# Fluorochrome Selection for Imaging Intraoperative Ovarian Cancer Probes

**DOI:** 10.3390/ph15060668

**Published:** 2022-05-26

**Authors:** Maria Grazia Perrone, Paola Vitale, Morena Miciaccia, Savina Ferorelli, Antonella Centonze, Roberta Solidoro, Cristina Munzone, Carmela Bonaccorso, Cosimo Gianluca Fortuna, Katrin Kleinmanns, Line Bjørge, Antonio Scilimati

**Affiliations:** 1 Department of Pharmacy-Pharmaceutical Sciences, University of Bari “Aldo Moro”, Via E. Orabona 4, 70125 Bari, Italy; mariagrazia.perrone@uniba.it (M.G.P.); paola.vitale@uniba.it (P.V.); morena.miciaccia@uniba.it (M.M.); savina.ferorelli@uniba.it (S.F.); antonella.centonze1@uniba.it (A.C.); roberta.solidoro@uniba.it (R.S.); 2 Department of Chemical Science, University of Catania, Viale Andrea Doria 6, 95125 Catania, Italy; cristinamunzone94@gmail.com (C.M.); bonaccorsoc@gmail.com (C.B.); cg.fortuna@unict.it (C.G.F.); 3 Centre for Cancer Biomarkers (CCBio), Department of Clinical Science, University of Bergen, Jonas Lies vei, 5021 Bergen, Norway; katrin.kleinmanns@uib.no (K.K.); line.bjorge@uib.no (L.B.); 4 Department of Obstetrics and Gynaecology, Haukeland University Hospital, 5021 Bergen, Norway

**Keywords:** mofezolac, COX-1 and COX-2 selective inhibitors, fluorescent probes, ovarian cancer, tumor-targeted imaging

## Abstract

The identification and removal of all gross and microscopic tumor to render the patient disease free represents a huge challenge in ovarian cancer treatment. The presence of residual disease is an independent negative prognostic factor. Herein, we describe the synthesis and the “in vitro” evaluation of compounds as cyclooxygenase (COX)-1 inhibitors, the COX-1 isoform being an ovarian cancer biomarker, each bearing fluorochromes with different fluorescence features. Two of these compounds *N*-[4-(9-dimethylimino-9H-benzo[a]phenoxazin-5-ylamino) butyl]-2-(3,4-bis(4-methoxyphenyl)isoxazol-5-yl)acetamide chloride (RR11) and 3-(6-(4-(2-(3,4-bis(4-methoxyphenyl)isoxazole-5-yl)acetamido)butyl)amino-6-oxohexyl)-2-[7-(1,3-dihydro-1,1-dimethyl-3-ethyl 2*H*-benz[e]indolin-2-yl-idene)-1,3,5-heptatrienyl]-1,1-dimethyl-3-(6-carboxilato-hexyl)-1*H*-benz[e]indolium chloride, **23** (MSA14) were found to be potent and selective inhibitors of cyclooxygenase (COX)-1 “in vitro”, and thus were further investigated “in vivo”. The IC_50_ values were 0.032 and 0.087 µM for RR11 and **23** (MSA 14), respectively, whereas the COX-2 IC_50_ for RR11 is 2.4 µM while **23** (MSA14) did not inhibit COX-2 even at a 50 µM concentration. Together, this represented selectivity index = 75 and 874, respectively. Structure-based virtual screening (SBVS) performed with the Fingerprints for Ligands and Proteins (FLAP) software allowed both to differentiate highly active compounds from less active and inactive structures and to define their interactions inside the substrate-binding cavity of *h*COX1. Fluorescent probes RR11 and **23** (MSA14), were used for preliminary near-infrared (NIR) fluorescent imaging (FLI) in human ovarian cancer (OVCAR-3 and SKOV-3) xenograft models. Surprisingly, a tumor-specific signal was observed for both tested fluorescent probes, even though this signal is not linked to the presence of COX-1.

## 1. Introduction

Ovarian cancer (OC) is the most aggressive gynecological cancer, and the 5-year survival rate is still <50%. This is due to delayed diagnoses, development of chemoresistance, and tumor evasion of the host’s immune responses.

OC of epithelial origin (EOC) constitutes 90% of all OC histological subtypes diagnosed. The remaining 10% are of germinal and stromal origin. Most patients with newly diagnosed EOC receive the same standard treatment, which consists of cytoreductive surgery together with adjuvant therapy consisting of first-line platinum-based chemotherapy. The addition of anti-angiogenetic agents and inhibitors of poly-ADP-ribose polymerase (PARP) molecules involved in DNA-repair processes as maintenance therapy is based on phenotypic selection criteria and the presence of mutations in the BRCA1 and BRCA2 genes, respectively. Overall, the surgical outcome remains a cornerstone in EOC treatment, presenting the most significant factor for prolonged survival [1,2].

However, besides the histological subtype, the extent of residual disease after surgery impacts patient outcomes [3]. The extent of residual disease is classified into complete cytoreduction (0 cm) and macroscopic residual disease, which is further subclassified into optimal (<1 cm) and suboptimal (>1 cm) cytoreduction [4]. The impact of complete debulking has been confirmed in many trials as the most important prognostic factor for the survival of EOC patients, and it has been suggested to increase the efficacy of subsequent drug therapies [5,6]. Clinical trials have also indicated that if macroscopic residual disease remains after surgery, the patient has no significant benefit compared to those who received suboptimal debulking, highlighting the need for better tools to help surgeons achieve complete resection.

Fluorescent probes are not yet routinely used to detect tissue lesions in oncology. However, over the past decade, there has been a notable growing interest in the development of fluorescent compounds that can enable surgeons to identify non-visible and/or non-palpable tumor masses in real time during cytoreductive surgery. In recent years, fluorescence image-guided surgery (FIGS) became of great interest for intraoperative detection of neoplastic bodies and real-time assessment of tumor borders to achieve complete tumor removal. Over the years, many efforts have been devoted to identifying targeted fluorescent imaging (FLI) compounds, which are characterized by high selectivity and sensitivity [7]. The use of this methodology would have a great impact on reducing disease recurrence, improving patients’ prognoses, and increasing overall survival (OS). As proof of this need, the U.S. Food and Drug Administration just approved Cytalux (pafolacianine targeting the folate receptor), an imaging drug intended to assist surgeons in identifying ovarian cancer lesions. The drug is designed to improve the ability to locate additional ovarian cancerous tissue that is normally difficult to detect during surgery. The drug is a diagnostic agent that is administered in the form of an intravenous injection prior to surgery [8].

Cyclooxygenase (COX) enzymes catalyze the conversion of arachidonic acid into potent mediators of the inflammatory syndrome triggered by a variety of stimuli. The prominent role in EOC of COX-1 over COX-2, is definitively ascertained, and several experimental data have shown COX-1, as a tumor-associated target in ovarian cancer supporting the idea that COX-1 could be a promising EOC biomarker, is suitable for EOC detection, and hence usable to guide EOC cytoreduction [9,10].

In this context, a new series of fluorescent compounds have been designed, synthesized, and pharmacologically characterized for their ability to inhibit and target COX. Fluorescent **23** (MSA 14), the most promising compound targeting “in vitro” COX-1, was compared to the reference compound RR11 [11] to access its possible use for ovarian cancer tumor-targeted FLI “in vivo”. SKOV-3 and OVCAR-3 subcutaneous (s.c.) xenograft models were imaged over time at different compound concentrations, showing tumor-specific uptake of **23** (MSA 14) and RR11.

## 2. Results and Discussion

### 2.1. Rationale behind the Design of the Novel Target Compounds

Novel compounds have been designed to target the COX-1 [12], especially in tumor cells and tissues where it is overexpressed [13,14,15,16,17]. From a chemical viewpoint, the molecules must carry at least three moieties: a mofezolac unit recognized through its carboxylic group by COX amino acid residues (Arg120, Tyr355, and Glu524) located at the entrance of the enzyme long hydrophobic channel that has the catalytic site on its top [18], a linker and a fluorochrome. Several fluorochromes were used to uncover the one endowed with the highest Stokes shift. Mofezolac was selected as the most potent and selective COX-1 inhibitor used in humans [19]. Flexible and rigid linkers were used to join mofezolac and the selected fluorochrome.

### 2.2. Chemistry

Most of the reaction conditions used to prepare the herein described target compounds for “in vitro”, “ex vivo”, and “in vivo” COX-1 detection use coupling reagents to form amide bonds. In other words, the carboxylic acids are “in situ” activated by the addition of HOBt to the reaction mixture to form an ester, before being mixed with the proper amine in the presence of the coupling reagent such as the EDC.

**Scheme 1:**
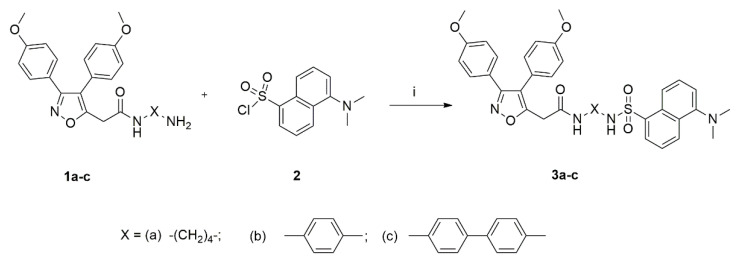
*Reagents and conditions*: (i) Et_3_N, anhydrous CH_2_Cl_2_, 25 °C, 2 h.

On the other hand, a chloride of the acid can be used (i.e., to prepare **3a–c**) [20]. Specifically, dansyl chloride (**2**) reacted with amines **1a–c** in the presence of Et_3_N; the latter was necessary to quench the HCl formed as a co-product during the reaction. This condition allowed us to prepare 2-[3,4-bis(4-methoxyphenyl)isoxazol-5-yl]-*N*-{4-[(5-dimethylaminonaphthalene)-1-sulfonamido]butyl}acetamide (**3a**), 2-[3,4-bis(4-methoxyphenyl)isoxazol-5-yl]-*N*-{4-[(5-dimethylaminonaphthalene)-1-sulfonamido]phenyl} acetamide (**3b**) and 2-[3,4-bis(4-methoxyphenyl)isoxazol-5-yl]-*N*-{4-[(5-dimethylaminonaphthalene)-1-sulfonamido]biphenyl} acetamide (**3c**) (Scheme 1).

**Scheme 2:**
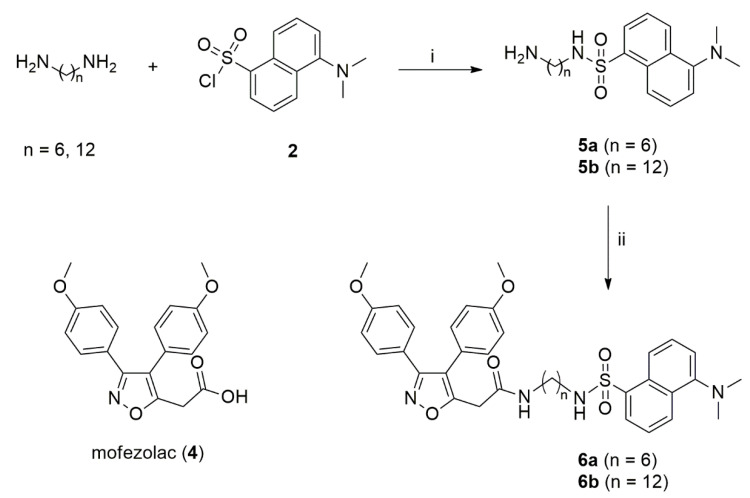
*Reagents and conditions*: (**i**) THF, 20h, r.t.; (**ii**) mofezolac (**4**), HOBt⋅H_2_O, DIEA, EDC⋅HCl, anhydrous CH_2_Cl_2_, r.t., 24 h.

We prepared **6a** and **6b** by reacting mofezolac (**4**), activated in situ being transformed into its HOBt ester, with **5a–b**. Then, in the presence of EDC, as coupling reagents, 2-[3,4-bis(4-methoxyphenyl)isoxazol-5-yl]-*N*-{4-[(5-dimethylaminonaphthalene)-1-sulfonamido]hexyl}acetamide (**6a)** and 2-[3,4-bis(4-methoxyphenyl)-5-yl]-*N*-{4-[(5-dimethylaminonaphthalene)-1-sulfonamido]dodecyl} acetamide (**6b**), respectively, were formed (Scheme 2).

*N*-(6-aminohexyl)-5-dansylsulfonamide (**5a**) and *N*-(12-aminododecyl)-5-dansylsulfonamide (**5b**) were obtained from the dansyl chloride (**2**) and 1,6-diaminohexane or 1,2-diaminododecane, respectively.

**Scheme 3:**
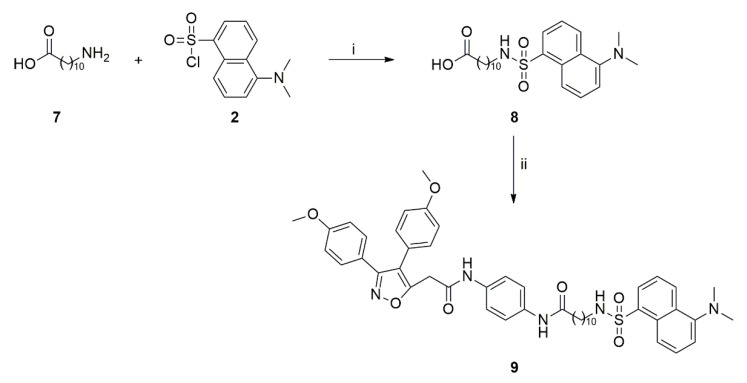
*Reagents and conditions*: (**i**) THF, 20 h, r.t.; (**ii**) HOBt⋅H_2_O, DIEA, EDC⋅HCl, anhydrous CH_2_Cl_2_, r.t., 16 h.

The synthesis of *N*-(4-(2-(3,4-bis(4-methoxyphenyl)isoxazol-5-yl)acetamido)phenyl)-11-((5-dimethylamino)naphthalene)-1-sulfonamido)undecamide (**9**) was accomplished by reacting 11-[(5-(dimethylaminonaphthalene)-1-sulfonamido]undecanoic acid (**8**) with *N*-(4-aminophenyl)-2-(3,4-bis(4-methoxyphenyl)isoxazol-5-yl)acetamide (**1b**) in the presence of HOBt/EDC. The intermediate **8** derived from the reaction of dansyl chloride (**2**) and 11-aminoundecanoic acid (**7**) (Scheme 3).

**Scheme 4:**
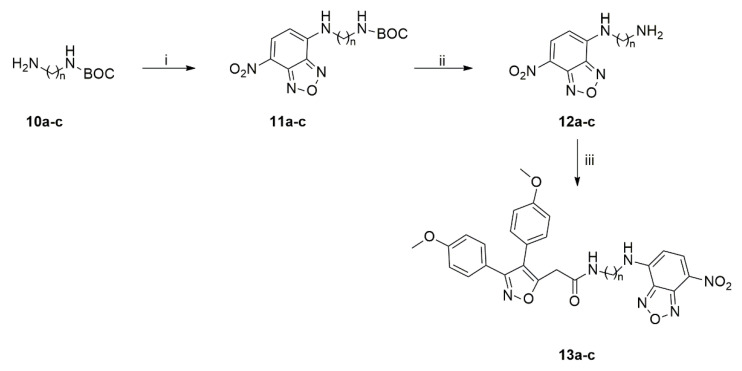
*Reagents and conditions:* (**i**) NBD-Cl, Et_3_N, anhydrous CH_2_Cl_2_, 25 °C, 16 h; (**ii**) TFA, 25 °C, 5 h; (**iii**) HOBt⋅H_2_O, DIEA, EDC⋅HCl, anhydrous CH_2_Cl_2_, r.t., 16 h.

Commercially available *N*-Boc-protected diamines **10a–c** reacted with 4-chloro-7-nitro-2,1,3-benzooxadiazole (NBD-Cl) to provide **11a–c,** that in turn were hydrolyzed to **12a–c**. Next, **11a–b** and **12a–b** were prepared and characterized, as previously reported [21,22], whereas **11c** and **12c** were prepared as depicted in Scheme 4. Their structural characterization is fully reported in the experimental section. Then, **12a–c** were reacted with mofezolac to give NBD derivatives **13a–c** (Scheme 4) [23,24].

**Scheme 5:**
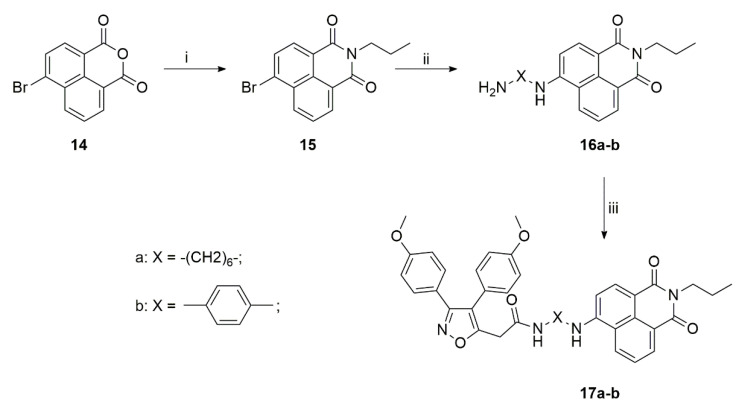
*Reagents and conditions:* (**i**) propylamine, EtOH, reflux, 16 h; (**ii**) 1,6-hexanediamine, 2-methoxyethanol **16a** or 1,4-phenylenediamine **16b**, Pd(OAc)_2_, K_2_CO_3_, anhydrous DMF, reflux, 16 h; (**iii**) mofezolac (**4**), HOBt⋅H_2_O, DIEA, EDC⋅HCl, anhydrous CH_2_Cl_2_, r.t., 16 h.

The synthesis of **17a–b** started with the conversion of 6-bromo-2-propyl-1*H*-benzo[de]isoquinoline-1,3(2*H*)-dione (**14**) into its N-propyl imide (**15**); in turn, reacting with 1,6-hexanediamine or 1,4-phenylenediamine afforded **16a–b** intermediates. The latter, in the presence of the coupling reagent and mofezolac, provided the two products **17a–b** (Scheme 5).

**Scheme 6:**
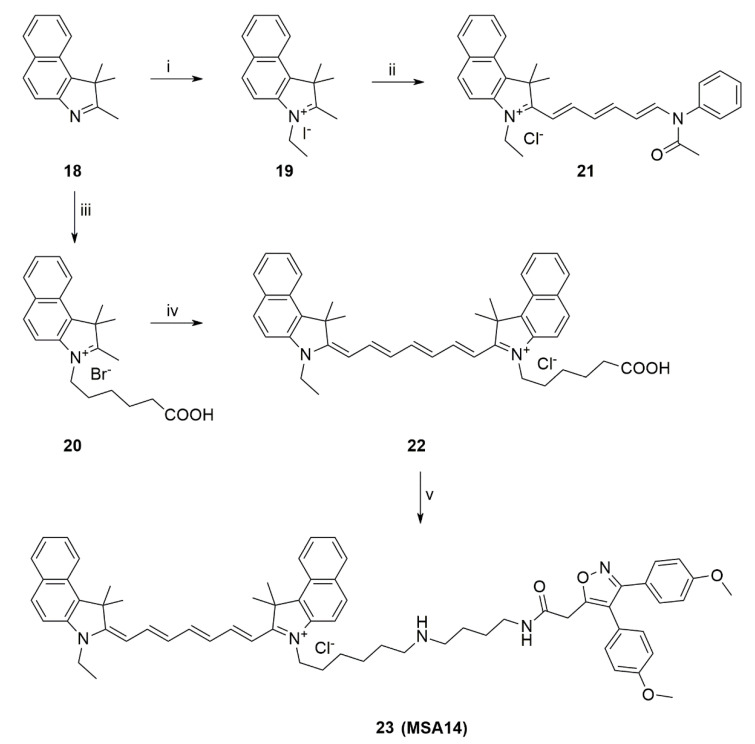
*Reagents and conditions:* (**i**) ethyl iodide, CH_3_CN, reflux, 4 days; (**ii**) glutaconaldehyde dianil hydrochloride, acetic anhydride, 100 °C, 1 h; (**iii**) 6-bromohexanoic acid, CH_3_CN, reflux, 4 days; (**iv**) **21**, pyridine, 40 °C, 30 min.; (**v**) 1a, HOBt⋅H_2_O, EDC⋅HCl, DIEA, anhydrous CH_2_Cl_2_, r.t., 16 h.

Then, **19** and **20** were obtained by reacting the commercially available 1,1,2-trimethylbenz[e]indole (**18**) with ethyl iodide or 6-bromohexanoic acid, respectively [25]. **19** reacted with glutaconaldehyde dianil hydrochloride providing **21** [25], in turn combined with **20** gave **22**. The target fluorescent probe **23** (**MSA14**) was obtained, coupling **22** and **1a** (Scheme 6).

#### Fluorescent Properties

The λ_abs_ and λ_em_, of the target compounds were measured in aqueous solution (phosphate-buffered saline solution, PBS) at the concentration of 10^−5^ M (Table 1).

The dansyl-bearing compounds **3a–c**, **6a–b** and **9** displayed λ_ex_ ~230–360 nm and λ_em_ in the range 492–514 nm, with a fairly large Stokes shift.

NBD-bearing compounds **13a–c** showed λ_abs_ between 482 and 495 nm and λ_em_ ranging from 550 to 564 nm, with the Stokes shift decreased, probably due to its well-known solvatochromic properties.

Compound **17a**, bearing the naphthalimide fluorophore, displayed λ_abs_ = 450 nm and λ_em_ = 546 nm. The excitation and emission wavelength values of **17b** were measured in EtOH because of its low solubility in PBS. Despite of the solvent, the Stokes shift was very low with this fluorophore.

The cyanine-bearing compound **23** (EtOH) showed a λ_abs_ and λ_em_ shifted towards higher wavelengths: 770 and 822 nm, respectively.

Overall, the measured λ_abs_ and λ_em_ for all the novel fluorescent molecules were in accordance with the properties conferred by the insertion of the specific fluorescent moiety. The fluorescent dyes used herein are known to be “environment sensitive”, since they display sensitivity to the local environment polarity. These pharmacodynamic properties combined with the optimal fluorescent spectroscopic properties, such as good Stokes shift and high fluorescence emission in aqueous and EtOH, make compounds **23** (MSA 14) and **RR11** [11] valuable tools to investigate COXs as targets “in vitro” and “in vivo” as well.

### 2.3. Biology

#### 2.3.1. Cyclooxygenase Catalytic Activity Inhibition Evaluation of the Novel Target Compounds

The novel fluorescent compounds were tested to evaluate their inhibitory activity and selectivity towards cyclooxygenases (Table 2) [26]. Regarding **3a–c** and **6a–b**, the presence of dansylsulfonamide moiety, as fluorophore, does not determine a change in the selectivity towards the cyclooxygenase isoforms. They remain selective COX-1 inhibitors. Additionally, 3a is almost inactive as an inhibitor towards both COXs. The nature of the linker does not affect the inhibitory potency of **3b–c** (COX-1 IC_50_ = 0.05 and 0.06 µM, respectively); it is important in **6a–b** (COX-1 IC_50_ = 12.0 and 0.1 µM, respectively).

In fact, **3a, 6a–b** showed an increase in potency as the length of the alkyl chain became longer, passing from an almost inactive **3a**, which determines only 45% COX-1 inhibition, to **6a**, which displays a 74% inhibition with a linker of six methylenes and a COX-1 IC_50_ value equal to 12 µM. The potency increases by an order of magnitude as the straight chain reaches the length of 12 carbon atoms (**6b**) (IC_50_ = 0.1 µM). In **6b,** the linker (twelve methylenes) confers a suitable flexibility to determine a greater inhibition potency, although the 55% of inhibition at compound concentration of 50µM is still low. The corresponding molecules with rigid linkers, phenyl (**3b**), and a benzidine (**3c**) showed a potency gain of an order of magnitude. The best result in terms of inhibition was obtained with **6b** (COX-1 IC_50_ = 0.06 µM and 71% inhibition). Bearing in mind that mofezolac (**4**), the most potent and selective COX-1 inhibitor, has COX-1 IC_50_ = 0.0079 µM [27,28,29], it seems that the presence of the dansyl moiety does not affect the selectivity of all new compounds because they remain as a COX-1 selective inhibitor, but with a reduced inhibitory potency. Among dansyl-bearing derivatives, **3c** remains the most potent inhibitor. When the fluorophore is bound to **3b** through a flexible linker with ten carbon atoms (**9**), the percentage of inhibition remains high (83%) but with a lower potency (IC_50_ = 0.7 µM). Unlikely dansyl-bearing compounds, the length of the linker does not determine any variation of the inhibitory activity of the NBD-bearing derivatives. On the contrary, the presence of this fluorophore completely cancels also the inhibitory activity induced by mofezolac. Specifically, all compounds **13a–c** result as inactive and non-selective. For the naphtylimide derivatives **17a–b**, a recovery of the selectivity in favor of the COX-1 isoform (IC_50_ = 0.08 µM and 0.15 µM for **17a** and **17b**, respectively) was observed, and was almost inactive towards COX-2 at 50µM final concentration.

In the dansyl derivatives **3a–c** and **6a–b** and naphthylamide derivatives **17a–b**, the dansylsulfonamide and naphthylimide carbonyls confer selectivity and potency towards COX-1, probably due to a certain electronic availability that allows them to interact with COX-1 active site, as revealed by the inspection of the binding poses in Fingerprints for Ligands and Proteins (FLAP) modeling studies. The cyanine-bearing compound 23 is still a potent and selective COX-1 inhibitor, with an IC_50_ = 0.087µM and 57% of inhibition, and is almost inactive towards COX-2 at 50µM final concentration.

#### 2.3.2. Computational Studies Based on the Fingerprints for Ligands and Proteins (FLAP) Algorithm

To ascertain the specific interactions established by the new fluorescent inhibitors with amino acids residues forming the *h*COX-1 binding site, structure-based virtual screening (SBVS) with the Fingerprints for Ligands and Proteins (FLAP) software [30] employing the recently published crystal structure of human cyclooxygenase (*h*COX)-1 (PDB ID: 6Y3C) as template, [31] was performed.

**Figure 1:**
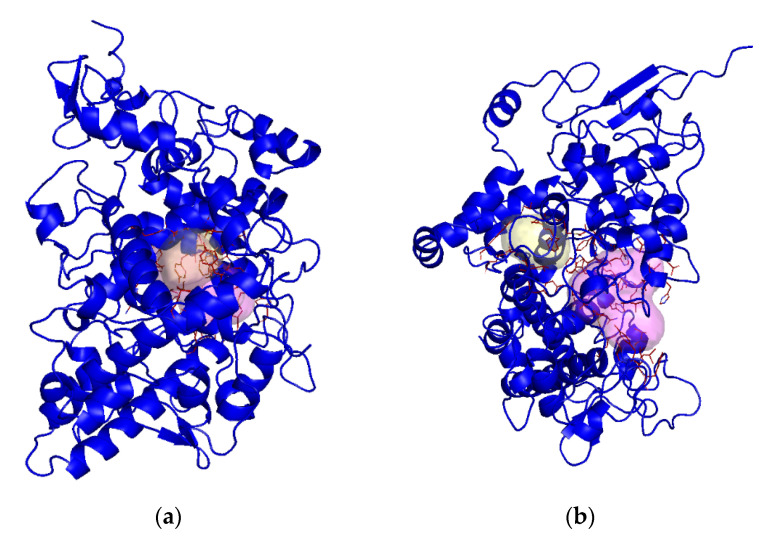
*h*COX-1 binding cavities calculated with the FLAP software: (**a**) front view and (**b**) side view Substrate-binding site (P1) is represented as a yellow surface. Heme catalytic site (P3) is represented as magenta surface.

In the first step of the computational study, the protein cavities (pockets) were calculated. Accordingly, five internal cavities in the structure of *h*COX-1 have been determined. As reported in Figure 1, it was possible to identify the substrate-binding site (P1) and the heme catalytic site (P3), in agreement with the analysis of *h*COX-1 crystal structures [31] [further details are in the Supporting materials (Figure S1)].

The SBVS approach allowed us to generate binding poses of the newly synthesized compounds in the substrate-binding cavity (P1) based on the similarity between their GRID fields; the FLAP binding pose for the reference compound **RR11** was also calculated in the new protein template and used as a reference (Figures S2 and S3). The FLAP calculated scores are reported in Table 3; the global similarity score (Glob-Sum) was used to rank the inhibitors.

Overall, the results reported in Table 3 shows that the SBVS procedure allows us to differentiate highly active compounds (IC_50_ < 0.1 μM, Glob-Sum > 3.0) from less active and inactive structures, the only exception being compound **13a**. It is noteworthy that structurally related compounds showing different inhibitor activity, i.e., **3a** and **3b**, gave reasons for different Glob-sum values, confirming the good correlation between this global parameter and the trend observed for the inhibitor activity.

**Figure 2:**
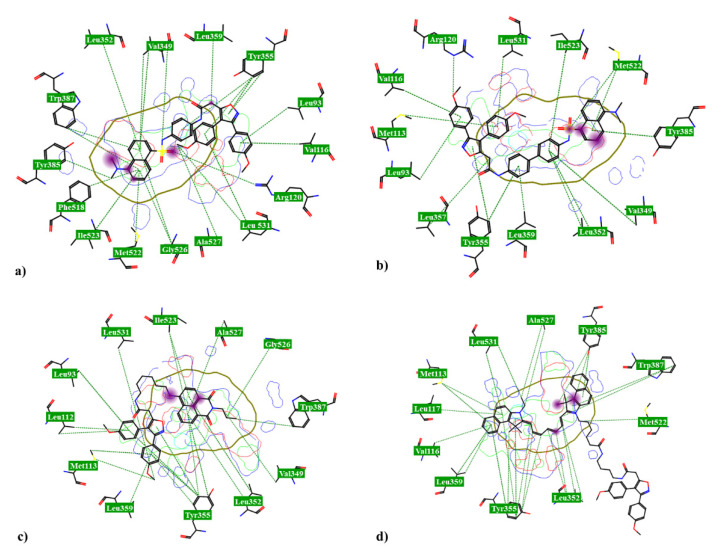
2D FLAP binding poses of **3b** (**a**), **3c** (**b**), **17a** (**c**) and **23** (**d**) inside the substrate-binding site of *h*COX-1 (PDB ID:6Y3C). Residues located in the *h*COX-1 binding site are highlighted in stick-mode: the most important amino acids are shown together with their respective numbers. Purple regions indicate strong interactions. Colored lines represent the area of hydrogen-bonding donor interactions (red), hydrogen-bonding acceptor interactions (blue), and hydrophobic interactions (green).

A close inspection at the binding poses, reported in Figure 2a–d and Figures S4–S7, for the most active compounds—namely **3b–c** and **17a–b**—revealed some common features:

(i) The fluorophore moieties are always located at the bottom of the P1 cavity and gave reasons for the strongest CH-π, S-π and π-π interactions with Tyr385, Trp387, Met522
(ii) The mofezolac units are placed in the outer region of the binding site realizing strong CH-π interaction with the external parallel α-helixes and strong π-π and H-bonds with Tyr 355 and Arg120, respectively, and lower cavity walls stabilize the inhibitors in the central region of the binding cavity.

Amongst the active compounds, the cyanine derivative **23** (Figure 2d) used the two fluorophore units for the interaction with both the entry and the bottom of the cavity; the mofezolac moiety showed negligible interaction, as it spanned behind the binding site but does not interfere with the heme catalytic site.

The binding pose obtained for **3a** and the NBD derivatives exhibited a similar arrangement of the different substrate moieties. However, no significant interaction occurred between the fluorophore units and the hydrophobic region of the binding cavity.

#### 2.3.3. Biodistribution of RR11 and 23 (MSA14) in OVCAR-3 Xenograft Models

Due to deeper tissue penetration of near-infrared (NIR) light, intraoperative imaging agents bearing NIR fluorophores are more valuable candidates for clinical translation than the shorter-wavelength fluorescent ligands. Consequently, two fluorescent probes, **RR11** and **23** (**MSA14**), were used for preliminary NIR FLI in OVCAR-3 bearing NSG mice.

**Figure 3:**
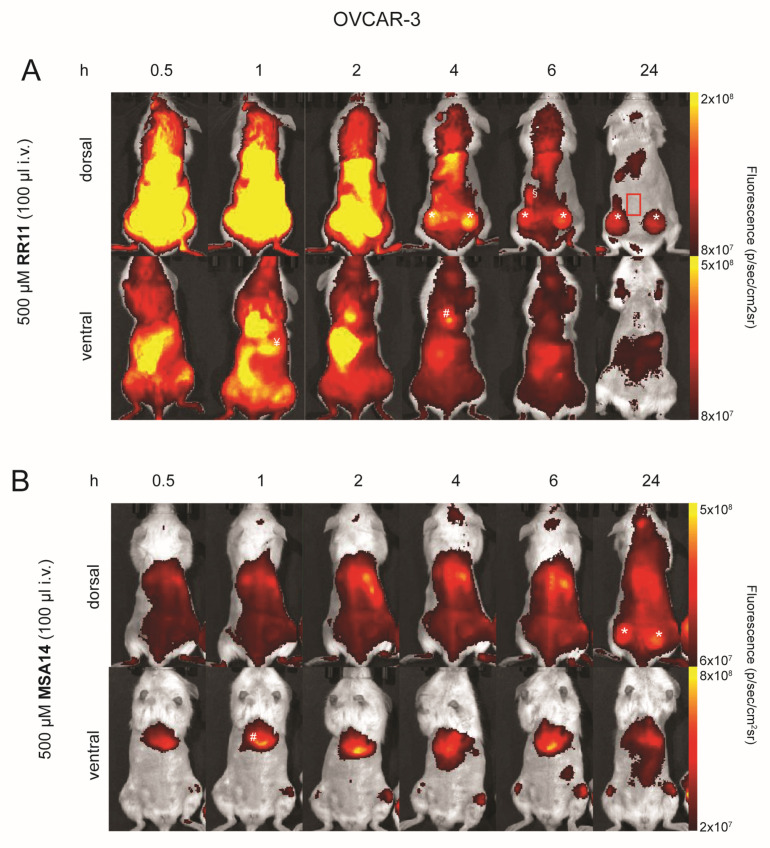
Biodistribution of RR11 and **23** (MSA14) in OVCAR-3 s.c. models: RR11 (**A**) and 23 (MSA14) (**B**) biodistribution was assessed by longitudinal “in vivo” whole body NIR FLI at six different time points (0.5, 1, 2, 4, 6, and 24 h) after intravenous (i.v.) injection of the fluorescent compounds. The fluorescence signal in the tumors (*), liver (#), kidney (§), stomach (¥), and background in the flank muscle (red square), presented by one representative mouse, were calculated with the IVIS spectrum living imaging software and expressed as radiance (p/s/cm2/sr).

To assess the capacity of both conjugates to visualize ovarian xenograft tumors “in vivo” and evaluate accumulation and clearance behavior of the conjugates, kinetic imaging was performed at six different time-points. Subcutaneously engrafted OVCAR-3 NSG mice were injected with 100 µL of 500 µM **RR11** or **23** (**MSA14**) intravenously into the tail vein (Figure 3). **RR11** imaged mice (*n* = 2) showed a high non-specific background signal at the first imaging time-points, with high signal intensities in kidneys and gastrointestinal tract (§, ¥, respectively, Figure 3A). Tumor-specific fluorescence was obtained 4, 6, and 24 h after **RR11** injection, with a mean tumor-to-background ratio (TBR) of 1.74 after 24 h. In OVCAR-3 bearing NSG mice (*n* = 3), injected with **23** (MSA14), high liver signals (ventral view, #) were obtained at all time points with high compound accumulation in the tumors at 24 h (*, Figure 3B). The best TBR of 1.44 was achieved after 24 h. Residual background fluorescence was predominantly observed in the spine and limbs as well as in the excretion organs.

**Figure 4:**
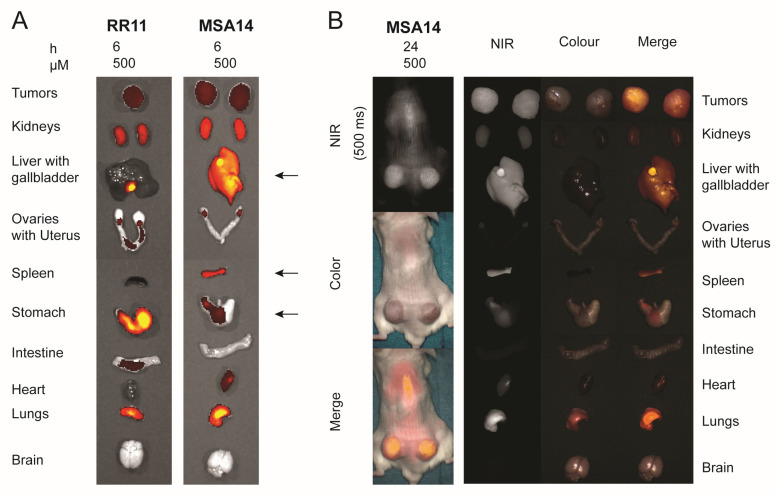
Organ biodistribution and intraoperative NIR FLI; (**A**) “Ex vivo” NIR FLI 6 h after 500 µM injection of **RR11** and **23** (**MSA14**) was performed to assess the compound accumulation in tumors and organs. (**B**) Representative intraoperative near-infrared (NIR), color and pseudo-colored fluorescent signal merge images of one **23** (**MSA14**) injected subcutaneously engrafted OVCAR-3 mouse in vivo and ex vivo.

Following the biodistribution over time “in vivo”, we assessed the accumulation of the compounds “ex vivo” at 6 h. In Figure 4A, **RR11**-induced tumor-specific signal, along with high fluorescence in kidneys, gallbladder, stomach, and lungs, can be observed. In contrast, “ex vivo” **23** (**MSA14**) FLI of tumor tissues and organs reveals distribution to kidneys, liver, spleen, and lungs with lower intensities in the stomach (Figure 4A). The higher gastrointestinal fluorescence signal in **RR11**-imaged mice can derive from auto-fluorescent alfalfa containing diet, which is acquired in the lower wavelength of **RR11**. The main differences between the accumulation and clearance behavior are marked with an arrow, indicating two different clearance patterns of the compounds through liver (**23, MSA14**) and kidney (**RR11**). Finally, the fluorescence of **23** (**MSA14**) was evaluated in a clinically compatible intraoperative setting using the FLARE^®^ system to assess the suitability of the COX-1 targeted NIR compound for FIGS and its potential clinical translation (Figure 4B). The “in vivo” and “ex vivo” intraoperative images confirm the results from Figure 3B and Figure 4A. Tumor-specific accumulation was observed in OVCAR-3 s.c. models. While the tumor fluorescence intensities were higher than those of kidneys, the fluorescence intensities in the gallbladder and lungs were the highest.

#### 2.3.4. Biodistribution of RR11 and **23** (MSA14) in OVCAR-3 Xenograft Models

**Figure 5:**
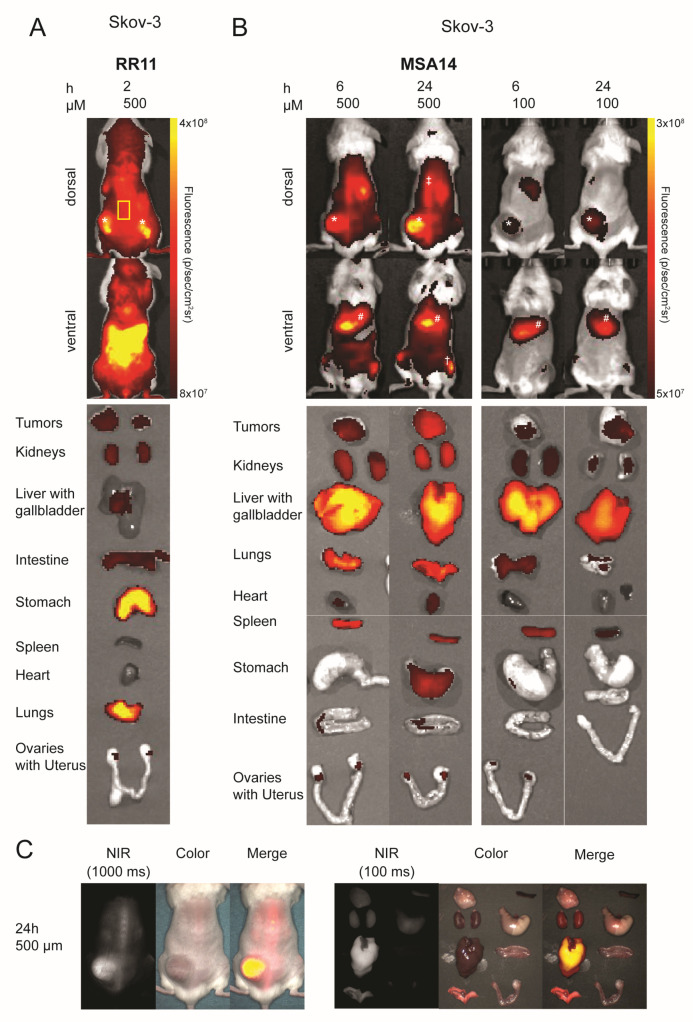
Biodistribution of **RR11** and **23** (**MSA14**) in SKOV-3 s.c. models. **RR11** (**A**) and **23** (**MSA14**) (**B**) were intravenously injected into n = 2 and n = 4 mice, respectively, and imaged at 2, 6, and 24 h with a dose of 500 and 100 µM. The fluorescence signal in the tumors (*), liver (#), limb (†), spine (‡) and background in the flank muscle (yellow square), is presented in one representative mouse. All animals were euthanized when indicated and “ex vivo” NIR FLI was performed. (**C**) 23 (MSA14) injected SKOV-3 mice were additionally imaged with the intraoperative imaging system “in vivo” and “ex vivo”. NIR exposure times are indicated in brackets.

In addition to OVCAR-3, the COX-1 low expressing SKOV-3 cell line was subcutaneously xenografted to evaluate the specificity of **RR11** and **23** (**MSA14**). **RR11** exerted high tumor fluorescence already two hours after injection (*n* = 2) with similar organ biodistribution as already observed in OVCAR-3 xenografts (TBR = 1.28; Figure 4A and Figure 5A). **23** (**MSA14**) was evaluated at 6 and 24 h, with a dose of 100 µM and 500 µM in *n* = 4 SKOV-3 models. Fluorescence signal in the tumor (average TBR = 1.49 at 6 h and 500 µM, 1.5 at 24 h and 500 µM, 1.23 at 6 h and 100 µM, 1.96 at 24 h and 100 µM) was observed in all four mice with unspecific signal in the liver, limbs, and spine (Figure 5B). However, “ex vivo” analysis revealed high signal in lungs (at high dose) and spleen as observed in OVCAR-3 models. Intraoperative imaging showed superior tumor signal at high dose and 1000 ms exposure time after 24 h (Figure 5C). However, the tumor fluorescence intensity was not sufficient to discriminate between healthy organs and malignant tissue at low exposure times of 100 ms “ex vivo”. The fluorescence signal in COX-1 low-expressing SKOV-3 tumors might be an indication of non-specific compound accumulation.

## 3. Materials and Methods

^1^H NMR and ^13^C NMR spectra were recorded on a Bruker 600 MHz or AGILENT 500 MHz spectrometer and chemical shifts are reported in parts per million (δ). The following abbreviations were used to explain the multiplicities: s—singlet; d—doublet; t—triplet; q—quartet; m—multiplet; quin—quintuplet; sext—sextet; sep—septet; b—broad. FT-IR spectra were recorded on a PerkinElmer 681 spectrophotometer. GC analyses were performed on a HP 6890 model, Series II, using a HP1 column (methyl siloxane; 30 m × 0.32 mm × 0.25 μm film thickness). Analytical thin-layer chromatography (TLC) was carried out on precoated 0.25 mm thick plates of Kieselgel 60 F254; visualization was accomplished by UV light (254 nm). Column chromatography was accomplished by using silica gel 60 with a particle size distribution 40–63 μm and 230–400 ASTM. GC-MS analyses were performed on HP 5995C model. High-resolution mass spectrometry (HRMS) analyses were performed using a Bruker microTOF QII mass spectrometer equipped with an electrospray ion source (ESI). Reagents and solvents were purchased from Sigma-Aldrich (Sigma-Aldrich, St. Louis, MO, USA) and used without any further purification. Full characterization data have been reported for both the newly synthesized compounds and the known compounds. All spectral data are consistent with the reported values. All compounds are >95% pure by HPLC carried out by Agilent Technology 1960 Infinity. **RR11** Rt = 3.490 min. (mobile phase: 20 mM NH_4_OAc (pH = 5.0)/CH_3_CN:20:80; stationary phase: ZORBAX ECLIPSE Plus C18, analytical 4.6 × 250 mm, 5-Micron); rate = 1 mL/min; **23** (MS14) Rt = 6.610 min. (Mobile phase: 20 mM NH_4_OAc (pH = 5.0)/CH_3_CN:10:90; stationary phase: ZORBAX ECLIPSE Plus C18, analytical 4.6 × 250 mm, 5-Micron); rate = 1 mL/min.

### 3.1. Synthesis of 2-[3,4-bis(4-methoxyphenyl)isoxazol-5-yl]-N-{4-[(5-dimethylaminonaphthalene)-1-sulfonamido]butyl or phenyl or biphenyl }acetamide (***3a–c***)

Et_3_N (0.65 mmol) was added to a stirred solution of *N*-(4-aminobutyl)-2-[3,4-bis(4-methoxyphenyl)isoxazole-5-yl]acetamide (**1a**) (0.53 mmol) [or *N*-(4-aminophenyl]-2-[3,4-bis(4-methoxyphenyl)isoxazole-5-yl]acetamide (**1b**) or *N*-(4′-amino [1,1-biphenyl]-4-yl)-2-[3,4-bis(4-methoxyphenyl)isoxazole-5-yl]acetamide (**1c**)] in anhydrous CH_2_Cl_2_ (10.0 mL). After 10 min., a solution of dansyl chloride (0.53 mmol) in anhydrous CH_2_Cl_2_ (10.0 mL) was added dropwise. The reaction mixture was stirred at room temperature for 2 h (TLC analysis: silica gel; AcOEt/hexane = 9/1 as mobile phase for **3a** and CHCl_3_/CH_3_OH = 9:1 as mobile phase for **3b–c**) and washed with aqueous 10% NaHCO_3_ (3 × 10 mL). Then, the solvent was removed under reduced pressure. The resulting brown semisolid underwent chromatography on silica gel and AcOEt/hexane = 9/1 as mobile phase for **3a** and CHCl_3_/CH_3_OH = 9.5:0.5 as mobile phase for **3b–c**.

#### 3.1.1. 2-[3,4-Bis(4-methoxyphenyl)isoxazol-5-yl]-N-{4-[(5-dimethylaminonaphthalene)-1-sulfonamido]butyl}acetamide (**3a**)

Forty percent yield, yellow-green solid. M.p. 69–71 °C. FT-IR (KBr): 3372, 3301, 2937, 2869, 1652, 1306, 1160, 836, 792 cm^−1^. ^1^H NMR (500 MHz, CDCl_3_, δ): 8.53 (d, 1H, J = 8.31 Hz, dansyl proton); 8.29 (d, 1H, J = 8.80 Hz, dansyl proton); 8.22 (d, 1H, J = 8.81 Hz, dansyl proton); 7.54–7.49 (m, 2H, dansyl protons); 7.38 (d, 2H, J = 8.81 Hz, mofezolac protons); 7.26–7.13 (m, 3H, 1H dansyl proton and 2H mofezolac protons); 6.97–6.87 (m, 2H, mofezolac protons); 6.88–6.73 (m, 2H, mofezolac protons); 5.92 (s, 1H, NHSO_2_); 5.02 (s, 1H, NHCO); 3.82 (s, 3H, OCH_3_); 3.80 (s, 3H, OCH_3_); 3.64 (s, 2H, CH_2_CO); 3.19 (q, 2H, J = 6.36, C*H*_2_NHSO_2_); 2.87-2.84 (m, 8H, N(CH_3_)_2_ and C*H*_2_NHCO);1.63-1.42 (m, 4H, (CH_2_)_2_). ^13^C NMR (125 MHz, CDCl_3_, δ): 166.7, 162.6, 161.1, 160.6, 159.5, 134.6, 131.0, 130.4, 129.8, 129.7, 129.6, 128.4, 123.2, 121.3, 121.0, 118.7, 117.5, 115.2, 114.4, 114.0, 55.3, 55.2, 45.4, 42.8, 39.2, 34.1, 29.7, 26.7, 26.4. HRMS (ESI) m/z calculated for [C_35_H_38_N_4_O_6_S + H]^+^: 643.2591; found 643.2586. ESI-MS-MS: 339.1338, 234.0572, 170.0958, 72.0814. (ESI) *m/z* [C_35_H_38_N_4_O_6_S + Na]^+^: 665.2410; found 665.2406. ESI-MS-MS: 665.2405, 532.1763, 370.1210, 102.1275.

#### 3.1.2. 2-[3,4-Bis(4-methoxyphenyl)isoxazol-5-yl]-N-{4-[(5-dimethylaminonaphthalene)-1-sulfonamido]phenyl}acetamide (**3b**)

Forty percent yield, yellow solid. M.p. 120–122 °C. FT-IR (KBr): 3281, 2930, 1671, 1609, 1512, 1292, 1250, 1144, 835, 790 cm^−1^. ^1^H NMR (500 MHz, CDCl_3_, δ): 8.49 (d, 1H, J = 8.32 Hz, dansyl proton); 8.31 (d, 1H, J = 8.80 Hz, Dansyl proton); 8.09 (d, 1H, J = 7.34 Hz, dansyl proton); 7.58 (t, 1H, J = 8.07, dansyl proton); 7.41–7.36 (m, 4H, 1dansyl proton, 2 mofezolac protons and NHCO); 7.23 (d, 2H, J = 8.80, mofezolac protons); 7.19 (d, 1H, J = 7.83, dansyl proton); 7.13 (d, 2H, J = 8.32, phenyl protons); 6.90 (d, 2H, J = 8.80, phenyl protons); 6.86–6.83 (m, 4H, mofezolac protons), 6.65 (s, 1H, NHSO_2_); 3.82 (s, 3H, OCH_3_); 3.80 (s, 3H, OCH_3_); 3.78 (s, 2H, CH_2_CO) 2.88 (s, 6H, N(CH_3_)_2_). ^13^C NMR (125 MHz, CDCl_3_, δ): 164.4, 161.7, 161.3, 160.7, 159.6, 134.9, 133.8, 132.7, 131.0, 130.9, 130.5, 129.8, 129.5, 128.7, 123.2, 123.1, 121.0, 120.8, 118.3, 117.8, 115.2, 114.5, 114.0, 55.3, 45.4, 35.1, 31.9, 29.7, 29.3, 22.7, 14.1. HRMS (ESI) *m/z* calculated for [C_37_H_34_N_4_O_6_S-H]^−^: 661.2119; found 661.2114. ESI-MS-MS: 661.2114 (100), 528.1584, 234.0589, 170.0972. (ESI) m/z [C_37_H_34_N_4_O_6_S + Na]^+^: 685.2097; found 685.2083. ESI-MS-MS: 685.2083 (100), 450.1423, 370.

#### 3.1.3. 2-[3,4-Bis(4-methoxyphenyl)isoxazol-5-yl]-N-{4-[(5-dimethylaminonaphthalene)-1-sulfonamido]biphenyl}acetamide (**3c**)

Twenty percent yield, yellow solid. M.p. 240–241 °C. FT-IR (KBr): 3439, 3013, 2977, 1645, 1325, 1163, 968, 761 cm^−1^. ^1^H NMR (300 MHz, CDCl_3_, δ): 8.53 (d, 1H, J = 8.57 Hz, dansyl proton); 8.35 (d, 1H, J = 8.57 Hz, dansyl proton); 8.19 (d, 1H, J = 7.91 Hz, dansyl proton); 7.62–7.36 (m, 9H, aromatic protons); 7.34–7.14 (m, 3H, aromatic protons); 7.10–6.78 (m, 7H, aromatic protons); 3.86 (s, 2H, CH_2_CO); 3.82 (s, 3H, OCH_3_); 3.80 (s, 3H, OCH_3_); 2.89 (s, 6H, N(CH_3_)_2_). ^13^C NMR (125 MHz, CDCl_3_, δ): 165.1, 162.4, 161.2, 160.6, 159.5, 136.6, 136.2, 135.7, 131.1, 130.3, 129.8, 129.5, 128.4, 127.3, 127.0, 125.1, 121.6, 121.2, 120.8, 120.6, 117.9, 114.4, 114.0, 59.5, 55.2, 46.0, 38.1, 35.0, 31.2, 29.7. HRMS (ESI) *m/z* calculated for [C_43_H_38_N_4_O_6_S-H]^−^: 737.2432; found 737.2430. ESI-MS-MS: 737.2430, 442.1239. (ESI) m/z [C_43_H_38_N_4_O_6_S + Na]^+^: 761.2410; found 761.2394. ESI-MS-MS: 761.2394, 527.1750, 442.3045.

### 3.2. Synthesis of 2-[3,4-bis(4-methoxyphenyl)isoxazol-5-yl]-N-{4-[(5-dimethylaminonaphthalene)-1-sulfonamido]hexyl or dodecyl}acetamide (***6a*** and ***6b***)

To a stirred solution of mofezolac (0.50 mmol) in anhydrous CH_2_Cl_2_ (15 mL), under argon atmosphere at 0 °C, we added HOBt monohydrate (0.57 mmol) and EDC hydrochloride (0.57 mmol). After 2 h, to this reaction mixture, a solution of *N*-(6-aminohexyl)-5-(dimethylamino)naphthalene-1-sulfonamide (**5a**) or *N*-(12-aminododecyl)-5-(dimethylamino)naphthalene-1-sulfonamide (**5b**) (0.57 mmol) and DIEA (0.57 mmol) in anhydrous CH_2_Cl_2_ (15 mL) was added, dropwise. The reaction mixture was allowed to warm to room temperature and stirred for 24 h (TLC analysis: silica gel; EtOAc/hexane = 9:1 as mobile phase for 6a and CHCl_3_/EtOAc = 7:3 as mobile phase for **6b**. Then, after removal of the solvent under reduced pressure, the resulting brown semisolid underwent chromatography on silica gel and EtOAc/hexane = 8:2 as mobile phase for 6a and CHCl_3_/EtOAc = 7:3 as mobile phase for **6b**.

#### 3.2.1. 2-[3,4-Bis(4-methoxyphenyl)isoxazol-5-yl]-N-{4-[(5-dimethylaminonaphthalene)-1-sulfonamido]hexyl}-acetamide (**6a**)

Seventy percent yield, yellow solid. M.p. 66–69 °C. FT-IR (KBr): 3372, 3301, 2933, 2869, 1660, 1610, 1574, 1395, 1314, 1160, 836, 792 cm^−1^. ^1^H NMR (500 MHz, CDCl_3_, δ): 8.53 (d, 1H, J = 8.31 Hz, dansyl proton); 8.29 (d, 1H, J = 8.80 Hz, dansyl proton); 8.23 (d, 1H, J = 8.31 Hz, dansyl proton); 7.55–7.51 (m, 2H, dansyl protons); 7.38 (d, 2H, J = 8.81 Hz, mofezolac protons); 7.18–7.13 (m, 3H, 1H dansyl proton and 2H mofezolac protons); 6.91–6.88 (m, 2H, mofezolac protons); 6.85–6.82 (m, 2H, mofezolac protons); 5.87 (s, 1H, NHSO_2_); 4.86 (t, 1H, J = 6.12 Hz, NHCO); 3.82 (s, 3H, OCH_3_); 3.80 (s, 3H, OCH_3_); 3.64 (s, 2H, CH_2_CO); 3.15 (q, 2H, J = 6.36, C*H*_2_NHSO_2_); 2.87–2.84 (m, 8H, N(CH_3_)_2_ and C*H*_2_NHCO); 1.38–1.27 (m, 4H, SNHCH_2_C*H*_2_(CH_2_)_2_C*H*_2_CH_2_NHCO); 1.20–1.06 (m, 4H, (CH_2_)_2_). ^13^C NMR (125 MHz, CDCl_3_, δ): 166.5, 162.7, 161.1, 160.6, 159.5, 134.9, 131.0, 130.3, 129.8, 129.6, 129.6, 128.3, 123.3, 121.3, 120.9, 118.8, 117.5, 115.2, 115.4, 114.0, 55.3, 55.2, 45.4, 43.0, 39.6, 34.2, 29.7, 29.3, 29.0, 25.9, 25.7. HRMS (ESI) *m/z* calculated for [C_37_H_42_N_4_O_6_S-H]^−^: 669.2746; found 669.2733. ESI-MS-MS: 669.2733, 374.1527, 264.0652. (ESI) m/z [C_37_H_42_N_4_O_6_S + Na]^+^: 693.2723; found 693.2725. ESI-MS-MS: 693.2725.

#### 3.2.2. 2-[3,4-Bis(4-methoxyphenyl)-5-yl]-N-{4-[(5-dimethylaminonaphthalene)-1-sulfonamido]dodecyl}acetamide (**6b**)

Twenty-two percent yield, brown solid. M.p. 126–129 °C. FT-IR (KBr): 3310, 2926, 2852, 1656, 1610, 1574, 1528, 1512, 1461, 1432, 1313, 1293, 1201, 1177, 1142, 835, 790 cm^−1^. ^1^H NMR (300 MHz, CDCl_3_, δ): 8.90–8.78 (m, 1H, dansyl proton); 8.58–8.44 (m, 1H, dansyl proton); 8.29 (d, 1H, J = 7.03 Hz, dansyl proton); 7.68–7.55 (m, 2H, dansyl protons); 7.42–7.35 (m, 3H, 1H dansyl proton and 2H mofezolac protons); 7.15 (d, 2H, J = 8.79 Hz, mofezolac protons); 6.91 (d, 2H, J = 8.79 Hz, mofezolac protons); 6.84 (d, 2H, J = 8.79 Hz, mofezolac protons); 5.57–5.55 (bs, 1H, NHCO); 4.56–4.52 (bs, 1H, NHCO); 3.83 (s, 3H, OCH_3_); 3.80 (s, 3H, OCH_3_); 3.68 (s, 2H, CH_2_CO); 3.30–3.19 (m, 2H, C*H*_2_NHSO_2_); 3.12–2.98 (m, 6H, N(CH_3_)_2_); 2.94–2.85 (m, 2H, C*H*_2_NHCO); 1.42–1.31 (m, 2H, C*H*_2_CH_2_NHSO_2_); 1.30–1.06 (m, 18H, (CH_2_)_9_). ^13^C NMR (125 MHz, CDCl_3_, δ): 166.4, 162.7, 161.1, 160.6, 159.5, 131.0, 129.8, 129.6, 128.1, 121.3, 121.0, 117.5, 114.4, 114.0, 60.4, 59.5, 55.3, 55.2, 45.7, 43.3, 40.0, 38.1, 34.2, 31.2, 29.7, 29.5, 29.4, 29.3, 29.2, 29.1, 28.9 26.7, 26.3, 22.7, 14.2, 14.1. HRMS (ESI) *m/z* calculated for [C_43_H_54_N_4_O_6_S-H]^−^: 753.3684; found 753.3683. ESI-MS-MS: 753.3683, 458.2502, 294.1160. (ESI) m/z [C_43_H_54_N_4_O_6_S + Na]^+^: 777.3661; found 777.3652. ESI-MS-MS: 777.3652.

### 3.3. Synthesis of N-(4-(2-(3,4-bis(4-methoxyphenyl)isoxazol-5-yl)acetamido)phenyl)-11-((5-dimethylamino)naphthalene)-1-sulfonamido)undecamide (***9***)

To a stirred solution of the commercially available (Sigma-Aldrich, Burlington, MA, USA) 11-((5-(dimethylaminonaphthalene)-1-sulfonamido)undecanoic acid (**8**) (0.336 mmol) in anhydrous CH_2_Cl_2_ (15 mL) kept under argon atmosphere and at 0 °C, HOBt monohydrate (0.50 mmol) and EDC hydrochloride (0.50 mmol) were added. After 2 h, a solution of *N*-(4-aminophenyl)-2-(3,4-bis(4-methoxyphenyl)isoxazol-5-yl)acetamide (**1b**) [32] (0.336 mmol) and DIEA (0.50 mmol) in anhydrous CH_2_CL_2_ (15 mL) was added to this solution dropwise. The reaction mixture was allowed to warm to room temperature, stirred for 24 h (TLC analysis: silica gel; EtOAc/hexane = 9:1 as mobile phase). After removal of the solvent under reduced pressure, the resulting brown semisolid underwent chromatography on silica gel and EtOAc/hexane = 9:1 as mobile phase. Nineteen percent yield, yellow solid. M.p. 140–143 °C. FT-IR (KBr): 3305, 2928, 2853, 1663, 1610, 1573, 1514, 1405, 1306, 1250, 1160, 835, 791 cm^−1^. ^1^H NMR (500 MHz, CD_3_OD, δ): 8.54 (d, 1H, J = 8.31 Hz, dansyl proton); 8.34 (d, 1H, J = 8.31 Hz, dansyl proton); 8.17 (d, 1H, J = 7.34 Hz, dansyl proton); 7.58–7.53 (m, 2H, dansyl protons); 7.51 (d, 2H, J = 8.81, aromatic protons); 7.46 (d, 2H, J = 8.80, aromatic protons); 7.35 (d, 2H, J = 8.81 Hz, mofezolac protons); 7.25 (d, 1H, J = 7.34, dansyl proton); 7.20 (d, 2H, J = 8.80, mofezolac protons); 6.93 (d, 2H, J = 8.32, mofezolac protons); 6.88 (d, 2H, J = 8.81, mofezolac protons); 3.86 (s, 2H, CH_2_CO); 3.79 (s, 3H, OCH_3_); 3.78 (s, 3H, OCH_3_); 2.86 (s, 6H, N(CH_3_)_2_); 2.83 (t, 2H, J = 6.85, C*H*_2_NHSO_2_); 2.33 (t, 2H, J = 7.34 Hz, NHCOC*H*_2_); 1.70-1.62 (m, 4H, SNHCH_2_C*H*_2_(CH_2_)_6_C*H*_2_CH_2_NHCO); 1.25–0.84 (m, 12H, (CH_2_)_6_). ^13^C NMR (125 MHz, CD_3_OD, δ): 161.0, 130.9, 129.8, 129.7, 129.4, 128.7, 127.6, 122.8, 120.2, 120.1, 119.2, 114.9, 113.9, 113.6, 54.4, 48.2, 44.4, 42.3, 36.5, 28.9, 28.8, 28.5, 26.0, 25.5. HRMS (ESI) *m/z* calculated for [C_48_H_55_N_5_O_7_S-H]^−^: 844.3743; found 844.3709. ESI-MS-MS: 844.3709, 549.3800. (ESI) m/z [C_48_H_55_N_5_O_7_S + Na]^+^: 868.3720; found 868.3720. ESI-MS-MS: 777.3652.

### 3.4. Synthesis of ***13a***–***c***

#### 3.4.1. Tert-Butyl(12-((7-nitrobenzo[c][1,2,5]oxadiazol-4-yl)amino)dodecyl)carbamate (**11c**)

To a stirred solution of mono-Boc-1,12-diaminododecane (**10c**) [24] (0.99 mmol) in anhydrous CH_2_Cl_2_ (20 mL), under argon atmosphere at r.t., Et_3_N (1.20 mmol) was added. After 10 min., a solution of 4-chloro-7-nitrobenzo[c][1,2,5]oxadiazole (0.99 mmol) in anhydrous CH_2_Cl_2_ (10 mL) was added dropwise. The reaction mixture was stirred at r.t. for 16 h (TLC analysis: silica gel; hexane/AcOEt = 8:2 as mobile phase) and then washed with H_2_O (3 × 10 mL). The organic layer was dried over anhydrous Na_2_SO_4_, filtered, and the solvent was distilled under reduced pressure. The resulting brown oil underwent chromatography on silica gel and hexane/AcOEt = 1/1 as mobile phase. Twenty-eight percent yield, yellow oil. ^1^H NMR (300 MHz, CDCl_3_, δ): 8.50 (d, 1H, J = 8.71 Hz, NBD proton); 6.25 (s, 1H, NHCO); 6.17 (d, 1H, J = 8.82 Hz, NBD proton); 4.48 (s, 1H, NHNBD); 3.48 (q, 2H, J = 6.64 Hz, C*H*_2_NHNBD); 3.13-3.04 (m, 2H, C*H*_2_NHBOC); 1.86–1.74 (m, 4H, CH_2_C*H*_2_(CH_2_)_8_C*H*_2_CH_2_); 1.34–1.22 (m, 25H, [CH_2_)_2_(C*H*_2_)_8_(CH_2_)_2_ and (CH_3_)_3_].

#### 3.4.2. N^1^-(7-Nitrobenzo[c][1,2,5]oxadiazol-4-yl)dodecane-1,12-diamine (**12c**)

To a stirred solution of tert-butyl(12-((7-nitrobenzo[c][1,2,5]oxadiazol-4-yl)amino)dodecyl)carbamate (**11c**) (0.27 mmol) in anhydrous CH_2_Cl_2_ (10 mL), trifluoroacetic acid (1.35 mmol) was added. The reaction mixture was stirred at r.t. for 5 h. After this time, 5% NaOH was added until pH = 14. Then, the reaction mixture was extracted with CH_2_Cl_2_ (3 × 10 mL). The organic layer, separated from the aqueous phase, was dried over Na_2_SO_4_, filtered, and the solvent was distilled under reduced pressure. The reaction crude product was used for the next reaction without any purification. Eighty-one percent yield, yellow oil. ^1^H NMR (300 MHz, CD_3_OD, δ): 8.52 (d, 1H, J = 8.79 Hz, NBD proton); 6.35 (d, 1H, J = 8.70 Hz, NBD proton); 3.60-3.41 (m, 2H, CH_2_NHNBD); 2.81-2.60 (m, 2H, CH_2_NH_2_); 1.80–0.82 (m, 20H, CH_2_(CH_2_)_10_CH_2_). HRMS (ESI) m/z calculated for [C_18_H_29_N_5_O_3_ + H]^+^: 364.2349; found 364.2345. ESI-MS-MS: 364.2345, 284.2120, 146.0713. (ESI) m/z [C_18_H_29_N_5_O_3_ + Na]^+^: 386.2164; found 386.2164. ESI-MS-MS: 386.2164, 259.0751, 225.0260, 182.1918, 134.0845, 89.0590. 

#### 3.4.3. 2-(3,4-Bis(4-methoxyphenyl)isoxazol-5-yl)-N-(4-((7-nitrobenzo[c][1,2,5]oxadiazol-4-yl)amino)butyl or hexyl or dodecyl)acetamide (**13a–c**)

To a stirred solution of mofezolac (0.073 mmol in anhydrous CH_2_Cl_2_ (10 mL), kept under argon atmosphere and at 0 °C, HOBt monohydrate (0.087 mmol) and EDC hydrochloride (0.087 mmol) were added. After 2 h, a solution of the amine (0.22 mmol) and DIEA (0.22 mmol) in anhydrous CH_2_Cl_2_ (10 mL) was added dropwise. The reaction mixture was allowed to warm to r.t. and stirred for 24 h (TLC analysis: silica gel; CHCl_3_/CH_3_OH = 9:1 as mobile phase for **13a**, EtOAc/hexane = 9:1 as mobile phase for **13b** and hexane/EtOAc = 6:4 as mobile phase for **13c**. Then, the solvent was distilled under reduced pressure to obtain a brown semisolid ass a reaction crude. The product was isolate by column chromatography by the same stationery and mobile phase used to monitor the reaction progress.

#### 3.4.4. 2-(3,4-Bis(4-methoxyphenyl)isoxazol-5-yl)-N-(4-((7-nitrobenzo[c][1,2,5]oxadiazol-4-yl)amino)butyl)acetamide (**13a**)

Twenty-one percent yield, yellow solid. M.p. 165 °C (dec.). FT-IR (KBr): 3411, 3313, 2919, 2850, 1676, 1611, 1512, 1305, 1251, 837, cm^−1^. ^1^H NMR (500 MHz, DMSO, δ): 9.53 (s, 1H, NHCO); 8.23 (t, 1H, J = 5.62 Hz, NHNBD), 8.48 (d, 1H, J = 8.81 Hz, NBD proton); 7.26 (d, 2H, J = 8.81 Hz, mofezolac protons); 7.15 (d, 2H, J = 8.81, mofezolac protons); 6.93–6.91 (m, 4H, mofezolac protons); 6.41 (d, 1H, J = 9.29 Hz, NBD proton); 3.74 (s, 3H, OCH_3_); 3.73 (s, 3H, OCH_3_); 3.58 (s, 2H, CH_2_CO); 3.55-3.42 (m, 2H, C*H*_2_NHNBD); 3.11 (t, 2H J = 6.85 Hz, C*H*_2_NHCO); 1.69–1.62 (m, 2H, C*H*_2_CH_2_NHCO); 1.54–1.47 (m, 2H, C*H*_2_CH_2_NHNBD). ^13^C NMR (125 MHz, DMSO, δ): 166.8, 166.7, 164.7, 160.6, 159.5, 145.6, 144.9, 144.6, 138.4, 131.4, 129.8, 128.5, 121.8, 121.3, 121.0, 116.9, 114.6, 114.6, 99.6, 79.6, 55.6, 55.5, 43.4, 38.8, 38.7, 33.3, 33.3, 26.9, 25.5, 22.2. HRMS (ESI) m/z calculated for [C_29_H_28_N_6_O_7_-H]^−^: 571.1939; found 571.1936. ESI-MS-MS: 571.1936, 250.0939, 116.0251. (ESI) *m/z* [C_29_H_28_N_6_O_7_ + Na]^+^: 595.1916; found 595.1911. ESI-MS-MS: 595.1911, 532.1940, 470.2042, 431.1551, 344.0884. 

#### 3.4.5. 2-(3,4-Bis(4-methoxyphenyl)isoxazol-5-yl)-N-(6-((7-nitrobenzo[c][1,2,5]oxadiazol-4-yl)amino)hexyl)acetamide (**13b**)

Thirty-three percent yield, orange semisolid. FT-IR (KBr): 3345, 2958, 2920, 2850,1654, 1614, 1583, 1297, 1260, 1177, 1099, 1023 cm^−1^. ^1^H NMR (300 MHz, CDCl_3_, δ): 8.44 (d, 1H, J = 8.79 Hz, NBD proton); 7.34 (d, 2H, J = 8.79 Hz, mofezolac protons); 7.16 (d, 2H, J = 8.79, mofezolac protons); 6.90 (d, 2H, J = 8.79 Hz, mofezolac protons); 6.81 (d, 2H, J = 8.79 Hz, mofezolac protons); 6.63 (bs, 1H, NHCO); 6.14 (d, 1H, J = 8.79 Hz, NBD proton); 5.90 (bs, 1H, CH_2_NH); 3.82 (s, 3H, OCH_3_); 3.79 (s, 3H, OCH_3_); 3.69 (s, 2H, CH_2_CO); 3.50–3.41 (m, 2H, C*H*_2_NHNBD); 3.33 (q, 2H, J = 6.25 Hz, C*H*_2_NHCO); 1.82-1.35 (m, 8H, (CH_2_)_4_). ^13^C NMR (125 MHz, CDCl_3_, δ): 166.81, 162.58, 161.23, 160.74, 159.55, 144.26, 143.94, 136.50, 134.75, 131.00, 129.69, 121.14, 120.69, 117.58, 114.41, 114.01, 98.51, 55.29, 55.25, 43.46, 39.37, 34.27, 29.69, 29.22, 28.19, 26.01, 25.79. HRMS (ESI) *m/z* calculated for [C_31_H_32_N_6_O_7_ − H]^−^: 599.2253; found 599.2215. ESI-MS-MS: 599.2215, 264.0660, 204.1138. (ESI) m/z [C_31_H_32_N_6_O_7_ + Na]^+^: 623.2230; found 623.2239. ESI-MS-MS: 623.2238, 560.2276, 459.1910, 344.0883. 

#### 3.4.6. 2-(3,4-Bis(4-methoxyphenyl)isoxazol-5-yl)-N-(12-((7-nitrobenzo[c][1,2,5]oxadiazol-4-yl)amino)dodecyl)acetamide (**13c**)

Thirty-two percent yield, yellow semisolid. FT-IR (KBr): 3418, 2923, 2851, 1652, 1614, 1582, 1513, 1297, 1253, 835, cm^−1^. ^1^H NMR (500 MHz, CDCl_3_, δ): 8.49 (d, 1H, J = 8.81 Hz, NBD proton); 7.38 (d, 2H, J = 8.81 Hz, mofezolac protons); 7.14 (d, 2H, J = 8.81 Hz, mofezolac protons); 6.90 (d, 2H, J = 8.32 Hz, mofezolac protons); 6.84 (d, 2H, J = 8.80 Hz, mofezolac protons); 6.36 (bs, 1H, NHCO); 6.16 (d, 1H, J = 8.80 Hz, NBD proton); 5.78 (bs, 1H, NHNBD); 3.82 (s, 3H, OCH_3_); 3.80 (s, 3H, OCH_3_); 3.68 (s, 2H, CH_2_CO); 3.49-3.45 (m, 2H, C*H*_2_NHNBD); 3.26 (q, 2H, J = 6.69 Hz, C*H*_2_NHCO); 1.82-1.76 (m, 2H, C*H*_2_CH_2_NHNBD); 1.50–1.24 (m, 18H, (CH_2_)_9_). ^13^C NMR (125 MHz, CDCl_3_, δ): 166.5, 162.6, 161.1, 160.6, 159.5, 144.3, 143.9, 136.5, 131.0, 129.7, 121.3, 120.9, 117.5, 114.4, 114.0, 98.4, 55.3, 44.0, 40.0, 34.2, 29.7, 29.3, 29.3, 29.2, 29.1, 29.0, 28.4, 26.8, 26.6. HRMS (ESI) *m/z* calculated for [C_37_H_44_N_6_O_7_ − H]^−^: 683.3191; found 683.3186. ESI-MS-MS: 683.3186, 636.3167, 362.2180, 294.1132. (ESI) m/z [C_37_H_44_N_6_O_7_ + Na]^+^: 707.3168; found 707.3159. ESI-MS-MS: 707.3156, 644.3193, 543.2813. 

### 3.5. 6-((6-Aminohexyl)amino)-2-propyl-1*H*-benzo[de]isoquinoline-1,3(2*H*)-dione (***16a***)

To a stirred solution of 6-bromo-2-propyl-1*H*-benzo[de]isoquinoline-1,3(2*H*)-dione (**15**) [33] (3.73 mmol) in 2-methoxyethanol (15.5 mL), kept under argon atmosphere, 1,6-diaminohexane (3.73 mmol) was added. The reaction mixture was heated under reflux for about 12 h in the dark. The reaction progress was monitored by TLC (CH_2_Cl_2_/CH_3_OH = 5:1). The reaction mixture was cooled to r.t. and then the solvent was distilled under reduced pressure. The reaction crude product was isolated by column chromatography (silica gel; CH_2_Cl_2_/CH_3_OH = 5:1 as mobile phase. Six percent yield, yellow oil. ^1^H NMR (300 MHz, CDCl_3_, δ): 8.45 (d, 1H, J = 7.03 Hz, aromatic proton); 8.36 (d, 1H, J = 8.00 Hz, aromatic proton); 8.01 (d, 1H, J = 7.90 Hz, aromatic proton); 7.54 (d, 1H, J = 7.60 Hz, aromatic proton); 6.45 (d, 1H, J = 7.60 Hz, aromatic proton); 5.78-5.60 (bs, 1H, NH); 4.09 (t, 2H, J = 7.32 Hz, C*H*_2_CH_2_CH_3_); 3.02–2.88 (m, 2H, NHC*H*_2_(CH_2_)_5_NH_2_); 2.71–2.30 (m, 4H, NH(CH_2_)_5_C*H*_2_ and NH_2_); 1.80-1.65 (m, 2H, CH_2_C*H*_2_CH_3_); 1.33–1.12 (m, 8H, NHCH_2_(C*H*_2_)_4_CH_2_); 0.98 (t, 3H, J = 7.32 Hz, CH_2_CH_2_C*H*_3_). HRMS (ESI) m/z calculated for [C_21_H_27_N_3_O_2_ + H]^+^: 354.2182; found 354.2186. ESI-MS-MS: 354.2186, 295.1410.

#### 6-((4-Aminophenyl)amino)-2-propyl-1*H*-benzo[de]isoquinoline-1,3(2*H*)-dione (**16b**)

To a stirred solution of 1,4-phenylenediamine (1.19 mmol) in anhydrous DMF (10 mL), kept under argon atmosphere, Pd(OAc)_2_ (0.47 mmol) and K_2_CO_3_ (0.93 mmol) were added. After 10 min, 6-bromo-2-propyl-1*H*-benzo[de]isoquinoline-1,3(2*H*)-dione (0.95 mmol) in anhydrous DMF (10 mL) was added to the reaction mixture, dropwise. The reaction mixture was heated under reflux for about 12 h. The reaction progress was monitored by TLC (CHCl_3_/CH_3_OH = 9.6/0.4). After the DMF distillation under reduced pressure, H_2_O was added, and the crude product was extracted with CH_2_Cl_2_ (3 × 10 mL). The organic layer was dried over anhydrous Na_2_SO_4_, filtered, and the solvent was distilled under reduced pressure. The resulting brown oil underwent column chromatography (silica gel, CHCl_3_/CH_3_OH = 9.6/0.4 as mobile phase). M.p. Thirty percent yield, yellow semisolid. ^1^H NMR (500 MHz, CD_3_OD, δ): 8.61 (d, 1H, J = 8.81 Hz, aromatic proton); 8.49 (d, 1H, J = 7.34 Hz, aromatic proton); 8.18 (d, 1H, J = 8.31 Hz, aromatic proton); 7.63 (t, 1H, J = 8.31 Hz, aromatic proton); 7.05 (d, 2H, J = 8.81 Hz, aromatic protons); 6.83 (d, 1H, J = 8.80 Hz, aromatic proton); 6.76 (d, 2H, J = 8.80 Hz, aromatic protons); 4.02 (t, 2H, J = 7.59 Hz, C*H*_2_CH_2_CH_3_); 1.71–1.59 (m, 2H, CH_2_C*H*_2_CH_3_); 0.92 (t, 3H, J = 7.34 Hz, CH_2_CH_2_C*H*_3_). HRMS (ESI) *m/z* calculated for [C_21_H_19_N_3_O_2_ − H]^−^: 344.1397; found 344.1395. ESI-MS-MS: 344.1395, 302.0920.

### 3.6. General Synthesis of ***17a*–*b***

To a stirred solution of mofezolac (0.17 mmol) in anhydrous CH_2_CL_2_ (5 mL), kept under argon atmosphere and at 0 °C, HOBt monohydrate (0.26 mmol) and EDC hydrochloride (0.26 mmol) were added. After 2 h, a solution of the amine (0.34 mmol) and DIEA (0.34 mmol) in anhydrous CH_2_Cl_2_ (15 mL) was added dropwise. The reaction mixture was allowed to warm to r.t. and stirred for 24 h. The reaction progress was monitored by TLC analysis (silica gel; EtOAc/hexane = 9:1 as mobile phase for compound **17a** and CHCl_3_/EtOAc = 7:3 as mobile phase for **17b**). Then, the solvent was distilled under reduced pressure and a brown semisolid was obtained as a crude reaction product. Column chromatography (silica gel; EtOAc/hexane = 9:1 as mobile phase for compound **17a** and CHCl_3_/EtOAc = 7:3 as mobile phase for compound **17b**) allowed us to isolate the expected product.

#### 3.6.1. Synthesis of 2-(3,4-Bis(4-methoxyphenyl)isoxazol-5-yl)-N-(6-((1,3-dioxo-2-propyl-2,3-dihydro-1*H*-benzo[de]isoquinolin-6-yl)amino)hexyl)acetamide (**17a**)

Sixty-eight percent yield, yellow solid. M.p. 110–111 °C. FT-IR (KBr): 3385, 3305, 2925, 2853, 1678, 1643, 1610, 1548, 1513, 1462, 1429, 1396, 1385, 1176, 1022, 833, 774 cm^−1^. ^1^H NMR (500 MHz, CDCl_3_, δ): 8.54 (d, 1H, J = 7.33 Hz, fluorescent moiety proton); 8.42 (d, 1H, J = 8.32 Hz, fluorescent moiety proton); 8.18 (d, 1H, J = 8.32 Hz, fluorescent moiety proton); 7.54 (t, 1H, J = 8.07 Hz, fluorescent moiety proton); 7.34 (d, 2H, J = 8.81 Hz, mofezolac protons); 7.15 (d, 2H, J = 8.81 Hz, mofezolac protons); 6.88 (d, 2H, J = 8.81 Hz, mofezolac protons); 6.80 (d, 2H, J = 8.81 Hz, mofezolac protons); 6.66 (d, 1H, J = 8.32 Hz, fluorescent moiety proton); 5.97 (t, 1H, J = 5.63 Hz, NHAr); 5.65 (t, 1H, J = 5.65 Hz, NHCO); 4.12 (t, 2H, J = 7.58 Hz, NCH_2_CH_2_CH_3_); 3.80 (s, 3H, OCH_3_); 3.77 (s, 3H, OCH_3_); 3.69 (s, 2H, CH_2_CO); 3.40–3.31 (m, 4H, NHCH_2_(CH_2_)_4_CH_2_NH_2_); 1.78–1.71 (m, 2H, NCH_2_CH_2_CH_3_); 1.58–1.46 (m, 4H, CH_2_(CH_2_)_2_CH_2_); 1.44–1.35 (m, 2H, (CH_2_)_2_CH_2_(CH_2_)_3_); 1.28–1.22 (m, 2H, (CH_2_)_3_CH_2_(CH_2_)_2_); 0.99 (t, 3H, J = 7.58 Hz, CH_2_CH_2_CH_3_). ^13^C NMR (125 MHz, CDCl_3_, δ): 166.6, 162.6, 160.7, 159.5, 149.5, 134.5, 131.0, 129.9, 129.7, 126.2, 124.6, 120.7, 117.6, 114.4, 114.0, 104.2, 55.3, 55.2, 43.0, 41.6, 39.4, 39.3, 34.3, 29.7, 29.3, 28.5, 26.1, 25.9, 21.4, 11.5. HRMS (ESI) m/z calculated for [C_40_H_42_N_4_O_6_ + Na]^+^: 697.3002; found 697.2999. ESI-MS-MS: 697.2999.

#### 3.6.2. Synthesis of 2-(3,4-Bis(4-methoxyphenyl)isoxazol-5-yl)-N-(4-((1,3-dioxo-2-propyl-2,3-dihydro-1*H*-benzo[de]isoquinolin-6-yl)amino)phenyl)acetamide (**17b**)

Thirty-six percent yield. M.p. 112–115 °C (orange solid). FT-IR (KBr): 3427, 3350, 3088, 3015, 2885, 1664, 1655, 1607, 1595, 1575, 1530, 1430, 1268, 1115, 880, 765 cm^−1^. ^1^H NMR (500 MHz, CDCl_3_, δ): 8.63 (d, 1H, J = 6.81 Hz, fluorescent moiety proton); 8.41 (d, 1H, J = 8.32 Hz, fluorescent moiety proton); 8.28 (d, 1H, J = 8.80 Hz, fluorescent moiety proton); 7.70 (t, 1H, J = 7.80 Hz, fluorescent moiety proton); 7.66–7.52 (m, 3H, mofezolac protons and ArNH); 7.42 (d, 2H, J = 8.81 Hz, mofezolac protons); 7.24–7.16 (m, 4H phenyl protons); 6.95 (d, 2H, J = 8.81 Hz, mofezolac protons); 6.86 (d, 2H, J = 8.81 Hz, mofezolac protons); 6.78–6.74 (m, 1H, fluorescent moiety proton); 5.30 (s, 1H, NHCO); 4.12–4.10 (m, 2H, NCH_2_CH_2_CH_3_); 3.89 (s, 2H, CH_2_CO); 3.84 (s, 3H, OCH_3_); 3.82 (s, 3H, OCH_3_); 1.82–1.74 (m, 2H, NCH_2_CH_2_CH_3_); 1.01 (t, 3H, J = 6.85 Hz, CH_2_CH_2_CH_3_). ^13^C NMR (125 MHz, CDCl_3_, δ): 164.6, 164.5, 163.9, 161.9, 161.3, 160.7, 159.7, 146.7, 136.5, 134.2, 131.4, 131.1, 129.9, 129.8, 126.2, 125.5, 123.5, 123.4, 121.7, 121.1, 120.7, 117.9, 114.5, 114.0, 113.4, 108.7, 55.3, 55.3, 41.7, 35.2, 21.4, 11.5. HRMS (ESI) m/z calculated for [C_40_H_34_N_4_O_6_ − H]^−^: 665.2398; found 665.2387. ESI-MS-MS: 665.2387, 532.1865, 370.1175. 

### 3.7. Synthesis of ***23*** (MSA14)

#### 3.7.1. 1,1,2-trimethyl-3-ethylbenz[e]indolium iodide (**19**)

To a stirred solution of the commercially available (Sigma-Aldrich, Burlington, MA, USA) 1,1,2-trimethylbenz[e]indole (**18**) (1.0 g, 4.8 mmol) in MeCN (40 mL), kept under argon atmosphere, ethyl iodide (1.17 g, 0.6 mL, 7.5 mmol) was added. The yellow reaction mixture was heated under reflux for two days. After TLC analysis (silica gel; EtOAc/hexane = 3:7 as mobile phase), more ethyl iodide (1.17 g, 0.6 mL, 7.5 mmol) was added to the reaction mixture, in turn heated under reflux for other two days. Then, the solvent was distilled under reduced pressure and to the resulting green semisolid, ethyl ether (50 mL) was added. The obtained solid was filtered and repeatedly washed with ethyl ether to obtain the expected product **19** as a green solid. Ninety-four percent yield, green solid. M.p. 231–233 °C. ^1^H NMR (300 MHz, CD_3_OD, δ): 8.33 (d, 1H, J = 8.79 Hz, aromatic proton); 8.24 (d, 1H, J = 9.37 Hz, aromatic proton); 8.16 (d, 1H, J = 8.20 Hz, aromatic proton); 8.03 (d, 1H, J = 8.79 Hz, aromatic proton); 7.84–7.77 (m, 1H, aromatic proton); 7.76–7.68 (m, 1H, aromatic proton); 4.85 (s, 3H, CH_3_); 4.68 (q, 2H, J = 7.61 Hz, C*H*_2_CH_3_); 1.84 (s, 6H, 2CH_3_); 1.63 (t, 3H, J = 7.61 Hz, CH_2_C*H*_3_). HRMS (ESI) *m/z* calculated for [C_17_H_20_N]^+^: 238.1590; found: 238.1583. ESI-MS-MS: 238.1583, 223.1342, 208.1110, 194.0956.

#### 3.7.2. 1,1,2-trimethyl-3-(6-carboxyl-hexyl)benz[e]indolium bromide (**20**)

To a stirred solution of 1,1,2-trimethylbenz[e]indole (**18**) (1.0 g, 4.8 mmol) in MeCN (40 mL), kept under argon atmosphere, 6-bromohexanoic acid (1.22 g, 6.24 mmol) was added. The reaction mixture was heated under reflux for seven days. Then, the solvent was distilled under reduced pressure, and ethyl ether (50 mL) was added to the resulting green semisolid. The obtained solid was filtered and repeatedly washed with ethyl ether to afford the product as a green solid. Seventy-one percent yield. M.p. 216–220 °C (dec.). ^1^H NMR (500 MHz, CD_3_OD, δ): 8.33 (d, 1H, J = 8.32, aromatic proton); 8.24 (d, 1H, J = 8.81, aromatic proton); 8.16 (d, 1H, J = 8.32, aromatic proton); 8.00 (d, 1H, J = 8.81, aromatic proton); 7.84–7.78 (m, 1H, aromatic proton); 7.74–7.68 (m, 1H, aromatic proton); 4.84 (s, 3H, CH_3_); 4.68 (t, 2H, J = 7.83 Hz, NC*H*_2_CH_2_); 3.35 (t, 2H, J = 7.10 Hz, C*H*_2_COOH); 2.18–2.02 (m, 2H, CH_2_C*H*_2_COOH); 1.84 (s, 6H, 2CH_3_); 1.75–1.68 (m, 2H, NCH_2_C*H*_2_); 1.62–1.55 (m, 2H, (CH_2_)_2_C*H*_2_(CH_2_)_2_). HRMS (ESI) *m/z* calculated for [C_21_H_26_NO_2_]^+^: 324.1958; found: 324.1959. ESI-MS-MS: 324.1959, 309.1724, 208.1120.

#### 3.7.3. 2-[6-(N-Phenyl-N-acetylamino)-1,3,5-hexatrienyl]-1,1-dimethyl-3-ethyl-1*H*-benz[e]indolium chloride (**21**)

A suspension of compound **19** (1.63 g, 4.5 mmol) in acetic anhydride (40 mL), and glutaconaldehyde dianil hydrochloride (1.28 g, 4.5 mmol), kept under argon atmosphere, was heated at 100 °C for 1 h. After cooling, the reaction mixture was poured into water and the obtained solid was filtered and repeatedly washed with water to give the product as a red solid. Sixty-nine percent yield. M.p. 162–165 °C. ^1^H NMR (300 MHz, CDCl_3_, δ): 8.19–7.92 (m, 5H, aromatic protons); 7.74–7.47 (m, 7H, 6H aromatic protons and 1H olefinic proton); 7.19–7.12 (m, 3H, olefinic protons); 7.03–6.94 (m, 1H, olefinic proton); 5.45–5.37 (m, 1H, olefinic proton); 4.89 (q, 2H, J = 7.13 Hz, C*H*_2_CH_3_); 1.98 (s, 6H, 2CH_3_); 1.57 (t, 3H, J = 7.13 Hz, CH_2_C*H*_3_). HRMS (ESI) *m/z* calculated for [C_30_H_31_N_2_O]^+^: 435.2431; found: 435.2431. ESI-MS-MS: 435.2431, 377.2007, 300.1744, 285.1509, 223.1348, 170.0960, 118.0649.

#### 3.7.4. 2-[7-(1,3-Dihydro-1,1-dimethyl-3-ethyl-1*H*-benz[e]indolin-2-yl-idene)-1,3,5-heptatrienyl]-1,1-dimethyl-3-(6-carboxilato-hexyl)-1*H*-benz[e]indolium inner salt (**22**)

A solution of compound **20** (0.50 g, 1.24 mmol) and **21** (0.58 g, 1.24 mmol) in pyridine (7 mL), under argon atmosphere, was stirred at 40 °C for 30 min. After cooling, the reaction mixture was poured into water and the obtained solid was filtered and repeatedly washed with 3N HCl to obtain a red solid. The product was isolated by column chromatography (silica gel; mobile phase: CHCl_3_/MeOH = 9:1, 8.5:1.5 and 8.2). Sixty percent yield, green solid. M.p. 229–232 °C. ^1^H NMR (500 MHz, CDCl_3_, δ): 8.13–8.09 (m, 2H, aromatic protons); 7.96–7.82 (m, 6H, 6H aromatic protons); 7.75–7.64 (m, 1H, aromatic proton), 7.62–7.55 (m, 3H, aromatic protons), 7.47–7.43 (m, 2H, 2CH), 7.35–7.33 (m, 2H, 2CH); 7.22–7.19 (m, 1H, CH); 6.86–6.68 (m, 1H, CH), 6.38–6.27 (m, 1H, CH); 4.29–4.12 (m, 4H, NC*H*_2_(CH_2_)_4_COOH), and NC*H*_2_CH_3_); 2.53 (t, 2H, J = 6.85, C*H*_2_COOH), 2.15–1.78 (m, 4H, CH_2_C*H*_2_CH_2_C*H*_2_CH_2_COOH); 1.99 (s, 6H, 2CH_3_), 1.97 (s, 3H, 2CH_3_), 1.48 (t, 3H, J = 7.33, CH_2_C*H*_3_). HRMS (ESI) *m/z* calculated for [C_43_H_47_N_2_O_2_]^+^: 623.3632; found: 623.3632. ESI-MS-MS: 623.3632, 400.2254, 308.1634, 222.1275. (ESI) m/z [C_43_H_46_N_2_O_2_ + Na]^+^: 645.3450; found 645.3455. ESI-MS-MS: 645.3455, 531.2754, 422.2081, 330.1459, 276.1737. 

#### 3.7.5. 3-(6-(4-(2-(3,4-Bis(4-methoxyphenyl)isoxazole-5-yl)acetamido)butyl)amino-6-oxohexyl)-2-[7-(1,3-dihydro-1,1-dimethyl-3-ethyl-2*H*-benz[e]indolin-2-yl-idene)-1,3,5-heptatrienyl]-1,1-dimethyl-3-(6-carboxilato-hexyl)-1*H*-benz[e]indolium chloride (**23**, **MSA14**)

2-[6-(*N*-Phenyl-*N*-acetylamino)-1,3,5-hexatrienyl]-1,1-dimethyl-3-ethyl-1*H*-benz[e]indolium chloride (**22**) (112 smg, 0.18 mmol) was solubilized in anhydrous CH_2_Cl_2_ (8 mL) in an argon-flushed, three-necked, round-bottom flask, with a dropping funnel and an argon inlet, equipped with a magnetic stirrer and an ice bath. HOBt^.^H_2_O (36 mg, 0.27 mmol) and EDC^.^HCl (51.7 mg, 0.27 mmol) were added to the reaction mixture, which was then stirred for 2h and kept at 0 °C. Soon after, *N*-(4-aminobutyl)-2-[3,4-bis(4-methoxyphenyl)isoxazol-5-yl]acetamide (**1a**) (73 mg, 0.18 mmol) and DIEA (0.045 mL, 0.27 mmol) were added, and the reaction mixture was stirred overnight at r.t. After the disappearance of the starting reagents, monitored by TLC (silica gel; mobile phase: CHCl_3_/MeOH = 9:1), the solvent was distilled under reduced pressure. The product was isolated by column chromatography (silica gel; mobile phase: CHCl_3_/MeOH = 9:1). Eighty-two percent yield, green solid. M.p. 220 °C (dec.). FT-IR (KBr): 3416, 2917, 2849, 1651, 1608, 1527, 1510, 1468, 1416, 1353, 1306, 1250, 1135, 1080, 1061, 1007, 921, 878 cm^−1^. ^1^H NMR (500 MHz, CDCl_3_, δ): 8.08–8.06 (m, 2H, aromatic protons); 7.93–7.88 (m, 4H, aromatic protons); 7.87–7.76 (m, 2H, aromatic protons); 7.66–7.62 (m, 1H, aromatic proton); 7.60–7.56 (m, 2H, aromatic protons); 7.47–7.34 (m, 6H, 1H aromatic proton, 4H olefinic protons and NH); 7.29–7.27 (m, 3H, 2H aromatic protons and 1H olefinic proton); 7.21 (d, 2H, J = 8.32 Hz, aromatic protons); 6.85 (d, 2H, J = 8.81 Hz, aromatic protons); 6.73 (d, 2H, J = 8.81 Hz, aromatic protons); 6.60 (t, 1H, J = 12.23 Hz, NH), 6.48 (d, 1H, J = 13.21 Hz, olefinic proton), 6.14 (d, 1H, J = 13.70 Hz, olefinic proton); 4.19 (t, 2H, J = 7.34 Hz, NC*H*_2_(CH_2_)_4_CO); 4.12–4.07 (m, 2H, NC*H*_2_CH_3_); 3.91 (s, 2H, CH_2_CO); 3.78 (s, 3H, OCH_3_); 3.73 (s, 3H, OCH_3_); 3.34-3.25 (m, 4H, NHC*H*_2_(CH_2_)_2_C*H*_2_NH); 2.41 (t, 2H, J = 7.10 Hz, N(CH_2_)_4_C*H*_2_CO); 1.94 (s, 6H, 2CH_3_); 1.93 (s, 6H, 2CH_3_); 1.84-1.72 (m, 4H, N(CH_2_)_3_C*H*_2_CH_2_CO and NH(CH_2_)_2_C*H*_2_CH_2_NH); 1.62-1.55 (m, 2H, NHCH_2_C*H*_2_(CH_2_)_2_NH); 1.42 (t, 3H, J = 7.10 Hz, CH_2_C*H*_3_); 1.28–1.20 (m, 4H, NCH_2_(C*H*_2_)_2_(CH_2_)_2_CO). ^13^C NMR (75 MHz, CDCl_3_, δ): 173.7, 173.5, 171.2, 167.3, 164.0, 160.6, 160.3, 159.2, 155.4, 150.8, 149.3, 139.5, 139.3, 133.8, 133.2, 131.9, 131.6, 131.2, 130.8, 130.5, 130.1, 130.0, 129.7, 128.3, 128.1, 127.7, 126.9, 126.2, 125.1, 124.7, 122.0, 121.7, 117.2, 114.2, 113.8, 110.8, 110.0, 104.6, 102.4, 77.4, 77.2, 77.0, 76.6, 55.2, 55.2, 51.0, 50.6, 44.9, 39.4, 39.2, 38.8, 36.3, 33.9, 29.7, 27.5, 26.3, 26.0, 25.4, 12.6. HRMS (ESI) *m/z* calculated for [C_66_H_72_N_5_O_5_]^+^: 1014.5528; found: 1014.5576. ESI-MS-MS: 1014.5576.

### 3.8. Fluorescence Property Measurements of the Target Compounds

Fluorescence spectra of all target compounds were determined in PBS and EtOH solutions (Table 1) with a Tecan Infinite 200 Microplate Reader. In all experiments, the excitation and the emission bandpass were set at λ = 5 nm. The emission spectra were obtained from λ = 280 to 850 nm, with excitation set at the appropriate excitation wavelength. Absorption spectra were recorded with a Shimadzu UV-1800 spectrophotometer.

### 3.9. Biology

Cell culture reagents were purchased from Euroclone (Milan, Italy). COX (ovine/human) inhibitor screening assay kit (Catalog No. 560131, Cayman Chemicals, Ann Arbor, MI, USA) was purchased from Sigma-Aldrich, Burlington, MA, USA. Mofezolac was prepared in our laboratory [29]. The other reagents were purchased from Sigma-Aldrich, Burlington, MA, USA.

#### 3.9.1. Cell Cultures

Ovarian cancer cell lines SKOV-3 and OVCAR-3 were purchased from American Type Culture Collection (ATCC) and cultured in RPMI culture medium containing 20% fetal bovine serum (FBS), 2 mM L-glutamine and 100 U/mL Penicillin-Streptomycin. OVCAR-3 cell culture cell culture medium was additionally supplemented with 0.01 mg/mL bovine insulin (cat# I6634, Sigma-Aldrich, St. Louis, MO, USA. Both cell lines were grown in the logarithmic phase at 37 °C in a 5%-CO_2_-humidified air.

#### 3.9.2. Cytotoxicity Study

Determination of SKOV-3 and OVCAR-3 cell growth was performed using the 3-(4,5-dimethylthiazol-2-yl)-2,5-diphenyltetrazolium bromide (MTT) assay [34]. On day 1, 20,000 cells/well were seeded into 96-well plates at a volume of 50 μL. On day 2, 50 μL of the various drug concentrations was added. In all the experiments, the drug solvent (DMSO) was added to each control to evaluate solvent cytotoxicity. After 48 h incubation time with drugs, MTT (10 µL, 0.5 mg/mL)) was added to each well, and after 3–4 h incubation at 37 °C, the supernatant was removed. The formazan crystals were solubilized using 100 μL of DMSO/EtOH (1:1), and the absorbance values at λ = 570 nm were determined on the Tecan Infinite 200 Microplate Reader.

#### 3.9.3. Cyclooxygenase Activity Inhibition

The COX-1/2 inhibition screening experiments were carried out according to the manufacturer’s instructions. The plate was incubated for 60 min at r.t. under shaking and read with the Tecan Infinite 200 Microplate Reader at λ = 405 nm.

The graph between percent inhibition of the enzyme and corresponding concentration of the compound provided IC_50_ values for the probe compounds.

#### 3.9.4. Subcutaneous Xenograft Models of Ovarian Cancer

Female NOD-scid IL2rγ^null^ (NSG) mice, aged 6 to 25 weeks were maintained under defined floral conditions and no more than five mice were housed in individually ventilated (HEPA-filtered air) cages, kept on a 12 h dark/night schedule at a constant temperature of 21 °C and at 50% relative humidity at the University of Bergen’s animal facility. Observations of body weight and general condition including activity levels, appearance, and food intake were monitored twice weekly, and humane endpoints were defined with the use of score sheets. When indicated, animals were euthanized according to institutional guidelines.

Single cell suspensions of SKOV-3 and OVCAR-3 ovarian cancer cells, cultured as previously described, were obtained by washing the adherent cells with PBS and detached with trypsin-EDTA for 3–5 min at 37 °C. For subcutaneous injection (s.c.), cells were resuspended in an ice-cold mixture of 100 µL per insulin syringe (3/10-cc with 30 g needle) comprising 50 μL saline, 25 µL medium, and 25 µL Matrigel (cat# 08-774-391, Corning Inc^®^, Fisher Scientific, Waltham, MA, USA) [35]. SKOV-3 and OVCAR-3 subcutaneous xenograft models were established through lateral flank injection of 5 × 10^6^ cells and 1 × 10^6^ cells, respectively, under 3.5% sevoflurane inhalation anesthesia (cat# 29960 SevoFlo, Zoetis, Louvain-la-Neuve, Belgium). Mice were imaged with RR11 and MSA14 when the caliper measurements reached 200–500 mm^3^ (Volume = (height × width × length × π)/6).

#### 3.9.5. Fluorescence Imaging (FLI)

“In vivo” and “ex vivo” FLI was performed with both COX-1 targeting compounds, the reference compound **RR11** and the novel **23** (**MSA14**), at different concentrations and time points. Prior to FLI, mice were shaved to avoid autofluorescence background signal. The desired concentration (100 µM, 500 µM) of the compound stock solutions in DMSO were freshly diluted with PBS, before 100 µL was injected intravenously into the tail vein. FLI was performed with the IVIS Spectrum In Vivo Imaging System with a λ_abs_ 640 ± 15 nm bandpass filter (BP), λ_em_ 680 ± 10 nm BP for **RR11**, and λ_abs_ 745 ± 15 nm BP, λ_em_ 820 ± 10 nm BP for **23** (**MSA14**), 0.5–24 h after small molecule administration. All scans were acquired with epi-illumination and auto exposure. Regions of interest (ROI) were manually gated around the tumor, organs, or the whole ventral and lateral positioned mouse and calculated using the Living image software (Perkin Elmer). The ROI of the muscle at the flank region was used to calculate the tumor-to-background (TBR) ratios.

A second intraoperative compatible imaging system (FLARE^®^, Curadel LLC, Nattick, MA, USA) was used to assess the clinical utility of **23** (**MSA14**). Images were acquired using the NIR channel #2 (λ_abs_760 ± 3 BP; λ_em_ 781 long pass filter) with an exposure time of 500 ms to 2 s and a gain of one. The color video channel has a 400–660 nm illumination source; exposure time was set to 8.5 ms. Both images were simultaneously acquired and merged as a pseudo-colored image. The imaging head was positioned at 24.5 cm working distance, resulting in a field of view (FOV) of 7,7 cm^2^ (no zoom).

### 3.10. Computational Methods

FLAP (fingerprints for ligands and proteins) [36,37] developed and licensed by Molecular Discovery Ltd. (Molecular Discovery, Borehamwood, United Kingdom) was employed to perform the virtual screening experiments, as reported in recent literature reports. [33,38].

The molecular interaction fields (MIFs) calculated by GRID [39] are employed in FLAP to describe both receptor and small molecules in terms of four-point pharmacophoric fingerprints. In the SBVS procedure, the GRID MIFs provide a complete description of both the ligands and the binding site (pocket), essentially in terms of shape (H), hydrophobic (DRY), H-bond acceptor (N1), and H-bond donor (O) properties of the pocket. The screening process involves the matching of the pockets and ligand quadruplets, which is quantitatively scored by considering the MIFs similarity. The Glob-Prod and Glob-Sum are two global scores that are produced by multiplying and summing all the scores of the individual probes.

## 4. Conclusions

Five different fluorochromes (dansyl, nitrobenzofuran-NBD, isoquinoline, cyanine, and nile-blue) were chosen as moieties of new compounds projected to target “in vivo” COX-1 overexpressed in the human ovarian cancer models. In particular, many such compounds were found to be potent and mostly highly selective COX-1 inhibitors, with their selective index ranging between 4 and 1000. COXs substrates and inhibitors are recognized by Arg120, Tyr355, and Glu524 located at the entry that gives access to the enzyme catalytic site. In this case, mofezolac moiety present in all twelve new compounds was expected to be recognized by the three amino acids (Arg120, Tyr355, and Glu524) and then to cross the long hydrophobic channel to reach Tyr385, responsible as a tyrosyl radical, to initiate the reaction that converts arachidonic acid into PGH_2_ (precursor of prostaglandins, thromboxane and prostacyclin). Instead, this portion remains outside the catalytic zone while the fluorochrome is inside, as shown in the FLAP measurements. **RR11** and **23** (**MSA14**) “in vivo” results show a high tumor-unspecific signal, which causes overall low TBRs. COX-1 is constitutively expressed in many organs, e.g., liver, spleen, intestines, and bone marrow, so the signal could be a result of mouse and human COX-1 cross reactivity. Therefore, COX-1 expression in ovarian cancer cell lines might not be specific enough, which renders both compounds unsuitable for FIGS. Further investigations are underway to better address the problem of fluoresce-guided surgery in ovarian cancer and to identify the real target of the newly prepared compounds.

## Figures and Tables

**Table 1:** Fluorescence properties of the new compounds ^a^.

Compound	λ_abs_	λ_em_	Stokes Shift
**3a**	230	494	264
**3b**	360	514	154
**3c**	287	504	217
**6a**	248 ^b^	521 ^b^	273
**6b**	270	492	222
**9**	274	496	222
**13a**	482	564	82
**13b**	490	554	64
**3a**	230	494	264
**3b**	360	514	154
**13c**	495	550	55
**17a**	450	546	96
**17b**	450 ^b^	542 ^b^	92
**23 (MSA14)**	260/770 ^c^	318/822 ^c^	58/52 ^c^
**RR11**	610 ^b^	672 ^b^	62

^a^ All the measurements were performed in PBS solution, unless otherwise specified; ^b^ fluorescence spectrum was recorded in ethanol (10^−5^ M) due to its low solubility in PBS; ^c^ fluorescence spectrum in ethanol (10^−4^ M).

**Table 2:** COXs inhibitory activity (IC_50_) of the newly synthesized compounds.

Compound	COX-1 IC_50_, µM (% inhib. ^a^)	COX-2 IC_50_, µM (% inhib. ^a^)	SI ^b^
**3a**	>50 (45)	>50	-
**3b**	0.05 (44)	>50	1000
**3c**	0.06 (71)	>50	833
**6a**	12.0 (74)	>50	4
**6b**	0.1 (55)	>50	500
**9**	0.7 (83)	˃50	71
**13a**	>50 (0)	>50	-
**13b**	>50 (26)	>50	-
**13c**	>50 (28)	>50	-
**17a**	0.08 (51)	>50	625
**17b**	0.15 (49)	>50	333
**23 (MSA14)**	0.087 (57)	>50	874
**RR11** (reference compound)	0.032 (83)	2.4 (64)	72

^a^ In brackets the percentage (%) inhibition measured at 50 µM compound final concentration is reported. ^b^ SI (selectivity index) = IC_50_ COX-2/IC_50_ COX-1.

**Table 3:** Scores obtained for the SBVS of the new inhibitors in *h*COX-1 crystal structure (PDB ID: 6Y3C) performed with the FLAP software.

Ligand	Distance ^a^	H ^b^	N1 ^c^	DRY ^d^	O ^e^	Glob-Sum ^f^
**3a**	6.827	0.883	0.171	1.979	0.340	2.843
**3b**	6.051	0.876	0.258	2.331	0.362	3.108
**3c**	6.678	0.834	0.186	2.237	0.328	3.073
**6a**	7.005	0.800	0.180	2.007	0.439	2.917
**6b**	6.096	0.854	0.222	2.107	0.395	2.875
**9**	7.145	0.766	0.175	2.164	0.340	2.935
**13a**	6.229	0.900	0.202	1.904	0.528	2.972
**13b**	7.371	0.850	0.181	1.897	0.385	2.702
**13c**	7.503	0.848	0.123	1.827	0.356	2.671
**17a**	6.670	0.870	0.168	2.132	0.366	2.911
**17b**	6.648	0.866	0.181	2.123	0.384	2.903
**23**	7.959	0.615	0.153	2.460	0.356	3.042
**RR11**	6.098	0.873	0.201	2.455	0.301	3.262

^a^ Distance score represents the overall similarity between the ligand and the template structures; ^b^ H (hydrogen) probe describes the shape of the interaction; ^c^ N1 (amide NH) probe describes the hydrogen bond donor character of interaction; ^d^ DRY (hydrophobic) probe describes the hydrophobic character of interaction; ^e^ O (carbonyl oxygen) probe describes the hydrogen bond acceptor character of interaction; ^f^ global sum is calculated by summing all the scores of the individual probes together.

## Data Availability

Data is contained within the article and supplementary material.

## Supplementary Materials

The following supporting information can be downloaded at: https://www.mdpi.com/article/10.3390/ph15060668/s1, Figures S1–S7: 2D and 3D binding mode and of selected target compounds.
