# Supplementary material for: Fluorochrome Selection for Imaging Intraoperative Ovarian Cancer Probes
Source: Pharmaceuticals (Basel). 2022 May 26;15(6):668. doi: 10.3390/ph15060668 (PMC9230671; doi:10.3390/ph15060668)
Supplement: Supplementary file 1 [file pharmaceuticals-15-00668-s001.zip › pharmaceuticals-1685022-supplementary.pdf]

Supplementary Material

Fluorochrome selection for imaging intraoperative ovarian cancer probes

Maria Grazia Perrone[1], Paola Vitale[1], Morena Miciaccia[1], Savina Ferorelli[1], Antonella Centonze[1], Roberta Solidoro[1], Cristina Munzone[2], Carmela Bonaccorso[2], Cosimo G. Fortuna[2], Katrin Kleinmanns[3], Line Bjørge[3, 4], Antonio Scilimati1[1]*

[1] Department of Pharmacy - Pharmaceutical Sciences, University of Bari "Aldo Moro", Via E. Orabona 4, 70125 Bari, Italy; mariagrazia.perrone@uniba.it (M.G.P.); paola.vitale@uniba.it (P.V.); morena.miciaccia@uniba.it (M.M.); savina.ferorelli@uniba.it (S.F.); antonella.centonze1@uniba.it (A.C.); roberta.solidoro@uniba.it (R.S.)
[2] Department of Chemical Science, University of Catania, Viale Andrea Doria 6, 95125 Catania, Italy; cristinamunzone94@gmail.com (C.M.); bonaccorsoc@gmail.com (C.B.); cg.fortuna@unict.it (C.G.F.)
[3] Department of Clinical Science and Centre for Cancer Biomarkers (CCBio), University of Bergen, Jonas Lies vei, 5021 Bergen, Norway; katrin.kleinmanns@uib.no (K.K.); line.bjorge@uib.no (L.B.)
[4] Department of Obstetrics and Gynaecology, Haukeland University Hospital, 5021 Bergen, Norway; line.bjorge@uib.no (L.B.)
* Correspondence: antonio.scilimati@uniba.it (A.S.)

Content:

1. **^{1}H NMR and ^{13}C NMR of the target compounds.**
2. **HPLC chromatograms of RR11 and 23 (MSA14).**
3. **FLAP S1-S7 figures of selected target compounds.**

1. **^{1}H NMR and ^{13}C NMR of the target compounds.**

2-[3,4-Bis(4-methoxyphenyl)isoxazol-5-yl]-N-{4-[(5-dimethylaminonaphthalene)-1-sulfonamido]butyl}acetamide (3a)

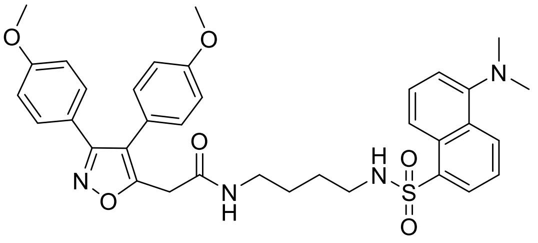

3a ^{1}H-NMR (500 MHz, CDCl$_3$)

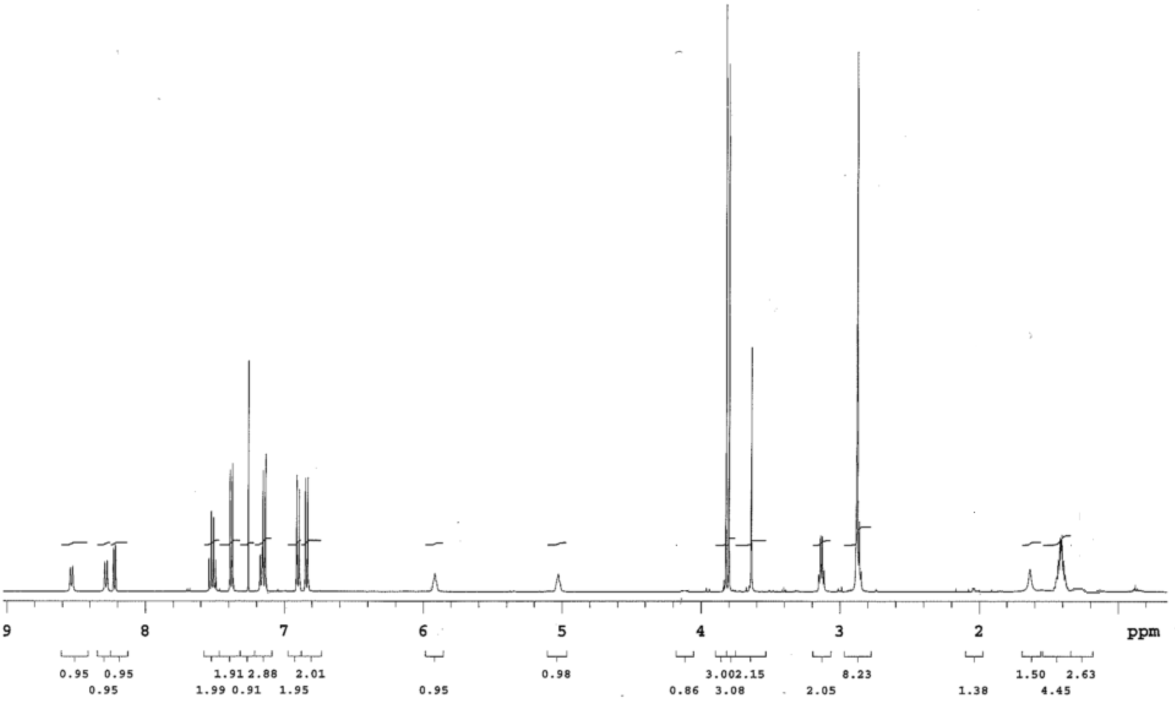

3a ^{13}C-NMR (125 MHz, $CDCl_3$)

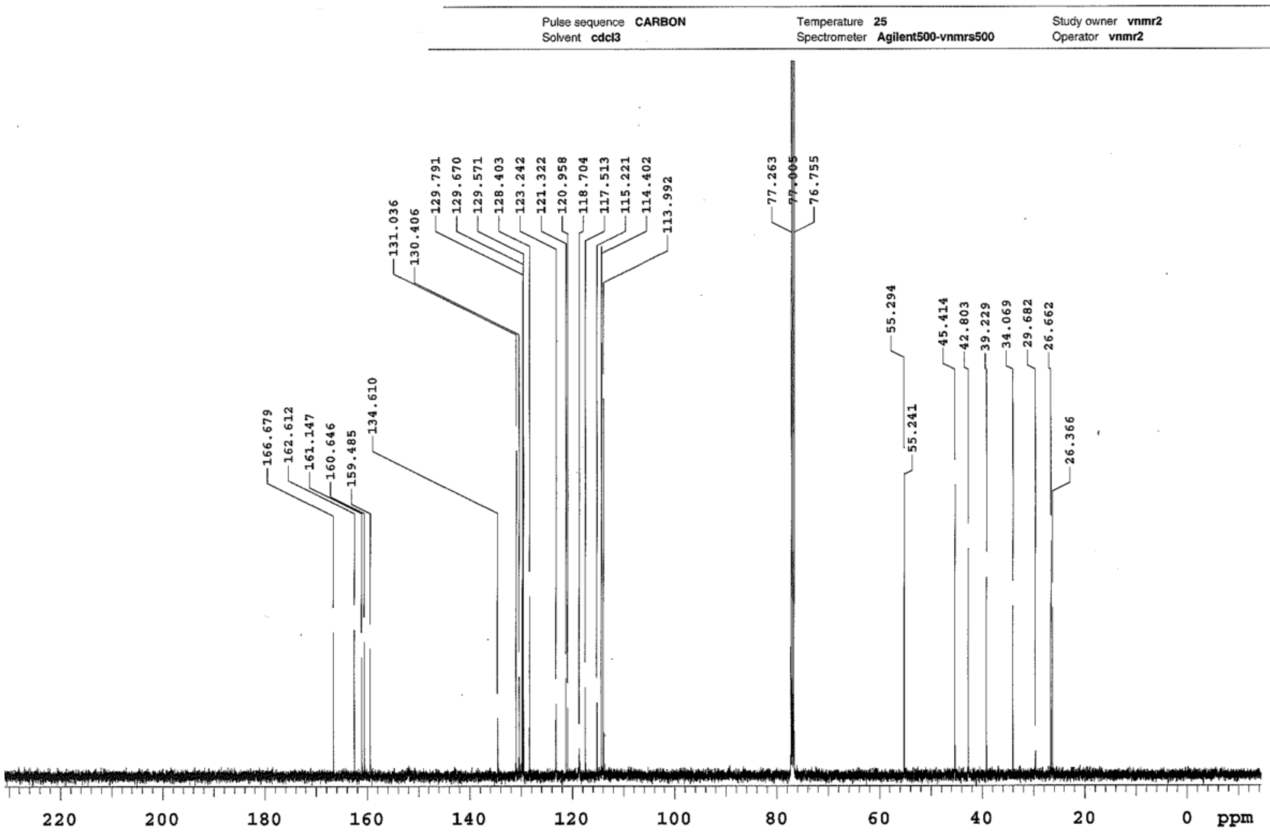

2-[3,4-Bis(4-methoxyphenyl)isoxazol-5-yl]-N-{4-[(5-dimethylaminonaphthalene)-1-sulfonamido]hexyl}-acetamide (6a)

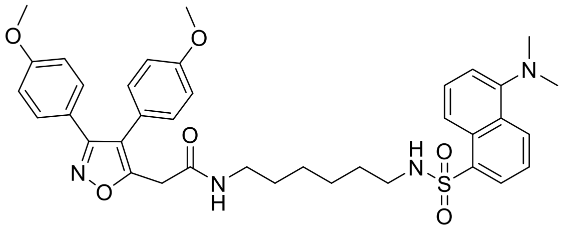

6a ^{1}H-NMR (500 MHz, $CDCl_3$)

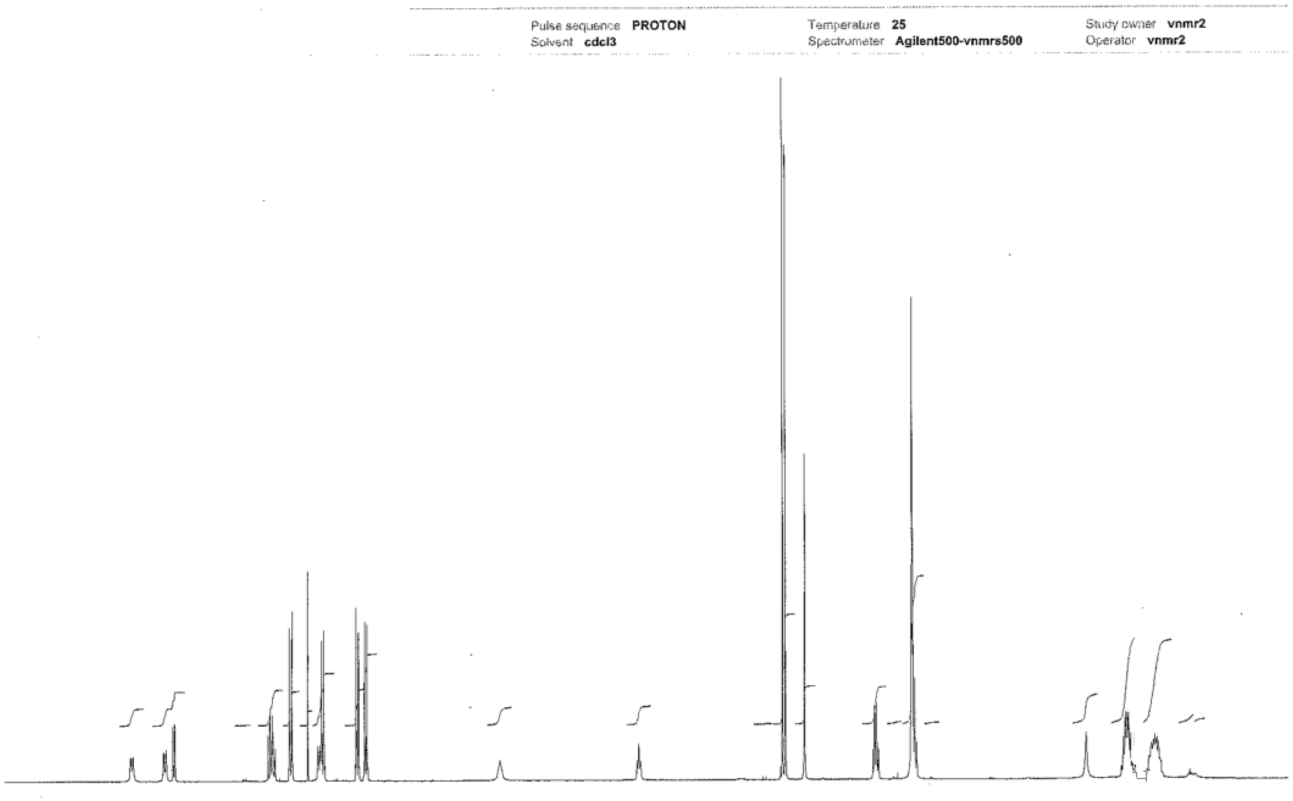

6a ^{13}C-NMR (125 MHz, CDCl$_3$)

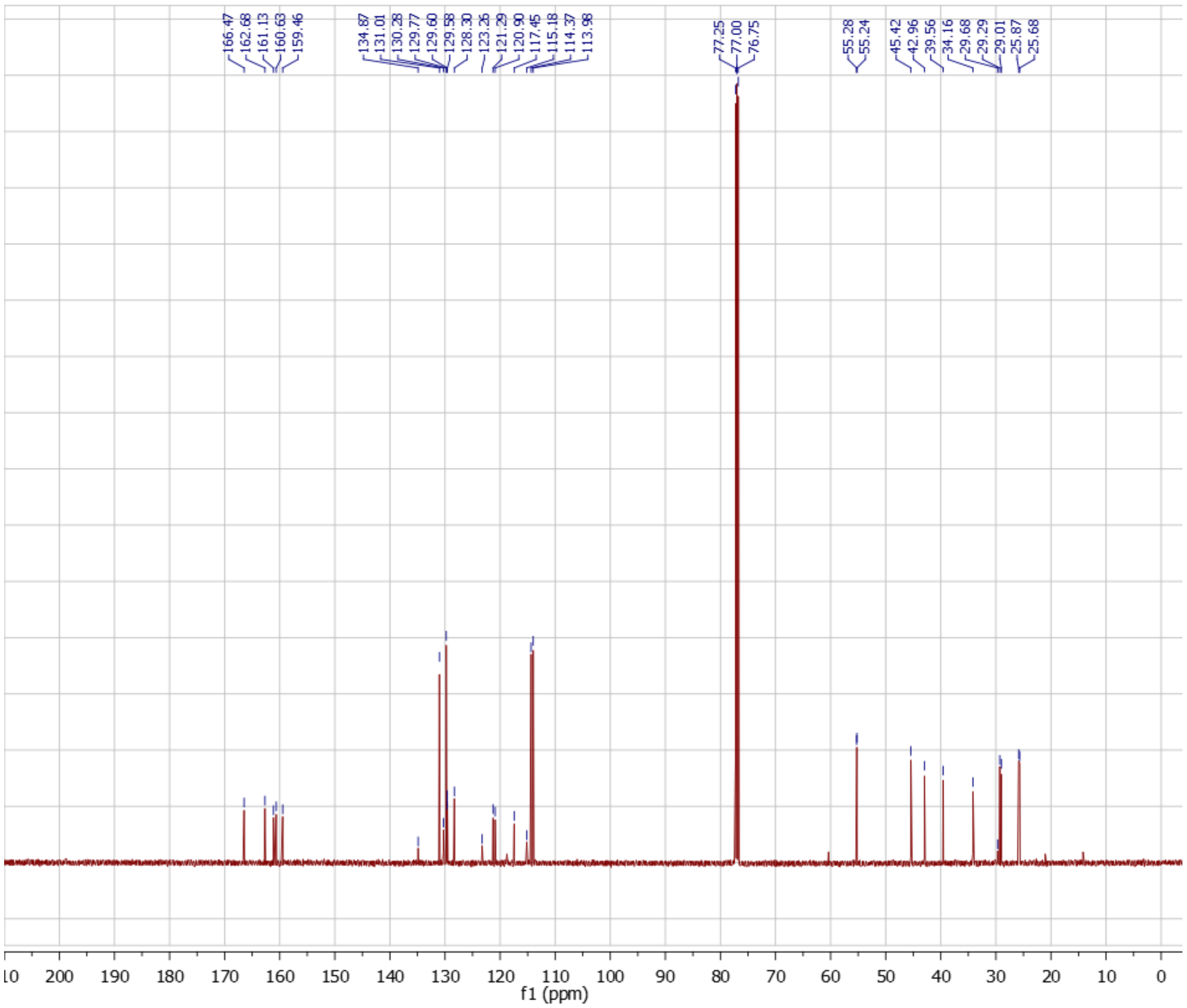

2-[3,4-Bis(4-methoxyphenyl)-5-yl]-N-{4-[(5-dimethylaminonaphthalene)-1-sulfonamido]dodecyl}-acetamide (6b)

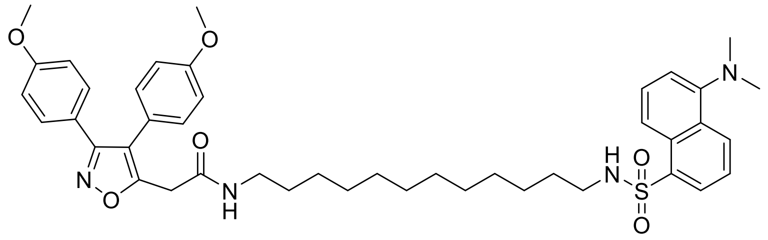

6b ^{1}H-NMR (300 MHz, $CDCl_3$)

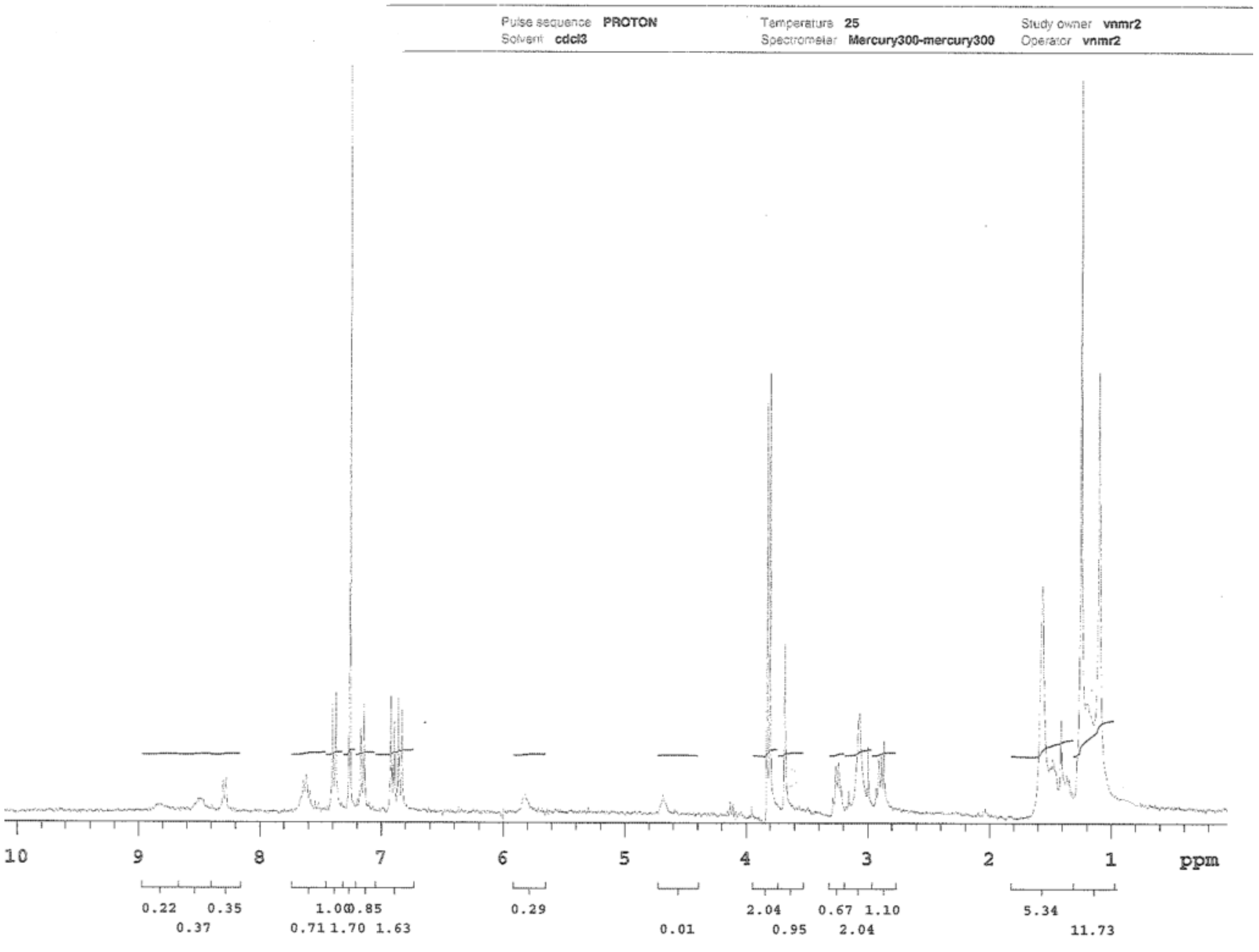

6b ^{13}C-NMR (75 MHz, $CDCl_3$)

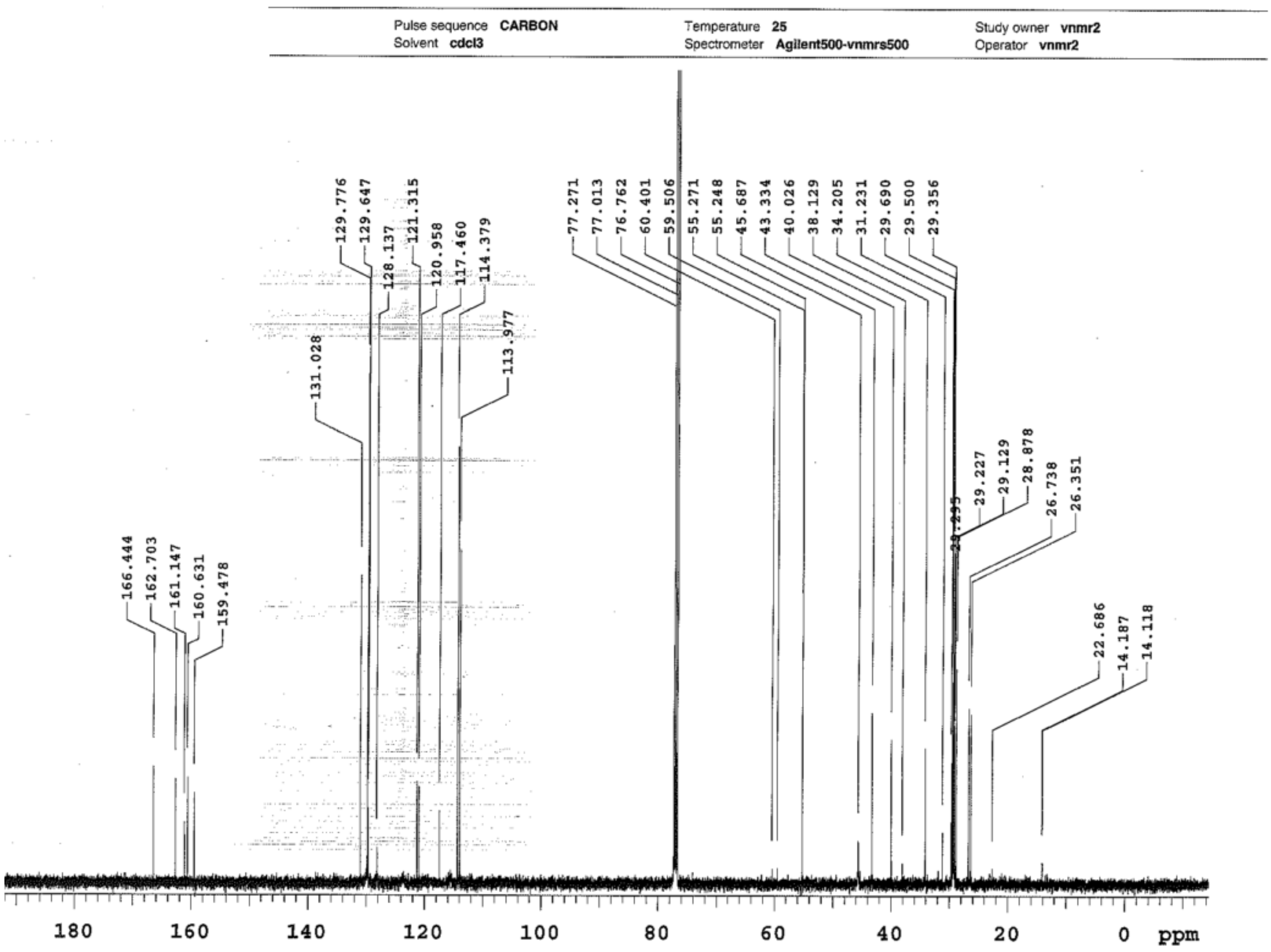

***N*-(4-(2-(3,4-Bis(4-methoxyphenyl)isoxazol-5-yl)acetamido)phenyl)-11-((5-dimethylamino)naphthalene)-1-sulfonamido)undecamide (9)**

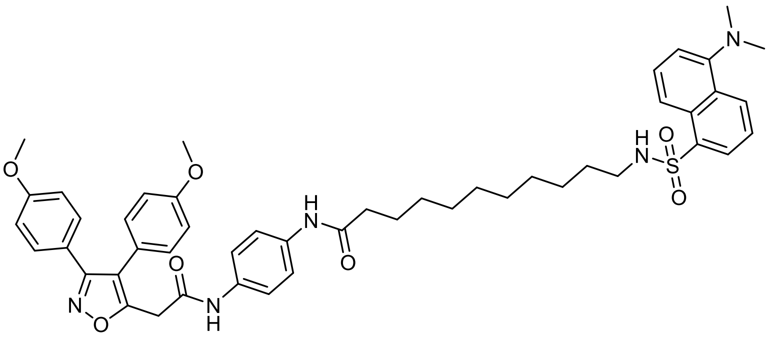

9 ^{1}H-NMR (500 MHz, CD_3OD)

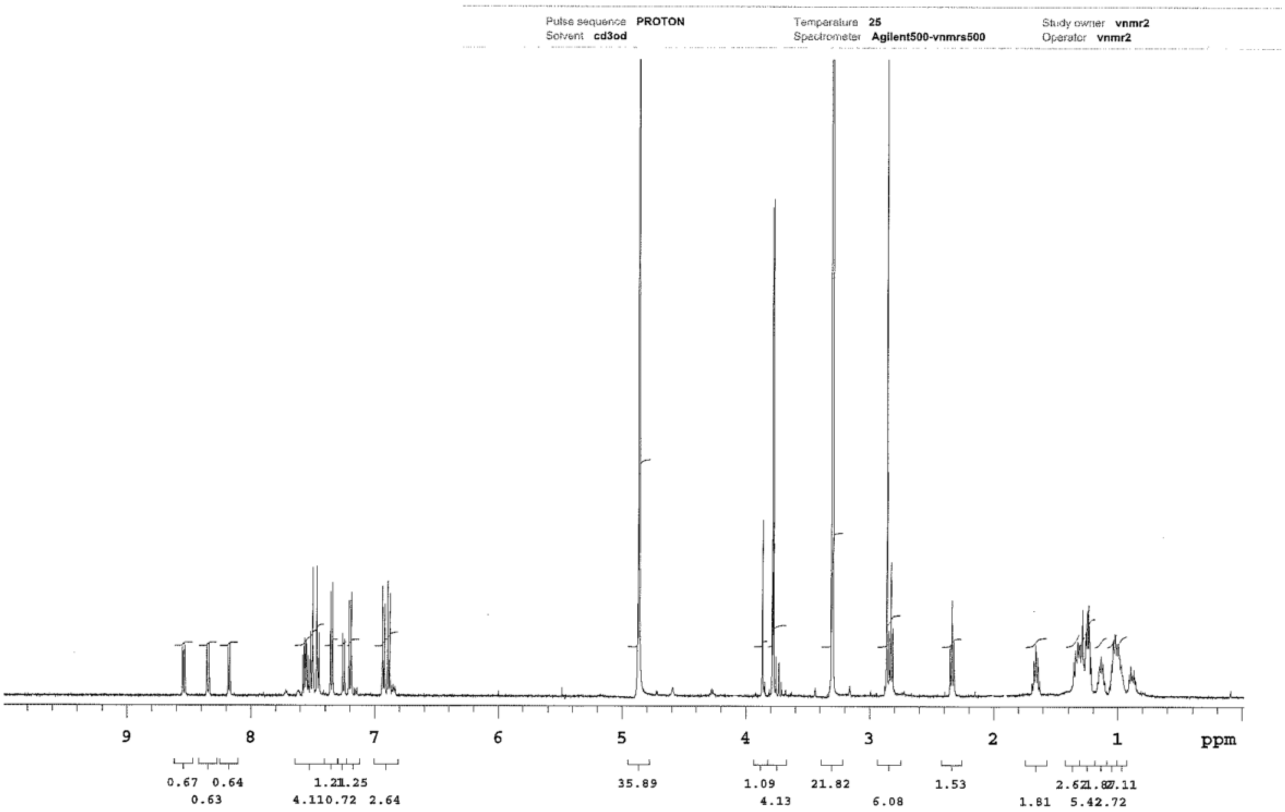

9 ^{13}C-NMR (125 MHz, CD_3OD)

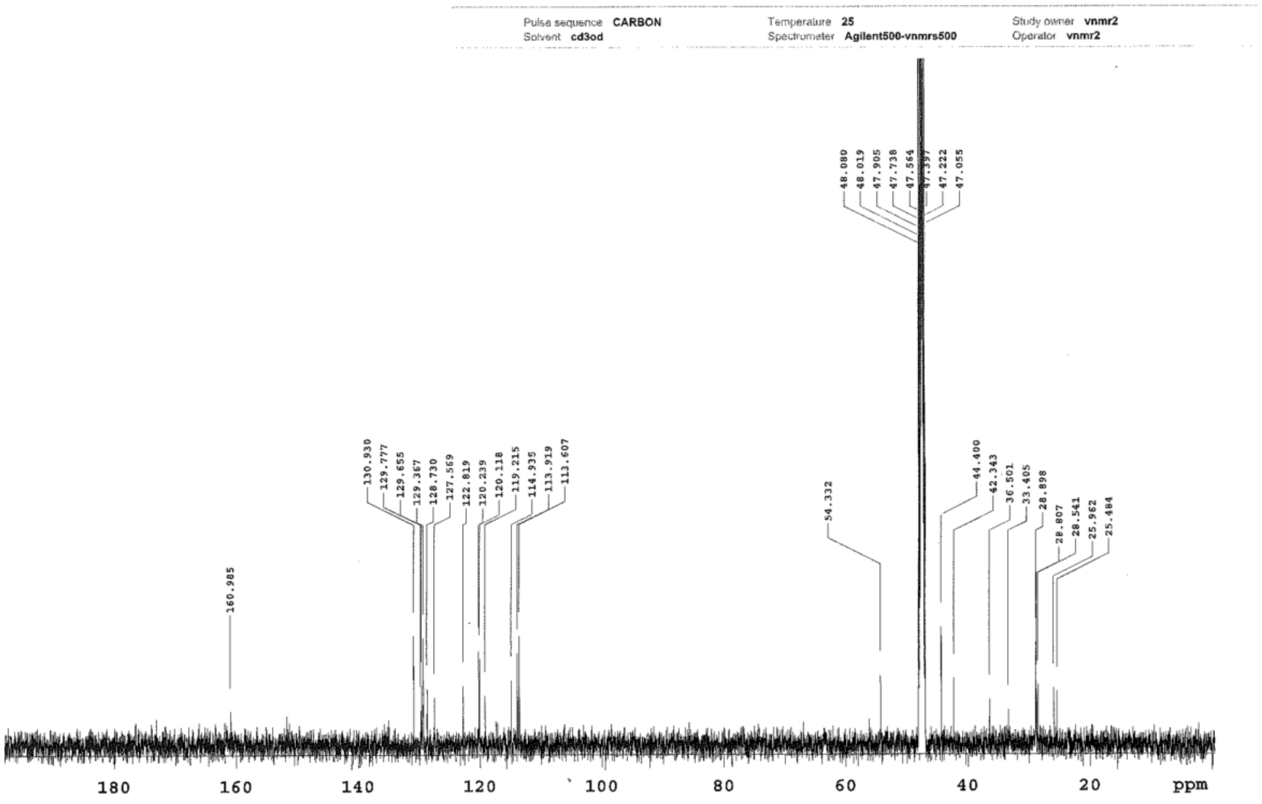

2-(3,4-Bis(4-methoxyphenyl)isoxazol-5-yl)-N-(4-((7-nitrobenzo[c][1,2,5]oxadiazol-4-yl)amino)butyl)acetamide (13a)

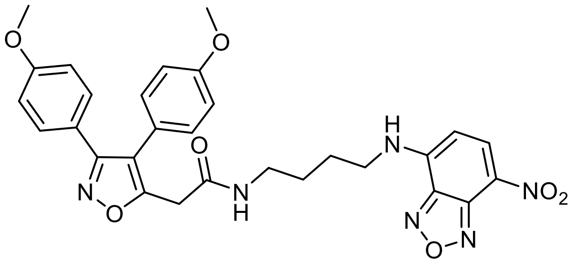

13a ^{1}H-NMR (500 MHz, DMSO-d_6)

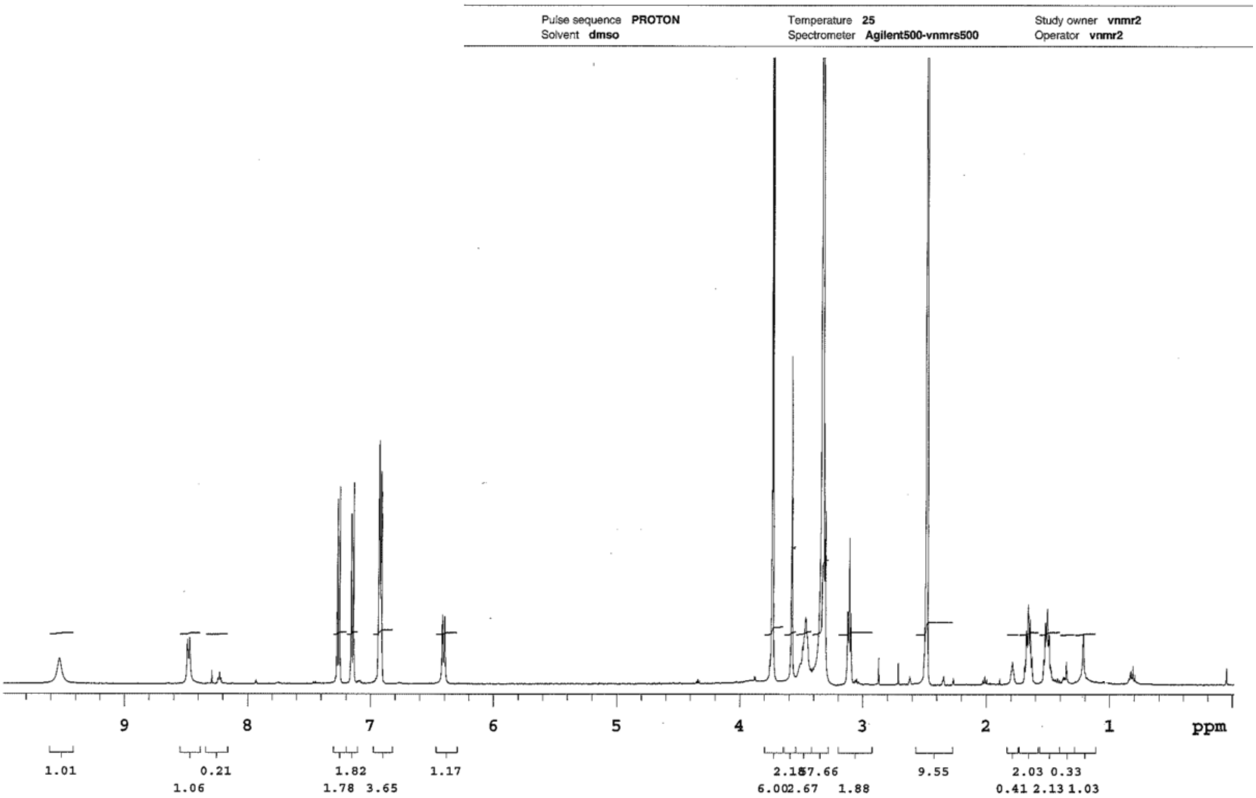

13a ^{13}C-NMR (125 MHz, DMSO-d_6)

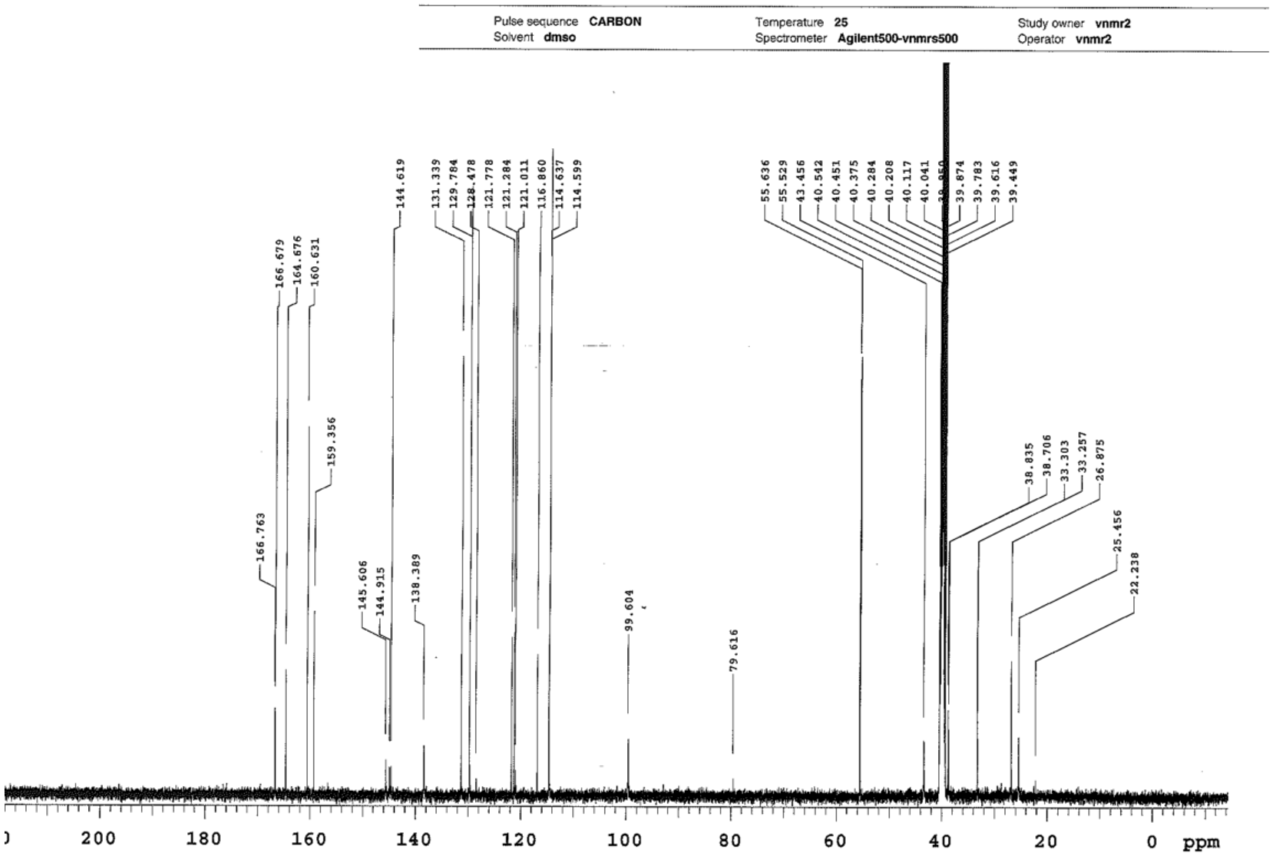

2-(3,4-Bis(4-methoxyphenyl)isoxazol-5-yl)-N-(6-((7-nitrobenzo[c][1,2,5]oxadiazol-4-yl)amino)hexyl)acetamide (13b)

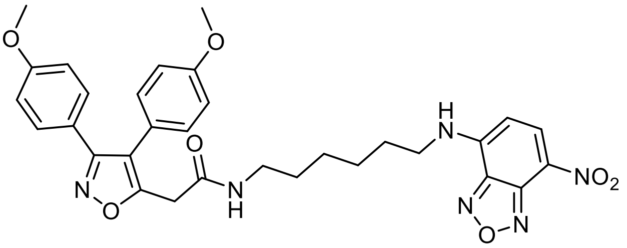

13b ^{1}H-NMR (300 MHz, $CDCl_3$)

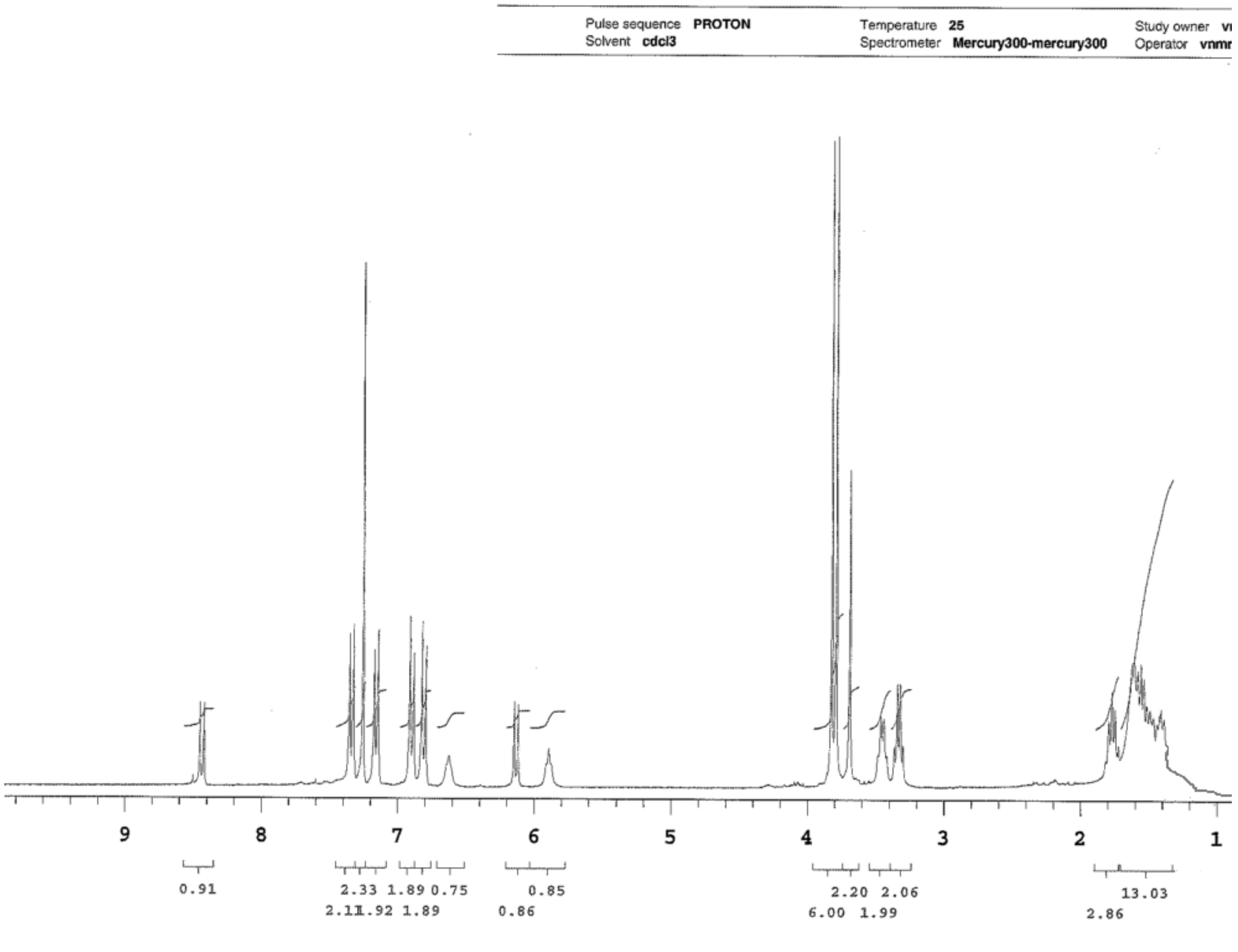

13b ^{13}C-NMR (125 MHz, $CDCl_3$)

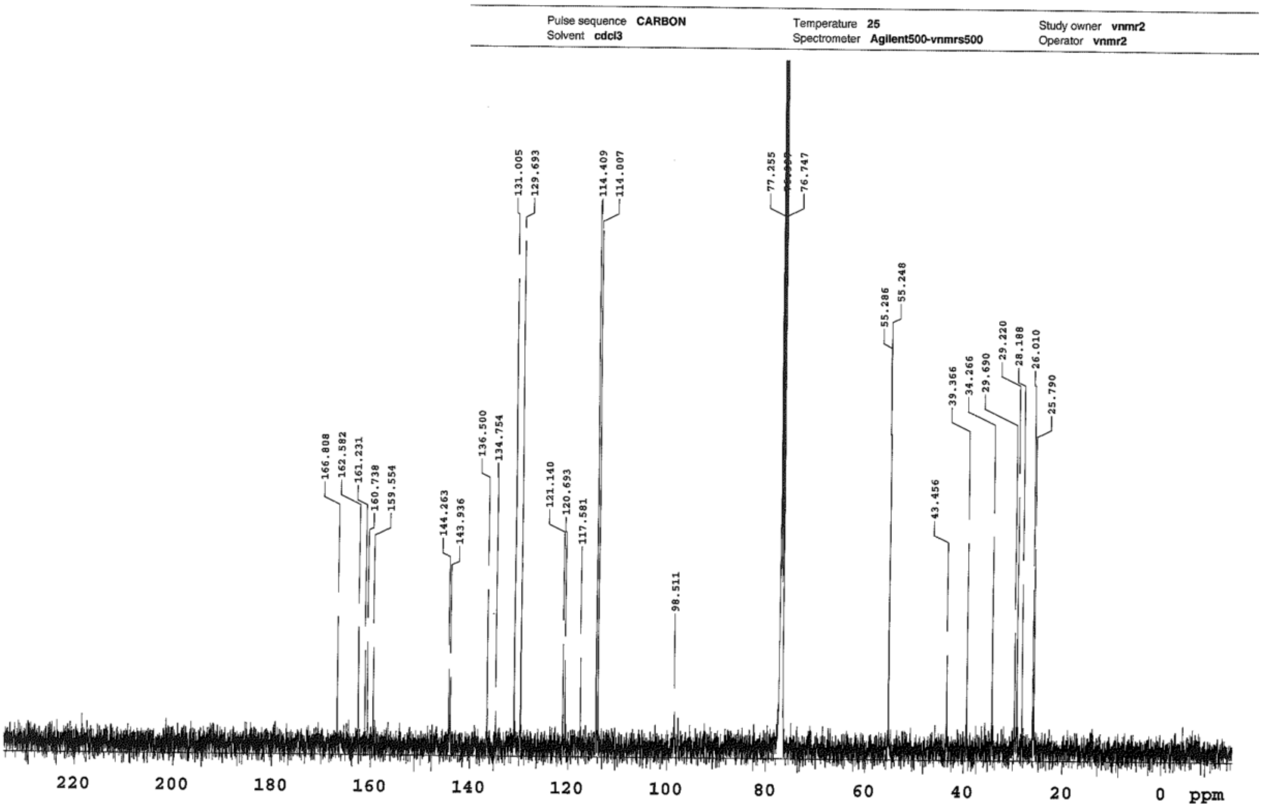

2-(3,4-Bis(4-methoxyphenyl)isoxazol-5-yl)-N-(12-((7-nitrobenzo[c][1,2,5]oxadiazol-4-yl)amino)dodecyl)acetamide (13c)

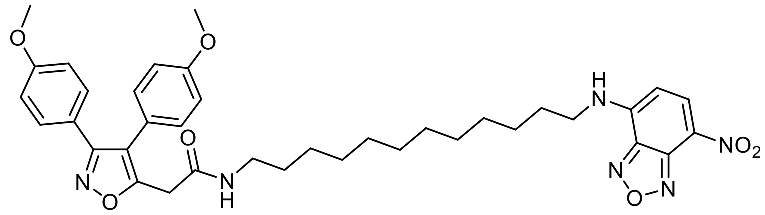

13c ^{1}H-NMR (500 MHz, $CDCl_3$)

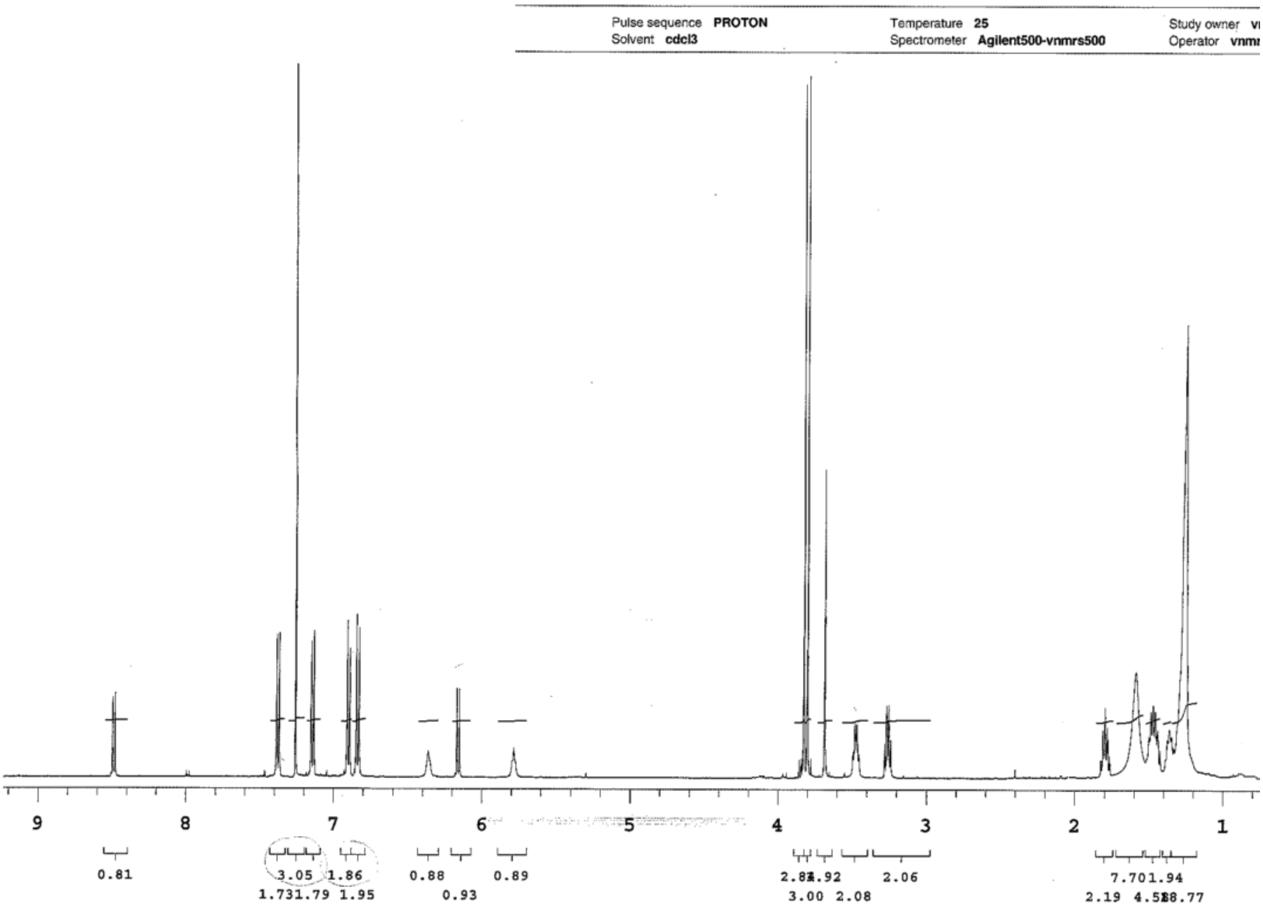

13c ^{13}C-NMR (125 MHz, $CDCl_3$)

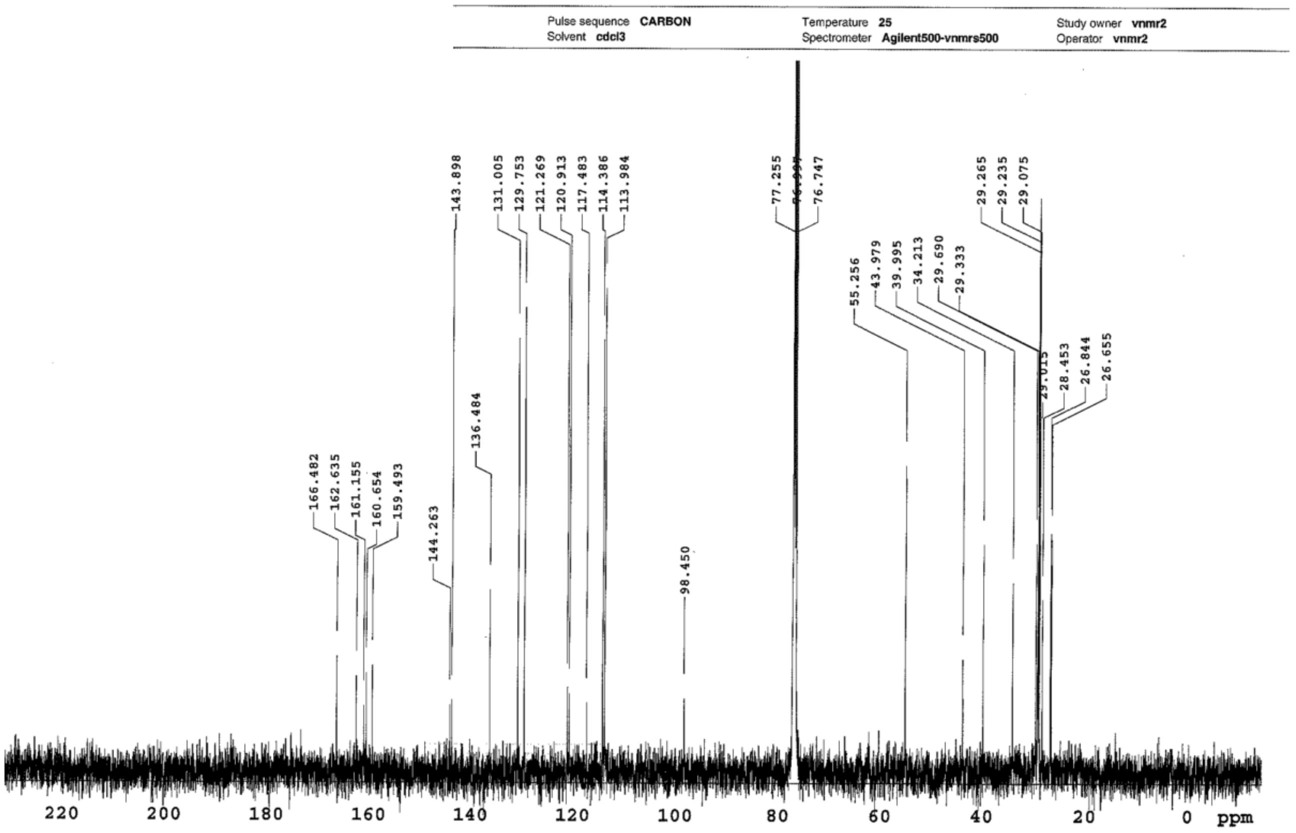

2-(3,4-Bis(4-methoxyphenyl)isoxazol-5-yl)-N-(6-((1,3-dioxo-2-propyl-2,3-dihydro-1H-benzo[de]isoquinolin-6-yl)amino)hexyl)acetamide (17a)

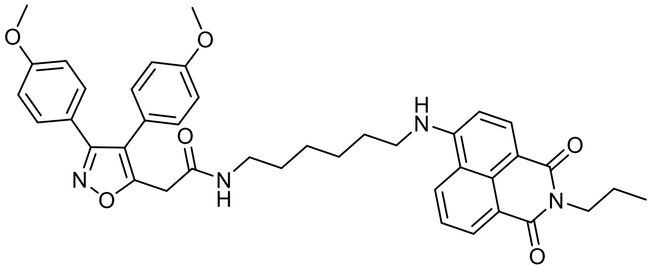

17a ^{1}H-NMR (500 MHz, $CDCl_3$)

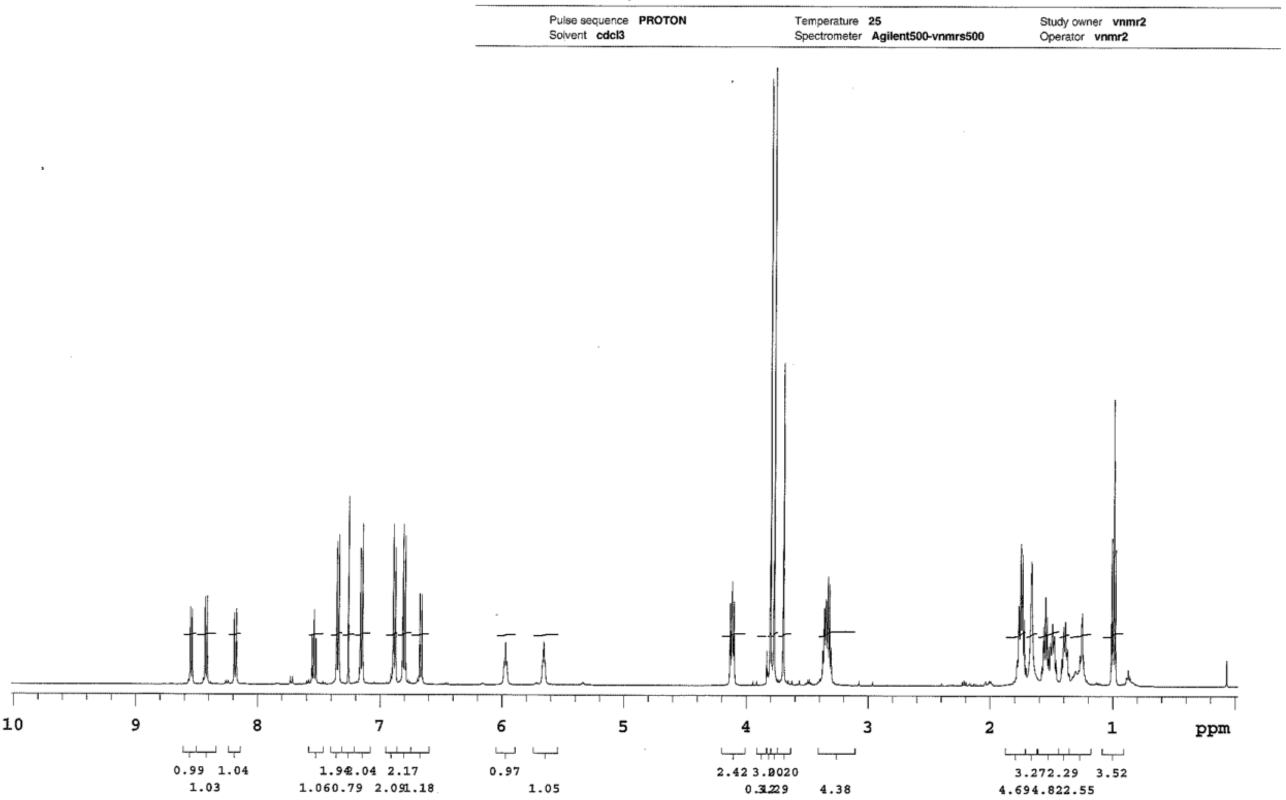

17a ^{13}C NMR (125 MHz, CDCl$_3$)

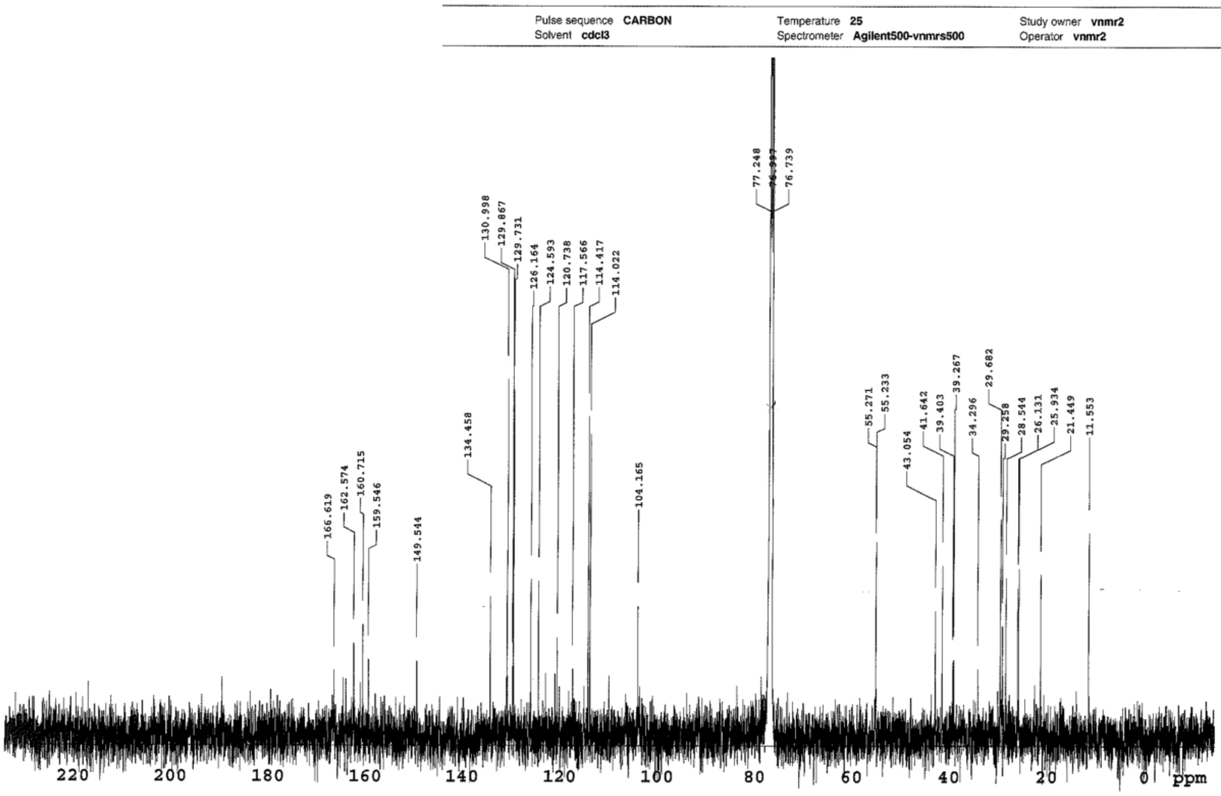

(3,4-Bis(4-methoxyphenyl)isoxazol-5-yl)-N-(4-((1,3-dioxo-2-propyl-2,3-dihydro-1H-benzo[de]isoquinolin-6-yl)amino)phenyl)acetamide (17b)

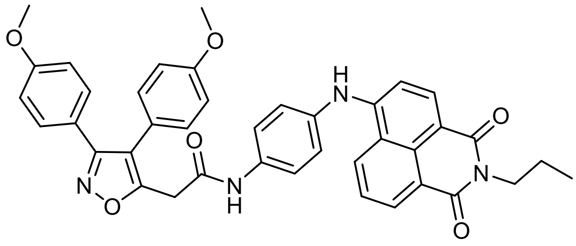

17b ^{1}H-NMR (300 MHz, $CDCl_3$)

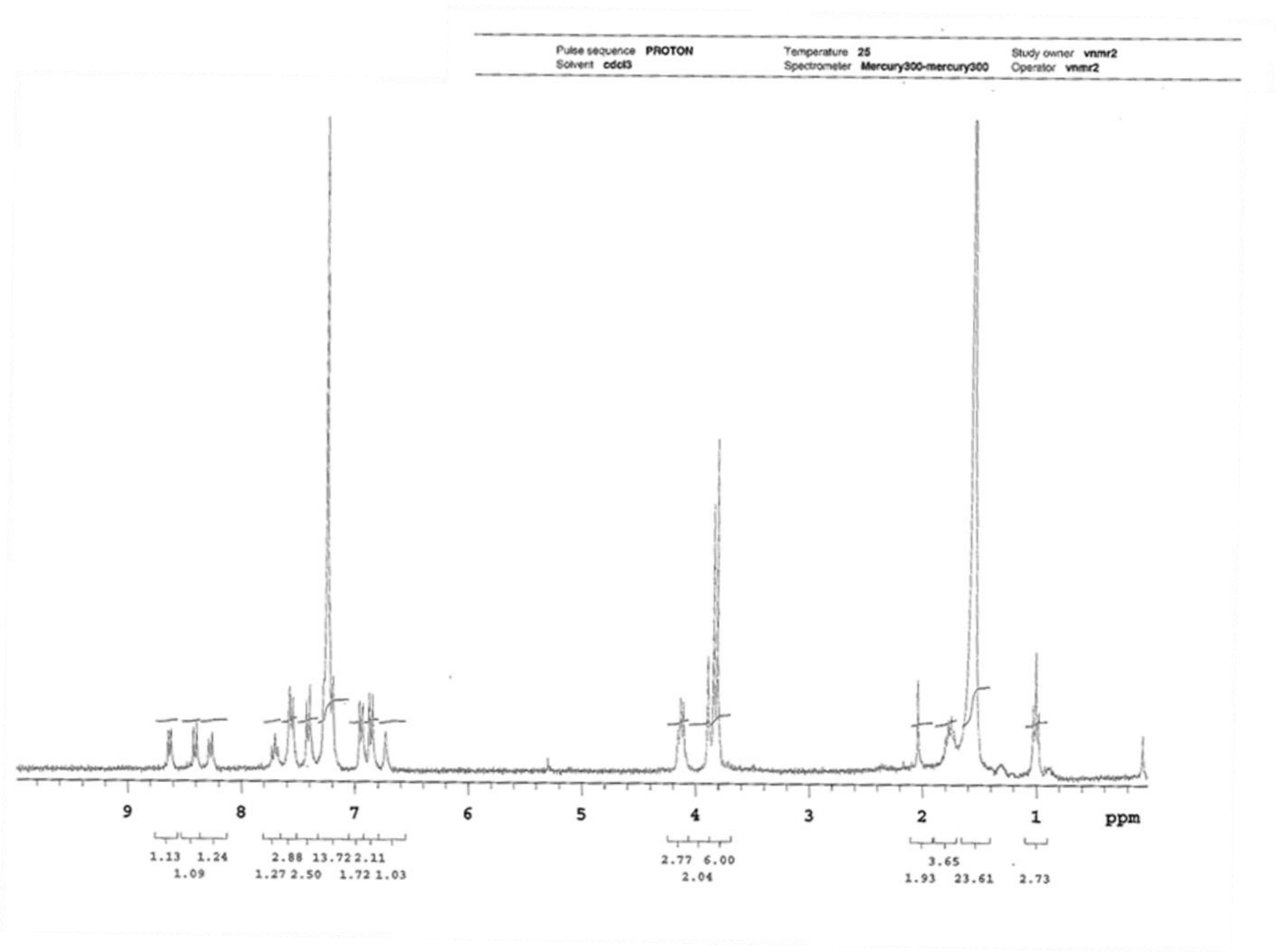

17b ^{13}C-NMR (125 MHz, $CDCl_3$)

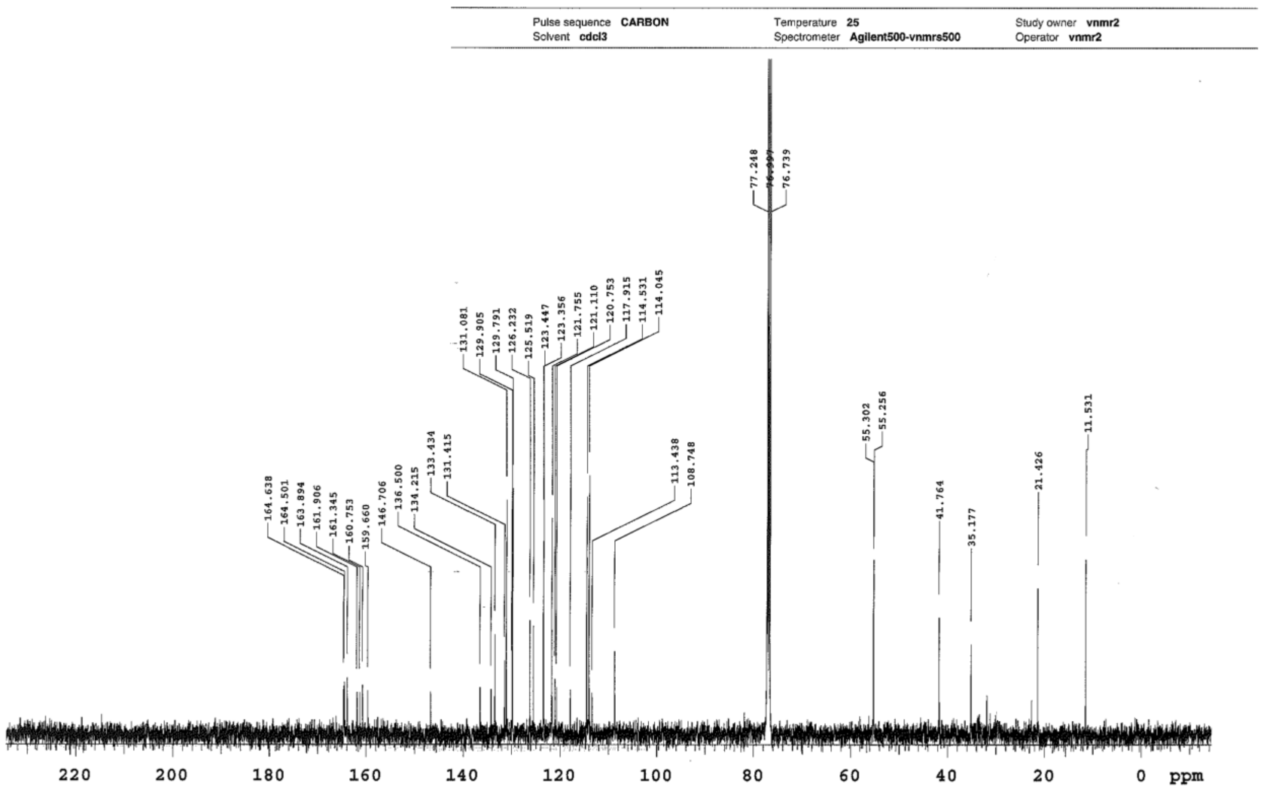

3-(6-(4-(2-(3,4-Bis(4-methoxyphenyl)isoxazole-5-yl)acetamido)butyl)amino-6-oxohexyl)-2-[7-(1,3-Dihydro-1,1-dimethyl-3-ethyl-2H-benz[e]indolin-2-yl-idene)-1,3,5-heptatrienyl]-1,1-dimethyl-3-(6-carboxilato-hexyl)-1H-benz[e]indolium chloride (23)

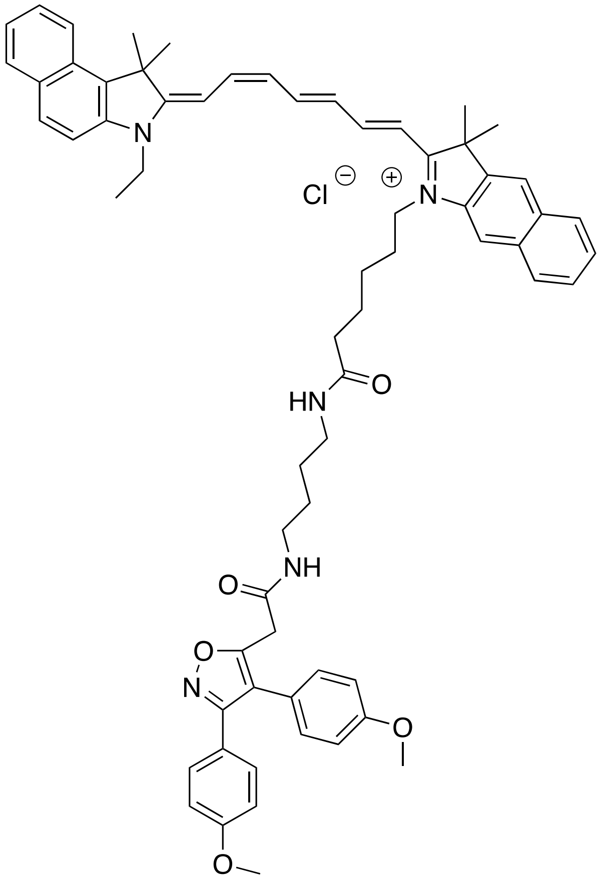

23 (MSA14) ^{1}H-NMR (500 MHz, $CDCl_3$)

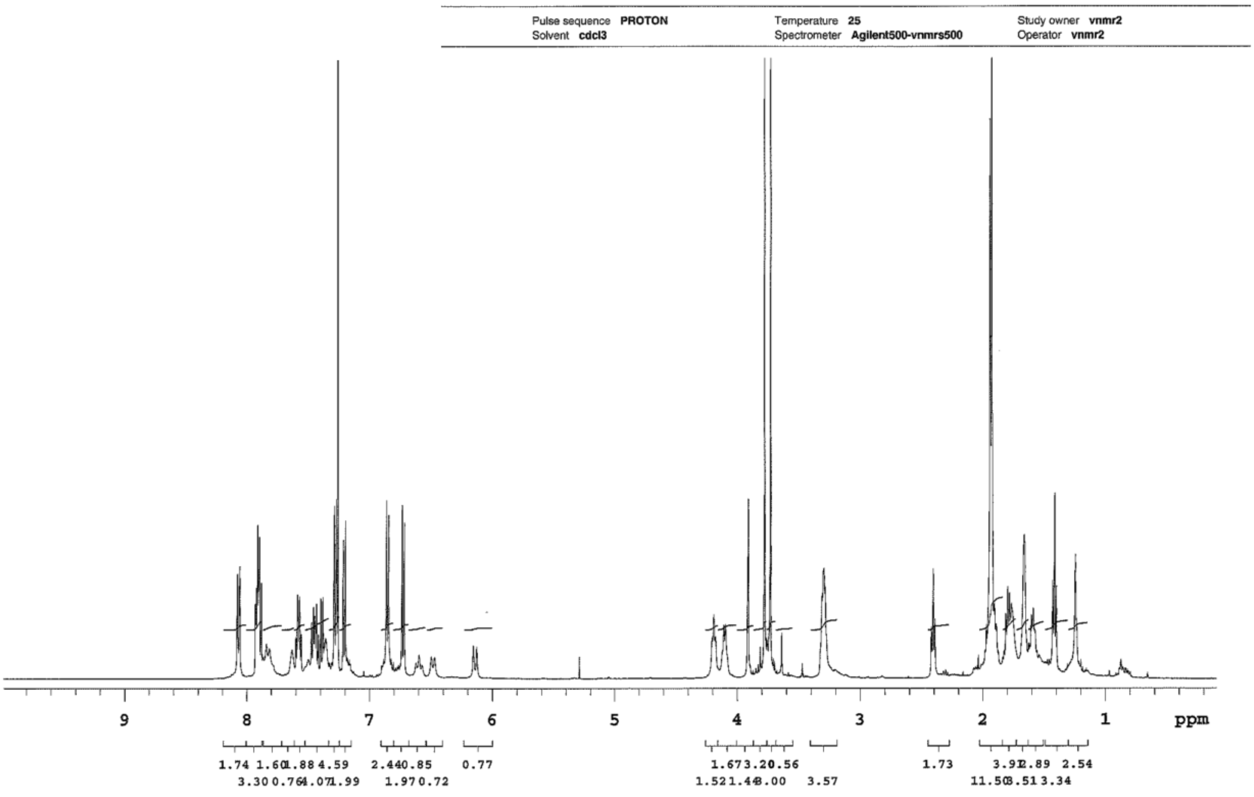

23 (MSA14) ^{13}C-NMR (75 MHz, $CDCl_3$)

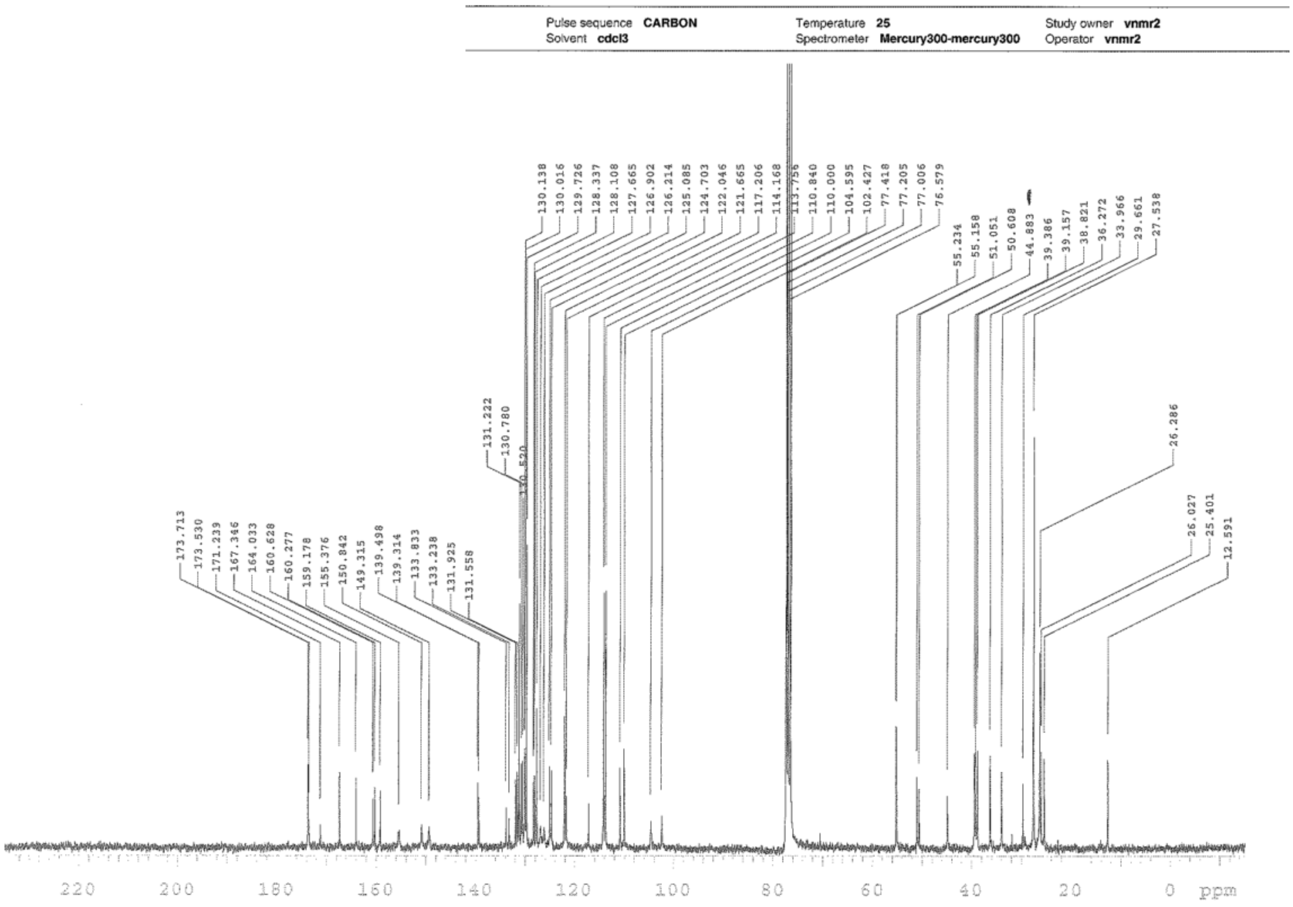

2. HPLC chromatograms of RR11 and 23 (MSA14).

RR11 Rt =3.490 min. (mobile phase: 20 mM NH_4OAc (pH = 5.0)/CH_3CN: 20:80; stationary phase: ZORBAX ECLIPSE Plus C18, analytical 4.6x250mm, 5-Micron); rate = 1mL/min.

```
=====================================================================
Acq. Operator   : SYSTEM                          Seq. Line :   4
Acq. Instrument : 1260                             Location : Vial 22
Injection Date  : 11/6/2020 12:45:20 PM                 Inj :   1
                                                 Inj Volume : 100.000 µl
Sequence File   : C:\Chem32\1\DATA\1-07-2020\MSA 2020-11-06 11-10-37\MSA.S
Method          : C:\CHEM32\1\DATA\1-07-2020\MSA 2020-11-06 11-10-37\MGPISOX80-20.M (
                  Sequence Method)
Last changed    : 11/6/2020 12:42:12 PM by SYSTEM
                  (modified after loading)
```

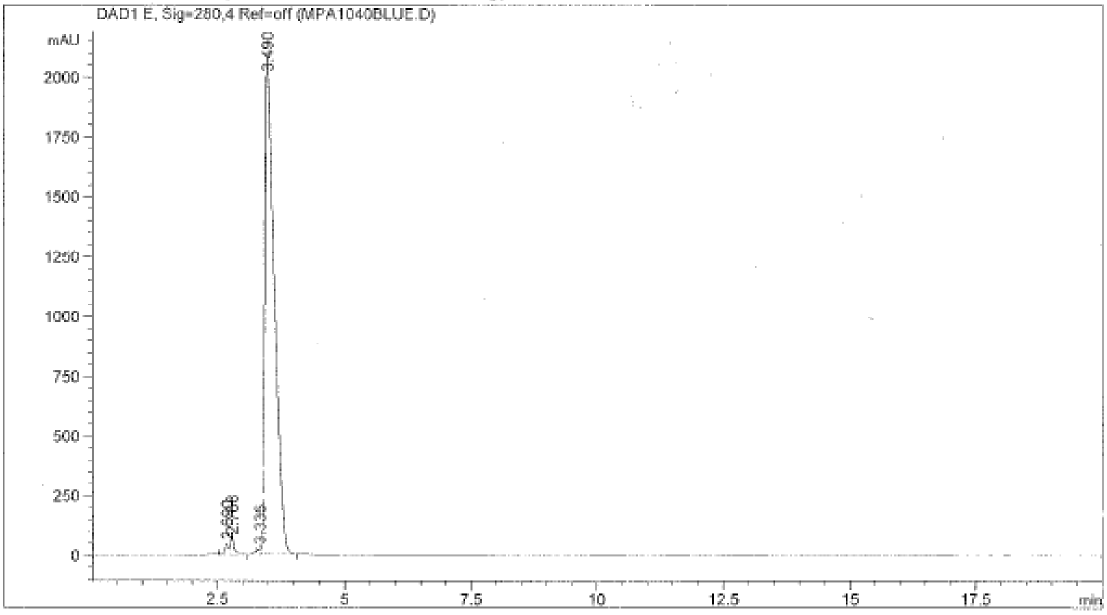


```
=====================================================================
                         Area Percent Report
=====================================================================

Sorted By             :      Signal
Multiplier            :      1.0000
Dilution              :      1.0000
Use Multiplier & Dilution Factor with ISTDs


Signal 1: DAD1 E, Sig=280,4 Ref=off
```

Peak #	RetTime [min]	Type	Width [min]	Area [mAU*s]	Height [mAU]	Area %
1	2.690	VV	0.0716	225.02391	47.24968	0.8261
2	2.788	VB	0.0841	388.98306	66.66299	1.4280
3	3.336	BV	0.0560	60.09843	16.68079	0.2206
4	3.490	VB	0.1772	2.65661e4	2077.43945	97.5253

Totals : 2.72402e4 2208.03291

```
=====================================================================
                         *** End of Report ***
```

23 (MS14) Rt = 6.610 min. (mobile phase: 20 mM NH_4OAc (pH = 5.0)/CH_3CN: 10:90; stationary phase: ZORBAX ECLIPSE Plus C18, analytical 4.6x250mm, 5-Micron); rate = 1mL/min.

```
Acq. Operator   : SYSTEM                          Seq. Line :    4
Acq. Instrument : 1260                             Location : Vial 4
Injection Date  : 7/21/2020 2:11:35 PM                  Inj :    1
                                                 Inj Volume : 10.000 µl
Acq. Method     : C:\CHEM32\1\DATA\ISOXPURITY\GIUGNO2020\1-07-2020\MSA 2020-07-21 11-14-
                  47\MGPISOX90-10.M
Last changed    : 7/21/2020 1:10:15 PM by SYSTEM
                  (modified after loading)
Analysis Method : C:\CHEM32\1\DATA\ISOXPURITY\GIUGNO2020\1-07-2020\MSA 2020-07-21 11-14-
                  47\MGPISOX90-10.M (Sequence Method)
Last changed    : 12/23/2021 10:44:40 AM by SYSTEM
                  (modified after loading) (Current integration events modified)
```

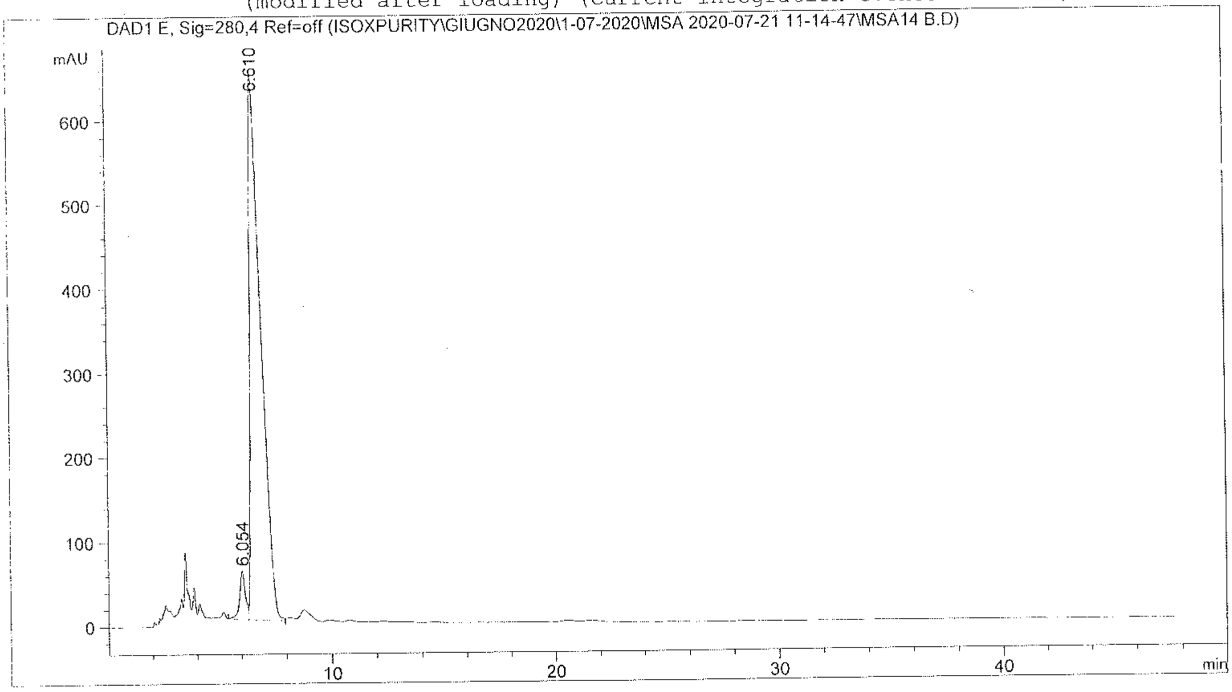


```
Area Percent Report

Sorted By          :     Signal
Multiplier         :     1.0000
Dilution           :     1.0000
Use Multiplier & Dilution Factor with ISTDs

Signal 1: DAD1 E, Sig=280,4 Ref=off
```

Peak #	RetTime [min]	Type	Width [min]	Area [mAU*s]	Height [mAU]	Area %
1	6.054	BV	0.2539	1033.15100	57.97709	4.4366
2	6.610	VV	0.4933	2.22540e4	646.92200	95.5634
Totals :				2.32871e4	704.89908	

3. FLAP S1-S7 figures of selected target compounds.

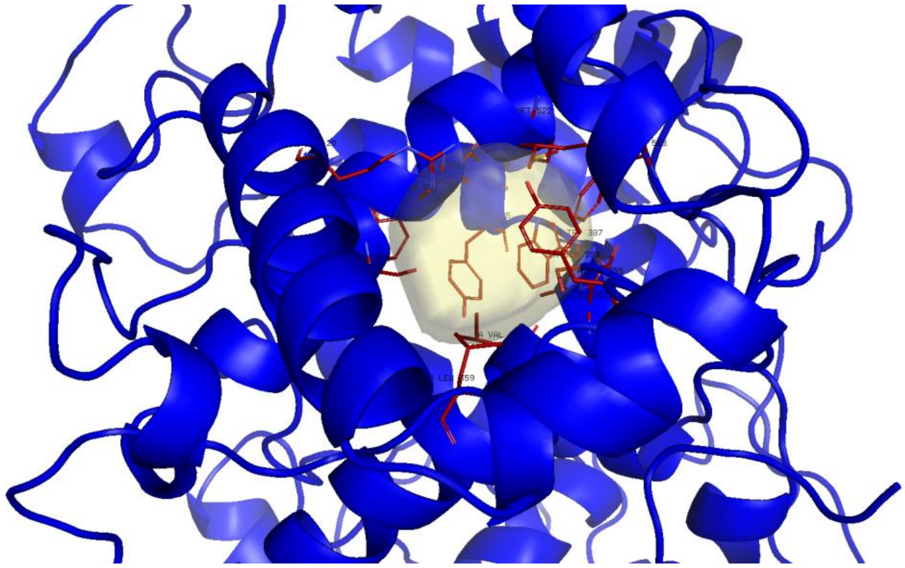

a)

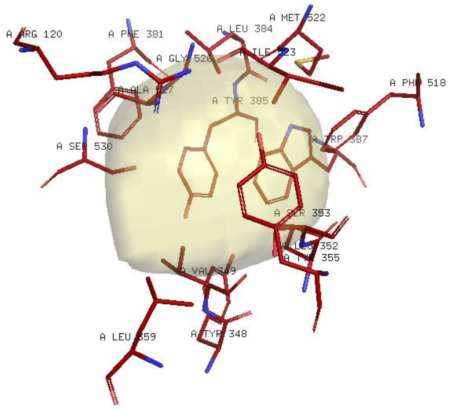

b)

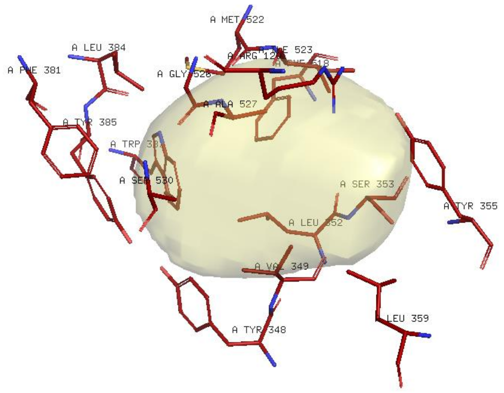

c)

Figure S1. a) The top-ranked pocket (front view, P1) overlaid with the *h*COX-1 X-ray structures (PDB code 6Y3C); b), c) zoom of the substrate binding site and involved amino acids (R120 Y348 V349 L352 S353 Y355 L359 F381 L384 Y385 W387 F518 M522 I523 G526 A527 S530).

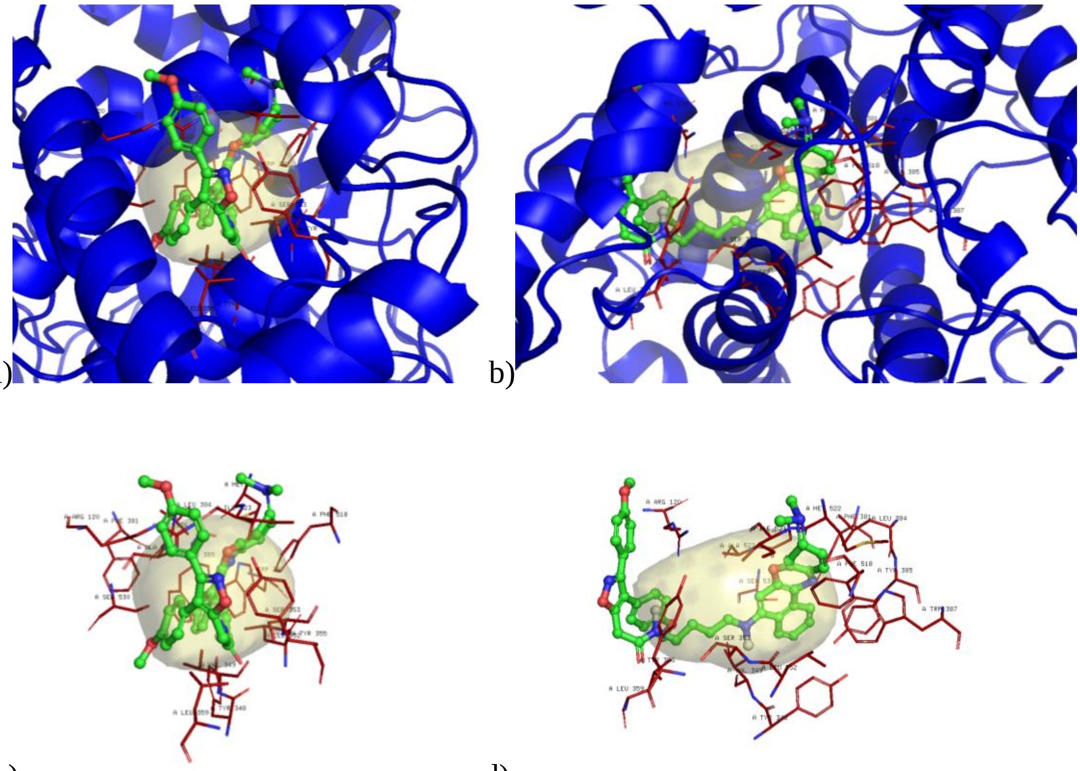

a) b) c) d)

Figure S2. 3D binding mode of reference compound **RR11** inside the substrate binding site of *h*COX-1 (PDB code 6y3c) determined by FLAP analysis, front (a, c) and side view (b, d).

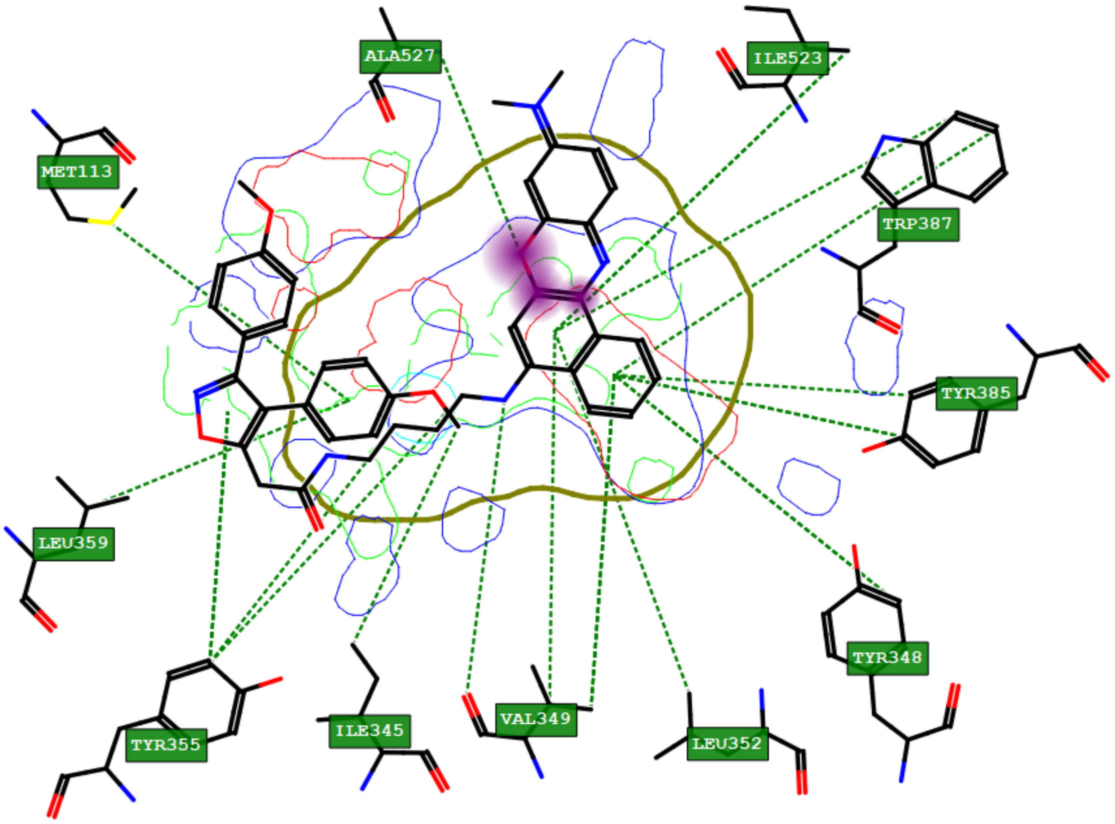

Figure S3. 2D FLAP binding poses of reference compound **RR11** inside the substrate binding site of *h*COX-1 (PDB code 6y3c). Residues located in the *h*COX-1 binding site are highlighted in stick-mode: the most important amino acids are shown together with their respective numbers. Purple regions indicate the strong interactions. Colored lines represent the area of hydrogen-bonding donor interactions (red), hydrogen-bonding acceptor interactions (blue), and the hydrophobic interactions (green).

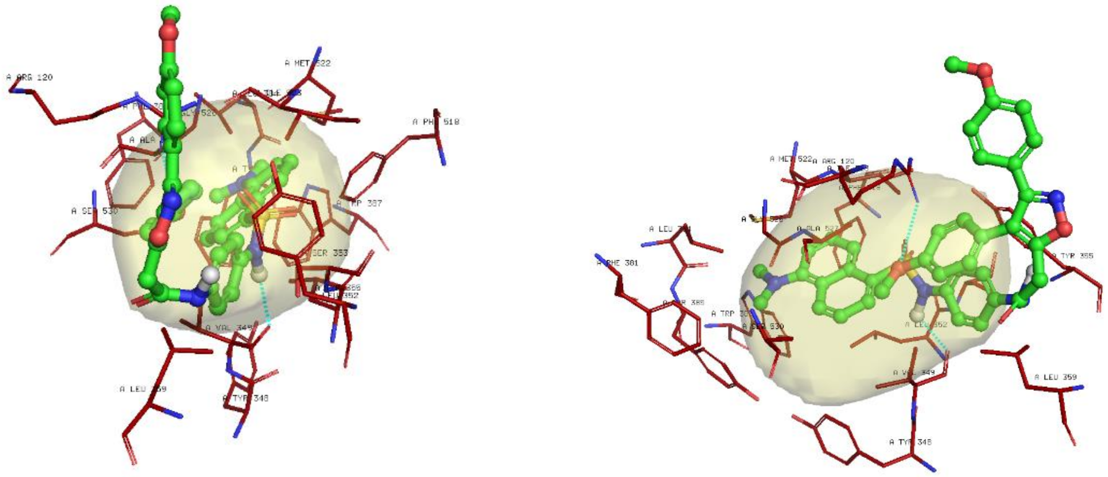

a) b)

Figure S4. 3D binding mode of **3b** inside the substrate binding site of *h*COX-1 (PDB code 6y3c) determined by FLAP analysis, front (a) and side view (b).

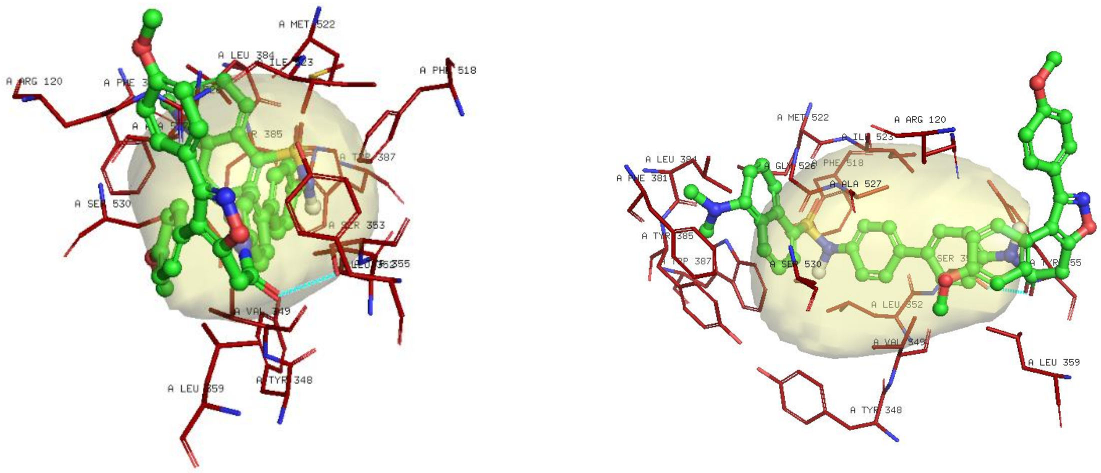

a) b)

Figure S5. 3D binding mode of reference compound **3c** inside the substrate binding site of *h*COX-1 (PDB code 6y3c) determined by FLAP analysis, front (a) and side view (b).

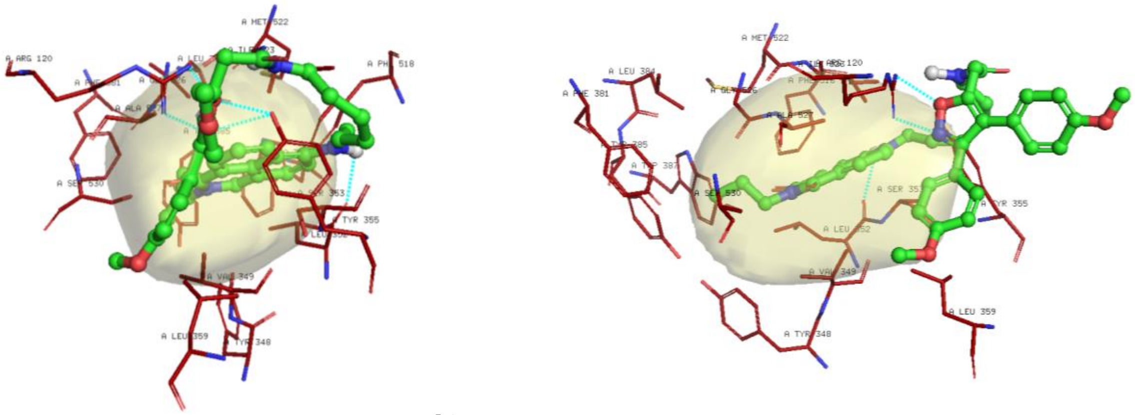

a) b)

Figure S6. 3D binding mode of **17a** inside the substrate binding site of *h*COX-1 (PDB code 6y3c) determined by FLAP analysis, front (a) and side view (b).

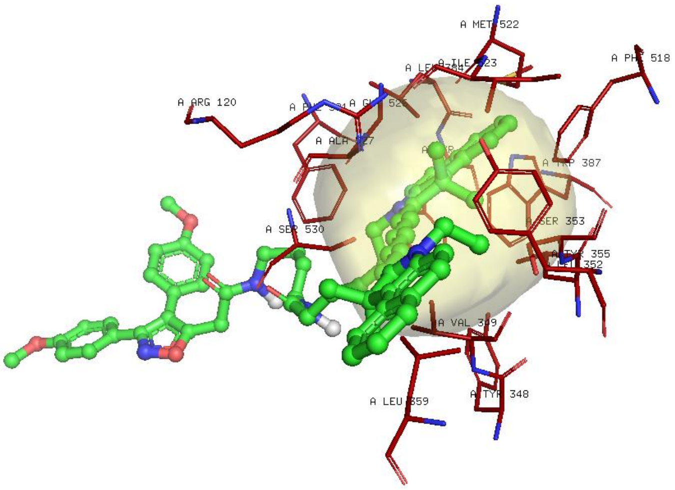

a)

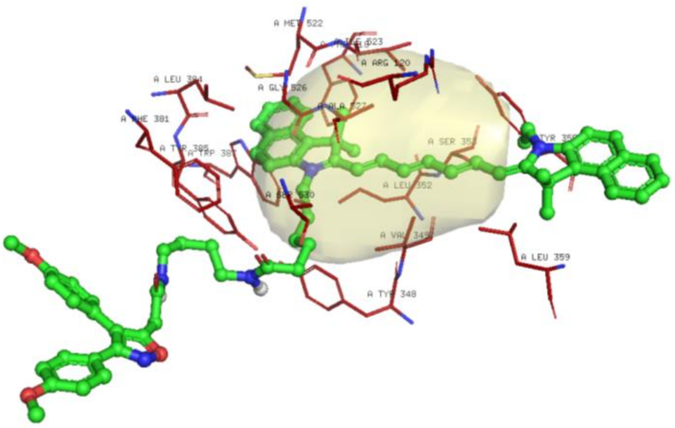

b)

Figure S7. 3D binding mode of **23** inside the substrate binding site of *h*COX-1 (PDB code 6y3c) determined by FLAP analysis, front (a) and side view (b).
